# Innate immune imprints shape HIV-1 reservoir cell persistence during long-term antiretroviral therapy

**DOI:** 10.1038/s41590-026-02637-w

**Published:** 2026-09-01

**Authors:** Toong Seng Tan, WeiWei Sun, Ce Gao, Mathias Viard, Lucy C. Walters, Melanie Lancien, Yuko Yuki, Tram N. Van, Carlos Casquero, Xuan Guo, Rebecca Hoh, David W. Haas, Nelson Michael, Gregory D. Kirk, George Yendewa, Rajesh T. Gandhi, Seble G. Kassaye, Phyllis C. Tien, Bruce D. Walker, Michael J. Peluso, Jeffrey M. Jacobson, Steven G. Deeks, Mary Carrington, Xu G. Yu, Mathias Lichterfeld

**Affiliations:** 1 Ragon Institute of MGB, MIT and Harvard, Cambridge, MA USA; 2 Infectious Disease Division, Brigham and Women’s Hospital, Boston, MA USA; 3 Basic Science Program, Frederick National Laboratory for Cancer Research, National Cancer Institute, Frederick, MD USA; 4 Laboratory of Integrative Cancer Immunology, Center for Cancer Research, National Cancer Institute, Bethesda, MD USA; 5 Koch Institute for Integrative Cancer Research, Massachusetts Institute of Technology, Cambridge, MA USA; 6 Division of HIV, Infectious Diseases and Global Medicine, University of California, San Francisco, San Francisco, CA USA; 7 Vanderbilt University Medical Center, Nashville, TN USA; 8 U.S. Military HIV Research Program, Silver Spring, MD USA; 9 Johns Hopkins University School of Public Health, Baltimore, MD USA; 10 School of Medicine, Case Western Reserve University, Cleveland, OH USA; 11 Infectious Disease Division, Massachusetts General Hospital, Boston, MA USA; 12 Department of Medicine, Georgetown University Medical Center, Washington DC, USA; 13 Department of Medicine, University of California, San Francisco, San Francisco, CA USA; 14 Department of Veterans Affairs Medical Center, San Francisco, CA USA; 15 Howard Hughes Medical Institute, Chevy Chase, MD USA; 16 Institute for Medical Engineering and Sciences, Massachusetts Institute of Technology, Cambridge, MA USA; 17 Department of Biology, Massachusetts Institute of Technology, Cambridge, MA USA

**Keywords:** Immunogenetics, Viral immune evasion, Innate immunity, HIV infections

## Abstract

Host immune responses that can target and eliminate HIV-1 reservoir cells during suppressive antiretroviral therapy are poorly understood. Here, analyzing over 6,000 proviral DNA amplicons from 104 individuals on long-term antiretroviral therapy, we found that carriers of HLA-C2 allotypes, which promote NK cell education via interactions with KIR2DL1, exhibited lower frequencies of intact proviruses. No protective effects of HLA-C2 alleles were observed during untreated infection, suggesting a selective vulnerability of reservoir cells to NK-cell-mediated immune activity under antiretroviral therapy. Supporting this, frequencies of KIR2DL1^+^ NK cells, particularly those coexpressing the activating HLA-E receptor NKG2C, were inversely correlated with frequencies of intact proviruses. Moreover, viral protein U variants that strongly downregulate HLA-C, increasing a missing-self response by KIR2DL1^+ ^NK cells, were associated with smaller reservoirs in carriers of HLA-C2. Infected cells surviving long-term displayed elevated HLA-C expression, consistent with in vivo selection for resistance to KIR2DL1^+ ^NK-cell-mediated immune clearance. Immune activity of KIR2DL1^+^ NK cells against viral reservoir cells was enhanced by NKG2C–HLA-E interactions, as sequence variants in HLA-E-restricted HIV-1 epitopes reduced NKG2C-dependent NK cell activation and were associated with higher frequencies of intact proviruses. These findings underscore the critical influence of host and viral genetic variation on reservoir persistence and highlight opportunities for leveraging innate immunity in HIV cure strategies.

## Abstract (web summary)

HIV reservoir cells are traditionally considered invisible to host immune responses due to viral latency. This work challenges this view by demonstrating that HIV persistence during long-term antiretroviral therapy is strongly influenced by KIR2DL1-educated NK cells.

## Main

Rebound-competent HIV-1-infected cells can persist life-long despite currently available antiretroviral therapy (ART), and represent the major barrier to the development of curative treatment interventions for HIV-1 infection^1–3^. The majority of these persisting viral reservoir cells include memory CD4^+^ T cells that harbor a chromosomally integrated form of viral DNA as a ‘provirus’, but express limited viral RNA and effectively resist immune elimination, for reasons that are currently poorly understood^4^. Nevertheless, several recent experimental observations suggest that the human immune system can recognize and target some of these reservoir cells, likely due to ongoing low-level viral transcription^5–9^ during ART. Indeed, imprints of host immune selection pressure can be detected through deep interrogations of the viral reservoir cell pool, specifically when focusing on genome-intact proviruses, which decay faster after ART initiation^10,11^ and appear more susceptible to host immune activity. In particular, the profiling of chromosomal integration sites has indicated the selective persistence of genome-intact proviruses in heterochromatin locations, likely as a result of immune-mediated selection pressure that preferentially targets more transcriptionally active proviruses integrated into accessible chromatin^7,12–14^. Moreover, single-cell proteogenomic profiling studies suggested that, relative to the total memory CD4^+^ T cell pool, persisting viral reservoir cells express higher levels of inhibitory immunoregulatory markers^15^, a finding that is best explained by effective immune targeting of reservoir cells with a phenotypic profile more conducive to antiviral immune activity. The types of host immune responses that can effectively drive immune selection of HIV-1 reservoir cells, however, are unclear at present. Cytotoxic T cells can eliminate infected cells when presenting viral peptides on human leukocyte antigen (HLA) molecules and have a recognized role in antiviral immunity during untreated HIV-1 infection^16^, especially in persons with natural, drug-free control of HIV-1 replication^17–19^; however, their ability to target HIV-1 reservoir cells during ART is less well understood^20^. Natural killer (NK) cells preferentially recognize and kill HLA-deficient target cells, providing a complementary mode of immune surveillance. NK cells undergo education through interactions between inhibitory killer-cell immunoglobulin-like receptors (KIR) and corresponding HLA class I ligands^21^ and can contribute to antiviral immune defense in untreated HIV-1 infection^22,23^; however, recent studies also suggest their involvement in immune activity against viral reservoirs during ART^24–26^, and during drug-free HIV-1 control following ART interruption in rare ‘posttreatment controllers’^27^. Here we conducted a detailed characterization of proviral sequences and host immune responses in a large cohort of people living with HIV-1 on long-term ART to define immune mechanisms that can impose selection pressure against the HIV-1 reservoir cell pool.

## Results

### Lower HIV-1 reservoirs in carriers of HLA-C2 alleles

**Fig. 1:**
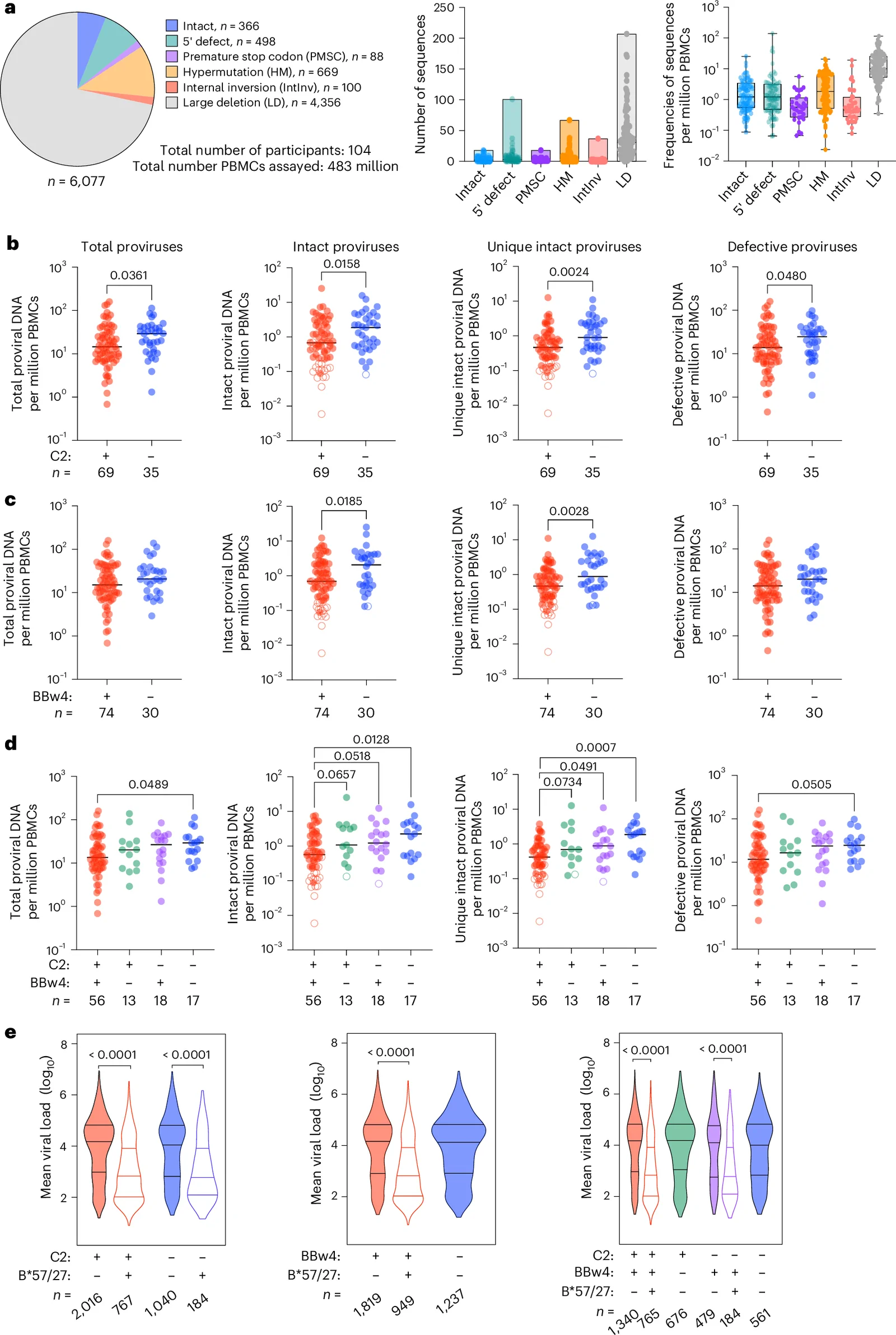
HLA-C2 and BBw4 are associated with reduced frequencies of intact proviruses during long-term ART. **a**, Proviral HIV-1 sequence landscape in *n* = 104 study persons on suppressive ART. Data show proportions (left), absolute numbers (middle) and relative frequencies of indicated HIV-1 DNA sequences (right). Each point represents one individual; boxes indicate median and interquartile range; whiskers extend to minimum and maximum values. PMSC, premature stop codon; HM, hypermutation; IntInv, internal inversion; LD, large deletion. **b**,**c**, Frequencies of indicated HIV-1 proviruses in study participants on long-term ART carrying or not carrying HLA-C2 (**b**) or HLA-B Bw4 alleles (**c**). **d**, Frequencies of indicated HIV-1 proviruses in participants on long-term ART carrying both HLA-C2 and HLA-B Bw4 alleles, in individuals lacking both alleles, and those with either HLA-C2 or HLA-B Bw4 alone. When members of clones of intact proviruses were collapsed and counted as a single provirus, they are referred to as ‘unique intact proviruses’. Open circles represent data at the limit of detection, defined as 0.5 viral copies in the maximum number of cells analyzed without target identification. Horizontal lines reflect the median. Significance was tested using two-tailed Mann–Whitney *U* tests for comparisons of two groups (**b**,**c**) and Kruskal–Wallis with Dunn’s post-hoc test for multiple-group comparisons (**d**), nominal *P* values are shown. **e**, Violin plots (indicating median, 25% and 75% percentiles) reflecting plasma viremia in untreated individuals expressing at least one HLA-C2 allele (left), one HLA-B Bw4 allele (middle) or indicated combinations of C2 and BBw4 alleles (right). Data for carriers of the protective HLA-B*57 and HLA-B*27 alleles are shown separately. Two untreated individuals with atypical B*57 or B*27 alleles that are not Bw4 carriers were excluded from the violin plots analysis in the middle and right panels. Significance was tested using two-tailed Mann–Whitney *U* tests. Source data

**Extended Data Fig. 1:**
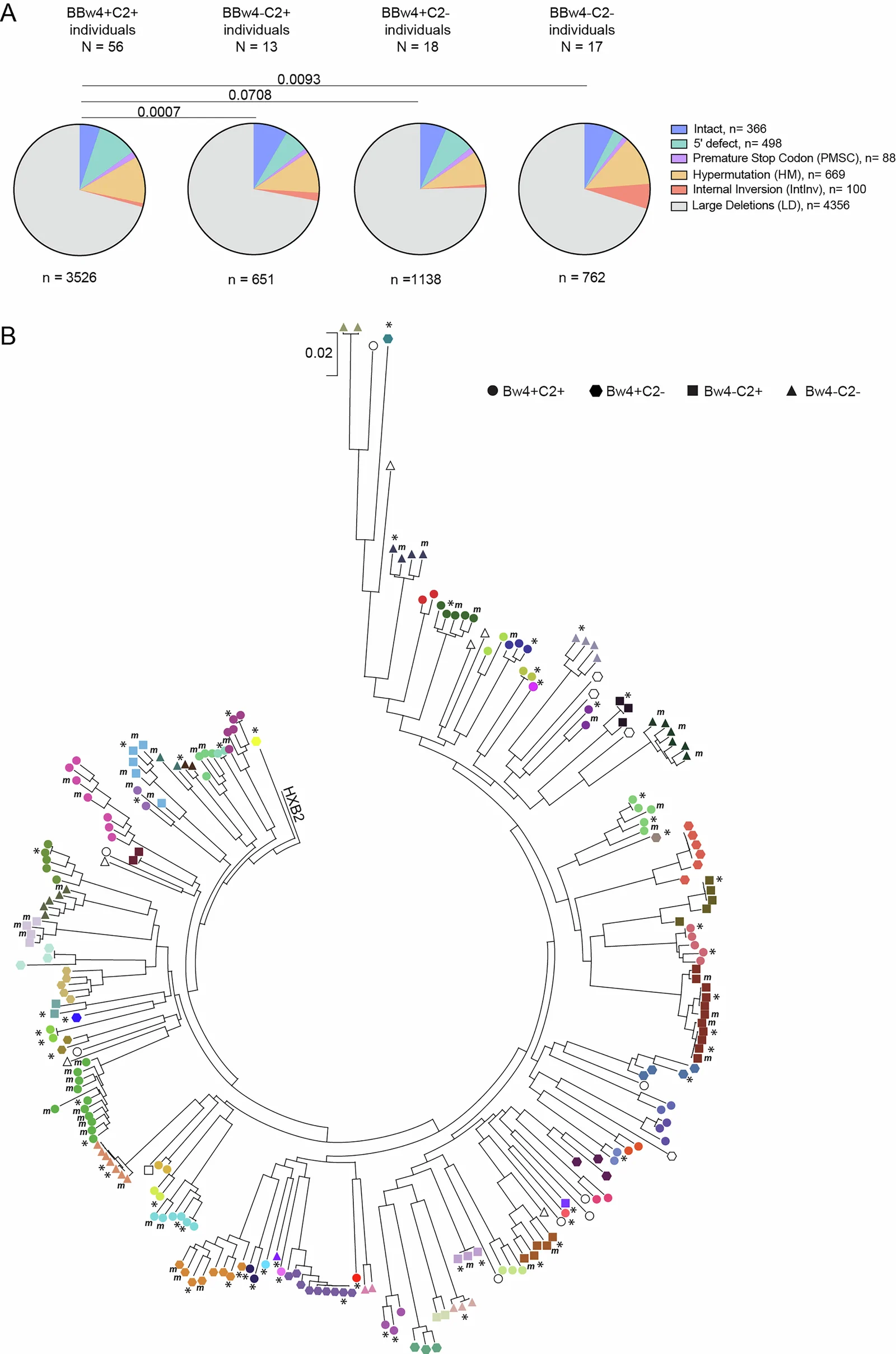
Proviral HIV-1 sequence landscape in study persons on long-term ART. **a**, Pie charts indicating the composition of HIV-1 proviral reservoir sequences in study persons on long-term ART with defined immunogenetic characteristics. Proportions of intact versus defective proviruses were compared using two-sided χ2 test. **b**, Circular maximum-likelihood phylogenetic tree of all intact proviruses identified in the study population of n = 104 individuals on long-term ART. All intact proviral sequences generated via the single-amplicon or the 5-amplicon approach (n = 245) derived from participants with detectable intact proviruses (n = 86) were included. HXB2, reference HIV-1 sequence. Clonal sequences are only shown once, with asterisk * indicating viral sequences that were detected in clones and symbol ^***m***^ indicating unique intact proviral sequences detected only using a five-amplicon approach. Sequences derived from a given study person are highlighted in the same color; immunogenetic characteristics of study persons are represented by symbols; open symbols indicate individuals in whom only one intact proviral sequence was detected. Source data

**Extended Data Fig. 2:**
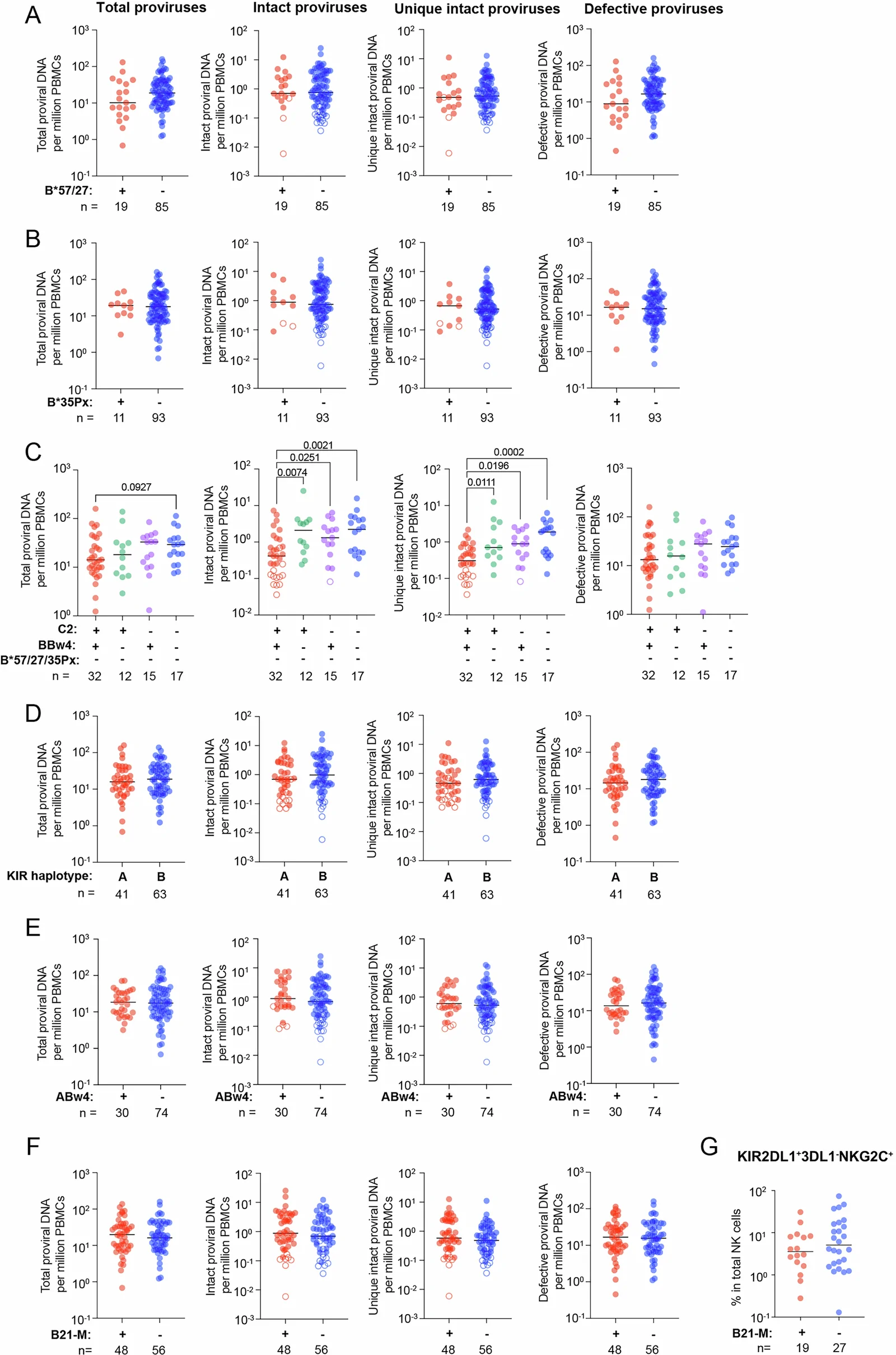
Immunogenetic determinants of HIV-1 reservoir size. **a-b**, Frequencies of indicated HIV-1 proviruses in carriers of HLA-B*27 or -B*57 (**a**) or -B35*Px (**b**), relative to individuals lacking the respective immunogenetic features. **c**, Frequencies of indicated HIV-1 proviruses in participants on long-term ART carrying both HLA-C2 and HLA-BBw4 alleles, in individuals lacking both alleles, and those with either HLA-C2 or HLA-BBw4 alone, following exclusion of individuals expression HLA-B*27 or -B*57 or -B*35Px. **d, e**, Frequencies of indicated HIV-1 proviruses in carriers of KIR-A or KIR-B haplotypes (**d**), and in carriers of HLA-ABw4 alleles versus non-carriers of HLA-ABw4 alleles (**e**). **f, g**, Frequencies of indicated HIV-1 proviruses (**f**) or KIR2DL1^+^ KIR3DL1^−^ NKG2C^+^ NK cells (**g**) in carriers of the HLA-B -21M/T signal peptide polymorphism. When members of clones of intact proviruses were only counted once, they are referred to as “unique intact proviruses”. n denotes the number of independent study participants. Open circles represent data at limit of detection. Horizontal lines reflect the median. Significance was tested using two-tailed Mann-Whitney *U* test for comparisons of two groups and Kruskal-Wallis with Dunn’s post-hoc test, nominal p values are shown. Source data

As a first step to investigate immune responses that can target HIV-1 reservoir cells, we conducted an immunogenetic association study to characterize linkages between HIV-1 reservoir sizes and sequence variations in the HLA class I locus, which critically influence host immune responses mediated by cytotoxic T and NK cells. For this purpose, we used single-genome, near-full-length proviral next-generation sequencing^28^ to characterize the frequencies of genome-intact and genome-defective proviruses in a cohort of *n* = 104 study participants who live with HIV-1 (PLWH) and had received ART for a median of 21 (range: 10–27) years at the time of peripheral blood mononuclear cell (PBMC) collection (Supplementary Table 1). In total, these experiments identified *n* = 6,077 HIV-1 DNA amplification products, of which *n* = 366 were defined as genome-intact, using a previously described computational code^7^ (Fig. 1a and Extended Data Fig. 1a,b). Approximately 38.25% (*n* = 140) of these genome-intact proviruses were identified only once in a given study participant, while 61.75% (*n* = 226) of proviruses were detected at least two times in individual study participants and represented members of proviral clones that result from the proliferative turnover of HIV-1-infected cells^29–32^. Annotating this dataset with corresponding high-resolution HLA class I typing data demonstrated that HLA-B*57 and HLA-B*27, which restrict immunodominant HIV-1-specific cytotoxic T cell responses and are associated with protection against HIV-1 disease progression during untreated infection^33^, were not associated with lower frequencies of intact HIV-1 proviruses during suppressive ART (Extended Data Fig. 2a). Moreover, HLA-B*35Px alleles, which are associated with accelerated HIV-1 disease progression during untreated infection^34^, showed no influence on HIV-1 reservoir size (Extended Data Fig. 2b). However, subsequent analysis demonstrated marked reductions of intact proviruses in carriers of HLA class I alleles influencing NK cell education. In particular, individuals with at least one HLA-C2 allotype, which promotes strong NK cell education through high-avidity interactions with KIR2DL1 (refs. ^35,36^), had significantly lower frequencies of intact proviruses compared to study participants carrying only HLA-C1 alleles (Fig. 1b); this was true when all intact proviruses were counted, when clonal intact proviral sequences were only counted once and when results were adjusted for race or for the presence of the protective (HLA-B*57 and HLA-B*27) or disease-accelerating (HLA-B*35Px) alleles (Table 1 and Extended Data Fig. 2c). A similar observation was made for carriers of at least one HLA-B Bw4 (BBw4) allotype (Fig. 1c), which can support NK cell education through interactions with KIR3DL1^37–39^; these individuals displayed significantly reduced frequencies of intact proviruses relative to study persons without BBw4 alleles, independently of HLA-B*57, HLA-B*27 and HLA-B*35Px expression or race (Table 1 and Extended Data Fig. 2c). Frequencies of defective proviruses, including viral genomes with defects at the 5’-LTR end, with statistically significant hypermutations, and with large deletions, also tended to be lower in carriers of at least one C2 or one BBw4 allele, but fold-differences relative to C2- or BBw4-negative persons were more limited (Fig. 1b,c and Table 1). Since the HLA-C2 and HLA-B Bw4 loci are in strong linkage disequilibrium (LD)^40^, we further classified our study persons based on the combined presence or absence of at least one C2 and one BBw4 allele. This analysis revealed the lowest measured proviral frequencies in carriers of both HLA-C2 and BBw4 alleles, while individuals who expressed neither C2 nor BBw4 had the highest frequencies of intact proviruses (Fig. 1d and Extended Data Fig. 2c); study persons expressing at least one C2 allele in the absence of BBw4 allele also tended to have lower reservoirs of intact proviruses, although the proportion of such individuals was more limited. Analysis of genetic variations within the KIR gene locus demonstrated almost ubiquitous expression of KIR2DL1 and KIR3DL1 across the entire study cohort (Supplementary Table 1). No differences in HIV-1 reservoir sizes were detected between carriers of KIR-A and KIR-B haplotypes (Extended Data Fig. 2d); moreover, the presence of HLA-A Bw4 allotypes (ABw4) was not associated with altered viral reservoir size, consistent with prior studies suggesting that HLA-A Bw4 alleles do not effectively contribute to NK cell education^41^ and represent functionally weaker ligands for KIR3DL1^42^ (Extended Data Fig. 2e). Together, these investigations strongly support the hypothesis that viral reservoir sizes during long-term ART are influenced and modulated by immunogenetic characteristics impacting NK cell education.

**Table 1:** Association between HLA-C2 or BBw4 and HIV-1 reservoir size determined by linear regression analysis

HIV-1 reservoir size analysis (*n* = 104 study participants)	Variable	Unadjusted		Adjusted based on race and HLA-B (*57, *27, *35Px) alleles		Adjusted based on race only	
		Effect	*P* value	Effect	*P* value	Effect	*P* value
Total proviruses	C2 presence	−0.171	0.072	−0.170	0.098	−0.188	0.056
Intact proviruses		−0.330	0.013	−0.401	0.0046	−0.382	0.005
Unique intact proviruses		−0.360	0.0017	−0.398	0.0014	−0.395	0.0009
Defective proviruses		−0.176	0.079	−0.162	0.133	−0.186	0.074
Total proviruses	BBw4 presence	−0.154	0.122	−0.118	0.275	−0.150	0.143
Intact proviruses		−0.335	0.010	−0.391	0.008	−0.351	0.01
Unique intact proviruses		−0.379	0.0016	−0.401	0.002	−0.390	0.002
Defective proviruses		−0.151	0.150	−0.106	0.348	−0.148	0.171

### No protective effects of HLA-C2 during untreated HIV-1 infection

Because associations between HLA class I alleles and frequencies of intact proviruses during long-term ART could reflect immune effects occurring before the initiation of therapy^33,43^, we examined relationships between the HLA-C2 and BBw4 alleles and plasma viral loads in PLWH not treated with ART. Among *n* = 4,007 ART-naive study participants recruited in a previous cohort study^44^, HLA-C2 was not associated with reduced plasma viremia, regardless of coexpression of the protective HLA class I alleles (B*57, B*27 and B*35Px) or race (Fig. 1e and Table 2). HLA-B Bw4 alleles correlated with lower viral loads; however, this effect was no longer detectable after adjusting for race and HLA-B*57, HLA-B*27 or HLA-B*35Px carrier status (Fig. 1e and Table 2). Combined presence or absence of HLA-C2 and HLA-B Bw4 was also unrelated to viral loads in untreated individuals after excluding carriers of HLA-B*27 and HLA-B*57 (Fig. 1e). By contrast, in our independent cohort of *n* = 104 individuals on long-term ART, the effects of HLA-C2 and HLA-B Bw4 on viral reservoir size remained detectable after adjusting for the presence of HLA-B*57, HLA-B*27 and HLA-B*35Px or race (Table 1 and Extended Data Fig. 2c). Together, these findings indicate that the associations between HLA-C2 and/or HLA-BBw4 and the frequencies of intact proviruses during long-term ART likely reflect immune pressure specifically acting on reservoir cells under suppressive therapy rather than effects established before ART initiation.

**Table 2:** Association between HLA-C2 or BBw4 and mean HIV-1 viral loads in untreated individuals, determined by linear regression analysis

HIV-1 viral load analysis (*n* = 4,007 study participants)	Unadjusted		Adjusted based on race and HLA-B (*57, *27, *35Px) alleles	
	Effect	*P* value	Effect	*P* value
C2 presence	−0.051	0.239	0.015	0.720
BBw4 presence	−0.286	3.6 × 10^−11^	−0.024	0.592

### KIR2DL1^+^KIR3DL1^−^NKG2C^+^ NK cells inversely correlate with HIV-1 reservoir size

**Fig. 2:**
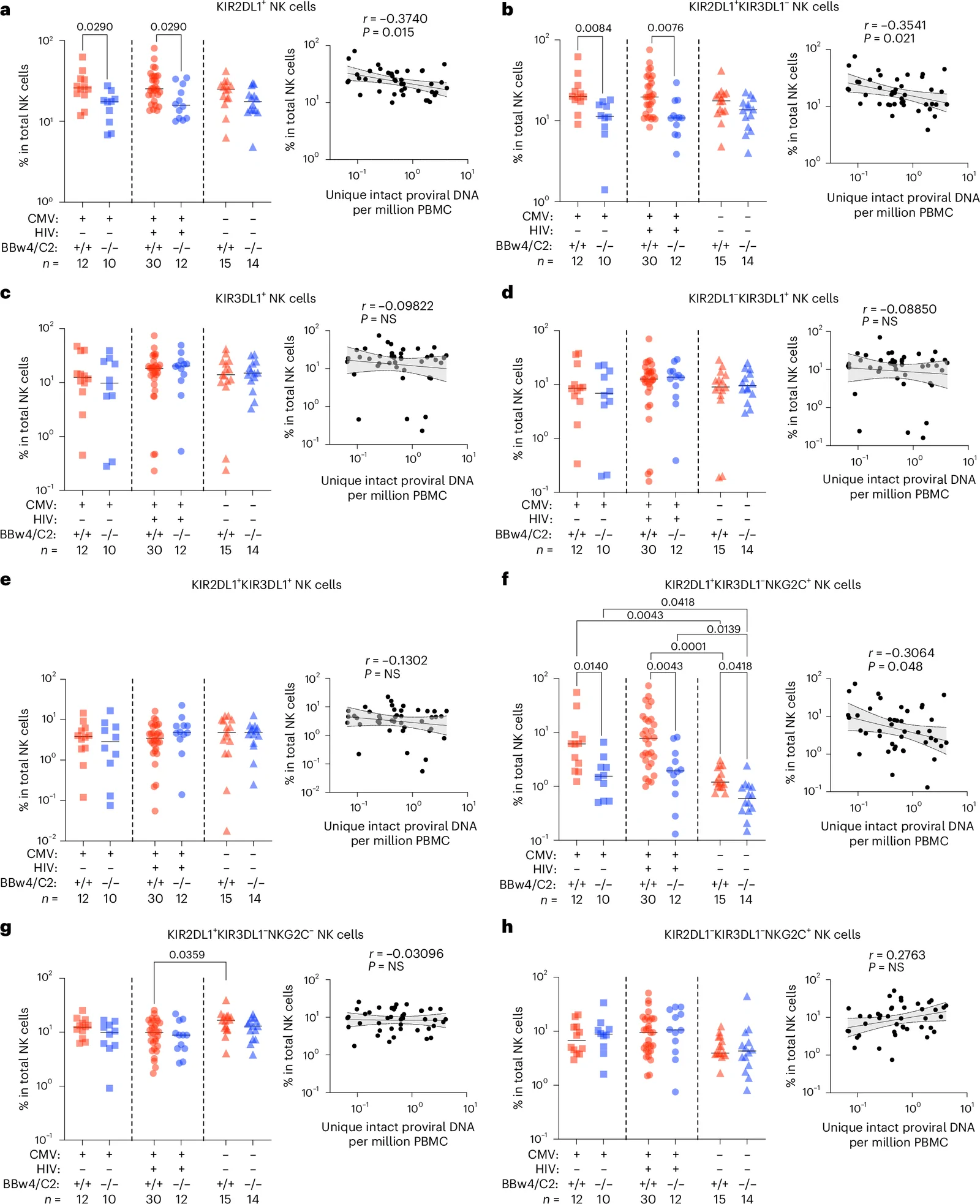
KIR2DL1^+^ NK cells are inversely associated with intact HIV-1 proviruses during long-term ART. **a–h**, Proportions of KIR2DL1^+^ (**a**), KIR2DL1^+^KIR3DL1^−^ (**b**), KIR3DL1^+^ (**c**), KIR2DL1^−^KIR3DL1^+^ (**d**), KIR2DL1^+^KIR3DL1^+^ (**e**), KIR2DL1^+^KIR3DL1^−^NKG2C^+^ (**f**), KIR2DL1^+^KIR3DL1^−^NKG2C^−^ (**g**) and KIR2DL1^−^KIR3DL1^−^NKG2C^+^ (**h**) NK cells in CMV^+^HIV^−^ individuals, ART-treated CMV^+^HIV^+^ individuals and CMV^−^HIV^−^ individuals carrying both HLA-C2 and BBw4 alleles or neither. Correlation plots demonstrate two-tailed Spearman correlations between frequencies of unique intact proviruses and the corresponding NK subsets. The regression line (solid) is plotted with its corresponding 95% confidence bands (shaded envelope). Horizontal lines reflect the median. Significance was tested using two-tailed Kruskal–Wallis with false-discovery rate (FDR)-corrected multiple comparison tests. NS, not significant. Source data

**Extended Data Fig. 3:**
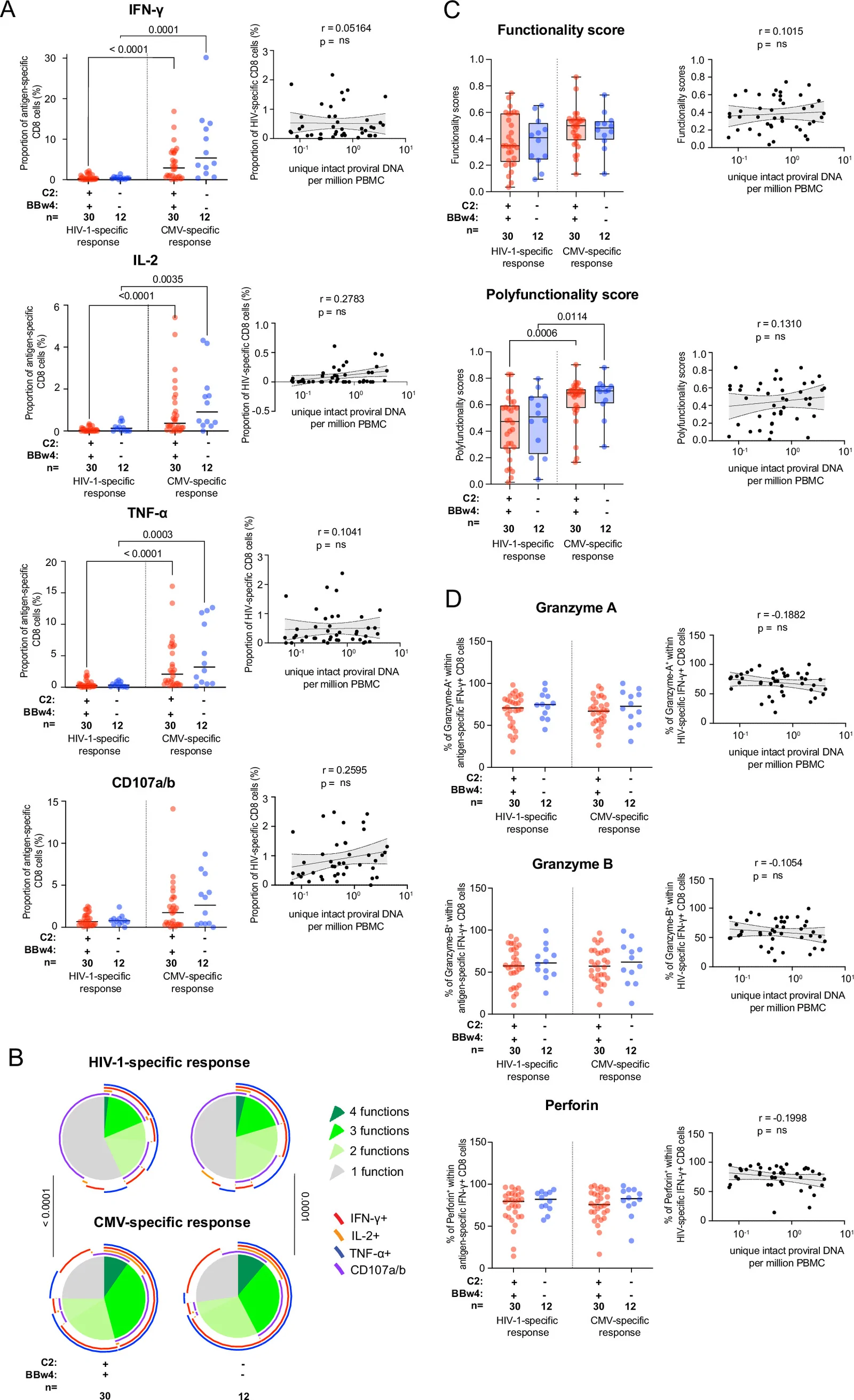
HIV-1-specific and CMV-specific CD8 T cell responses in study participants on long-term ART. **a**, Proportions of CD8 T cells secreting IFN-γ, IL-2, TNF-α and CD107a/b in response to stimulation with overlapping peptides spanning HIV-1 Gag or CMV pp65/IE-1. Data show background-corrected values after subtraction of cytokine secretion in unstimulated control. Correlation plots demonstrate two-tailed Spearman correlations between frequencies of unique intact proviruses and proportions of the respective cytokine-producing CD8 T cells. **b**, Polyfunctional CD8 T cell responses specific to HIV-1 and CMV antigens. Proportions of cells secreting one or multiple cytokines in response to antigen stimulation are highlighted by color-coding; individual effector functions are reflected by arches. Significance was tested using Permutation tests embedded in SPICE. **c**, Functionality (FS) and polyfunctionality (PFS) scores of HIV- and CMV- specific CD8 + T cells in CMV + HIV+ individuals carrying both HLA-C2 and -BBw4 alleles or lacking both alleles. Scores were computed using the COMPASS framework, which applies a Bayesian hierarchical model to intracellular cytokine staining data measuring four functions (IFN-γ, IL-2, TNF-α and CD107a/b). The model was fitted using 40,000 Markov Chain Monte Carlo (MCMC) iterations across 8 independent replications, with a fixed random seed for reproducibility. Each point represents one individual; boxes indicate median and interquartile range; whiskers extend to minimum and maximum values. **d**, Proportions of antigen-specific CD8 T cells expressing cytotoxic effector molecules Granzyme A, Granzyme B and Perforin. Correlation plots demonstrate two-tailed Spearman correlations between frequencies of unique intact proviruses and proportions of the respective cytotoxic molecules-producing HIV-1-specific CD8 T cells. n denotes the number of independent study participants. The regression line (solid) is plotted with its corresponding 95% confidence bands (shaded envelope). Horizontal lines reflect the medians. Significance was tested using two-tailed Kruskal-Wallis with false-discovery rate (FDR)-corrected multiple comparison tests (**a**, **c**-**d**). Source data

**Extended Data Fig. 4:**
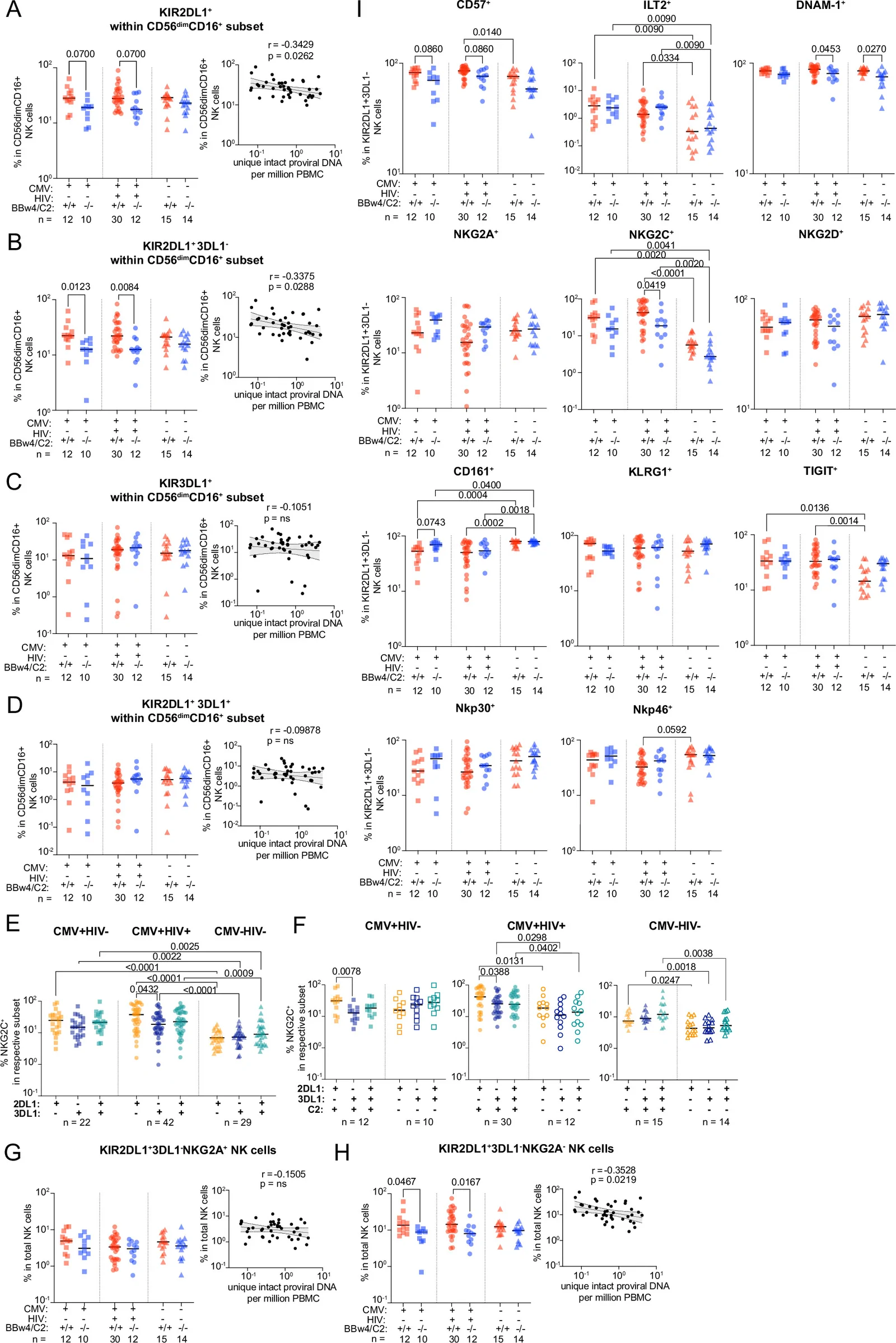
Phenotypic properties of KIR2DL1^+^ KIR3DL1^−^ NK cells in study persons on long-term ART. **a-d**, Proportions of KIR2DL1^+^ (**a**), KIR2DL1^+^ 3DL1^−^(**b**), KIR3DL1^+^ (**c**), KIR2DL1^+^ 3DL1^+^ (**d**) cytotoxic CD56^dim^CD16^+^ NK cells in CMV + HIV-, CMV + HIV+ and CMV- HIV- individuals carrying both HLA^−^C2 and -BBw4 alleles or lacking both alleles. Correlation plots demonstrate two-tailed Spearman correlations between frequencies of unique intact proviruses and respective NK subsets. Data are shown for CMV + HIV- study persons, CMV + HIV+ individuals on long-term ART, and HIV- CMV- study participants with indicated immunogenetic backgrounds. **e-f**, Proportions of NKG2C^+^ NK cells within KIR2DL1^+^ and/or KIR3DL1^+^ NK cell subpopulations from indicated study cohorts. Data are shown for all study participants in each cohort (**e**), or in study participants stratified based on HLA-C2 carrier status (**f**). **g, h**, Proportions of KIR2DL1^+^ KIR3DL1^−^ NKG2A^+^ (**g**) and KIR2DL1^+^ KIR3DL1^−^ NKG2A^−^ (**h**) NK cell subsets in CMV + HIV-, CMV + HIV + , and CMV- HIV- study cohorts with indicated immunogenetic characteristics. Correlation plots reflect two-tailed Spearman correlations between respective proportions of indicated NK cells and frequencies of unique intact HIV-1 proviruses. **i**, Data reflect expression of indicated surface markers on KIR2DL1^+^ KIR3DL1^−^ NK cells in CMV + HIV-, CMV + HIV+ and CMV- HIV- study persons stratified based on indicated immunogenetic characteristics. The regression line (solid) is plotted with its corresponding 95% confidence bands (shaded envelope). Horizontal lines reflect the median. Significance was tested using two-tailed FDR-controlled Mann-Whitney *U* test in panel **e-f**, and two-tailed Kruskal-Wallis with false-discovery rate (FDR)-corrected multiple comparison tests (**a**-**d**, **i**, **g**-**h**). Source data

To explore mechanisms underlying the immunogenetic associations between HLA-C2 and/or BBw4 alleles and lower frequencies of intact proviruses, we initially evaluated the magnitude of HIV-1 Gag-specific T cell responses in a subgroup of PLWH on ART who jointly carried at least one C2 allele and one BBw4 allele, or none of these two types of alleles (Supplementary Tables 2 and 3). However, using established assays to quantify the frequencies of HIV-1-specific CD8^+^ T cells expressing the cytokines IFN-γ, TNF or IL-2, or the degranulation marker CD107a/b, we observed that the proportions and polyfunctionality of Gag-specific CD8^+^ T cells were statistically unrelated to HLA-C2 or BBw4 carrier status, and not correlated with the frequencies of intact HIV-1 proviruses (Extended Data Fig. 3a–c). Moreover, HIV-1-specific CD8^+^ T cells that expressed perforin or granzyme A and B in response to Gag stimulation were not different between HLA-C2 and BBw4 co-carriers and individuals lacking such alleles (Extended Data Fig. 3d). CD8^+^ T cell responses recognizing CMV pp65 and IE-1, analyzed for control purposes, also did not vary significantly between individuals with or without C2 and BBw4 alleles (Extended Data Fig. 3a–d). As a subsequent step, we conducted a deep phenotypic characterization of NK cells in these study persons using a multiparametric spectral flow cytometry analysis involving 22 NK cell surface markers (Supplementary Table 4). These studies demonstrated that, relative to C2 and BBw4 double-negative study persons, C2 and BBw4 double-positive individuals on ART had significantly higher levels of NK cells expressing the HLA-C2 receptor KIR2DL1; moreover, proportions of these KIR2DL1^+^ NK cells, when assessed either within the total NK pool or within the highly cytotoxic CD56^dim^CD16^+^ NK population, were inversely correlated with the frequencies of intact proviruses (Fig. 2a and Extended Data Fig. 4a). A closer examination revealed that the link between KIR2DL1^+^ NK cells and reservoir sizes was confined to the KIR2DL1^+^KIR3DL1^−^ subset, which exhibited an inverse correlation with frequencies of intact proviruses (Fig. 2b and Extended Data Fig. 4b). In contrast, the proportions of total KIR3DL1^+^, KIR2DL1^−^KIR3DL1^+^ or KIR2DL1^+^KIR3DL1^+^ NK cells were not different between C2 and BBw4 co-carriers and noncarriers, and did not correlate with frequencies of intact proviruses; this was true when assessed within total NK cells or within the cytotoxic CD56^dim^CD16^+^ NK cell subset (Fig. 2c–e and Extended Data Fig. 4c,d). Collectively, these data indicate that KIR2DL1^+^ KIR3DL1-educated NK cells are selectively expanded in PLWH expressing HLA-C2 and inversely associated with the intact proviral reservoir sizes during long-term ART, suggesting a functional role for these cells in restricting the pool sizes of reservoir cells carrying intact proviruses.

**Extended Data Fig. 5:**
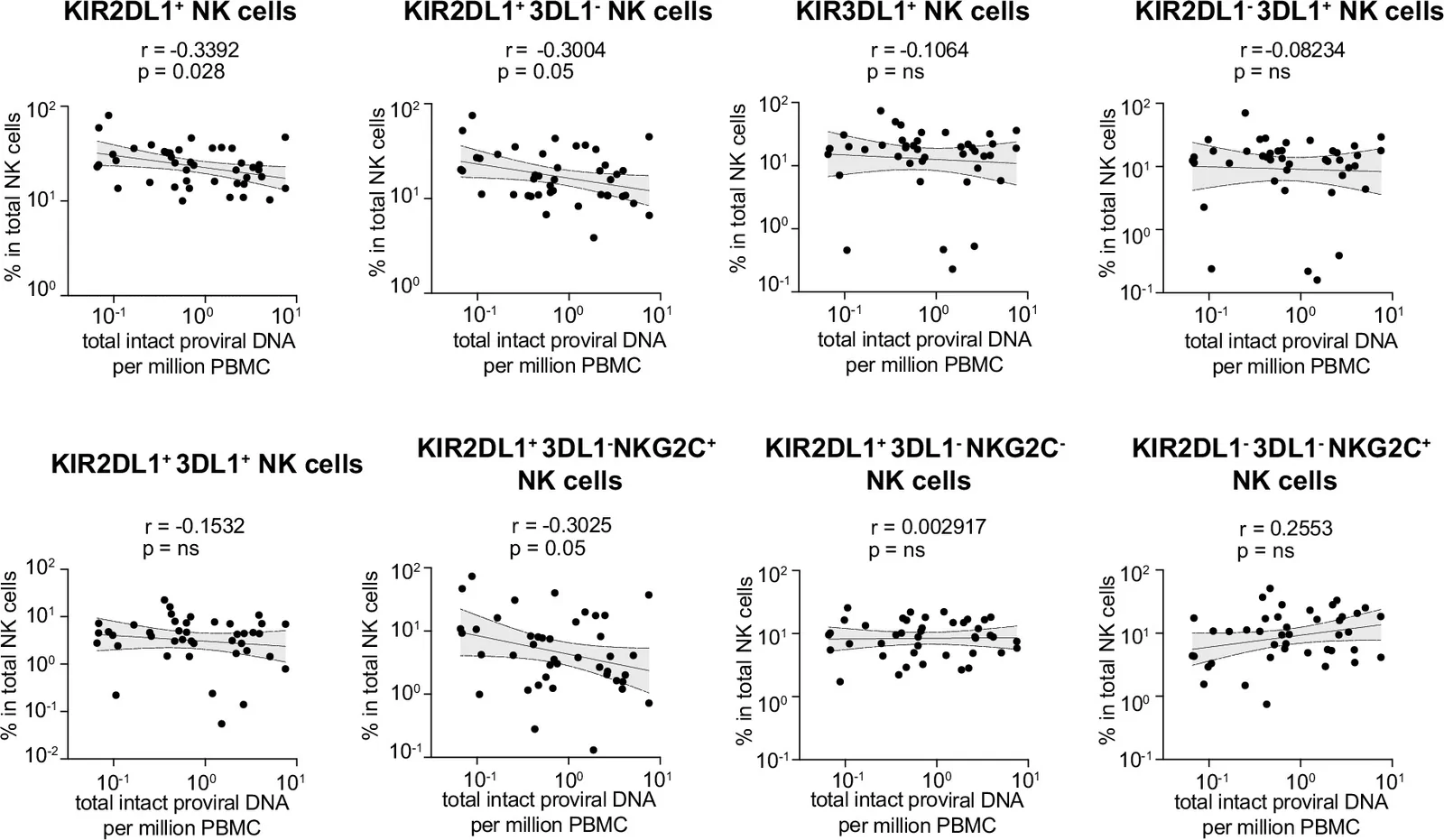
KIR2DL1^+^ NK cells are inversely associated with total intact HIV-1 proviruses during long-term ART. Correlation plots demonstrate two-tailed Spearman correlations between frequencies of total intact proviruses and the proportions of indicated NK subpopulations in ART-treated CMV + HIV+ individuals carrying both HLA-C2 and -BBw4 alleles or lacking both alleles. The regression line (solid) is plotted with its corresponding 95% confidence bands (shaded envelope). Source data

In a subsequent phenotypic characterization of KIR2DL1^+^KIR3DL1^−^ NK cells, we observed that these cells frequently coexpress the activating HLA-E receptor CD94/NKG2C (termed ‘NKG2C’ in this paper)^45^ (Extended Data Fig. 4e,f), a marker that denotes a subset of ‘adaptive’ NK cells^46,47^. In particular, we noted that KIR2DL1^+^KIR3DL1^−^NKG2C^+^ NK cells, a cell population reminiscent of single KIR^+^ adaptive NK cells previously described in oncologic contexts^48,49^, were selectively expanded in HLA-C2 carriers and inversely associated with the frequencies of intact proviruses (Fig. 2f). By contrast, proportions of NK cells expressing KIR2DL1 in the absence of NKG2C, or NKG2C in the absence of KIR2DL1, were not correlated with frequencies of intact proviruses and not quantitatively different between individuals with or without HLA-C2 (Fig. 2g,h). Interestingly, KIR2DL1^+^KIR3DL1^−^ NK cells also frequently coexpressed DNAM-1, CD57 and KLRG1, while exhibiting limited surface expression of the inhibitory HLA-E receptor NKG2A (Extended Data Fig. 4i), consistent with some of the previously recognized phenotypic features of adaptive NK cells^50^. Moreover, KIR2DL1^+^ NK cells coexpressing NKG2A did not differ between HLA-C2 carriers and noncarriers, while KIR2DL1^+^KIR3DL1^−^NKG2A^−^ NK cells, a cell population enriched for KIR2DL1^+^KIR3DL1^−^NKG2C^+^ cells, were expanded in HLA-C2 carriers and correlated inversely with the frequency of intact proviruses (Extended Data Fig. 4g,h). Notably, inverse correlations involving KIR2DL1^+^KIR3DL1^−^NKG2C^+^ NK cells were observed when clonal intact proviruses were counted only once (Fig. 2a–h) or when scored individually (Extended Data Fig. 5). In sum, these observations identify KIR2DL1^+^KIR3DL1^−^NKG2C^+^ NK cells as the subset with the most definitive inverse association with intact proviral reservoir size, suggesting that both KIR2DL1–HLA-C2-mediated NK cell education and NKG2C–HLA-E-driven NK cell activation contribute to shaping the innate cellular immune responses against persisting HIV-1 reservoir cells.

Since NKG2C^+^ adaptive NK cells are frequently expanded in response to CMV infection^51–53^, we first confirmed that over 98% (*n* = 102) of the participants in our original cohort of PLWH receiving ART were seropositive for CMV infection (Supplementary Table 1), as expected based on prior investigations^54^. Moreover, we expanded our phenotypic analysis to reference cohorts of HIV-negative study persons with or without CMV co-infection (Supplementary Tables 2 and 3); these additional studies demonstrated that the proportion of KIR2DL1^+^KIR3DL1^−^NKG2C^+^ NK cells in HLA-C2-expressing, HIV-negative, CMV-positive individuals was significantly elevated compared to their C2-negative counterparts, thus mirroring observations in the HIV^+^CMV^+^ study cohort (Fig. 2f). In contrast, the proportion of KIR2DL1^+^KIR3DL1^−^NKG2C^+^ NK cells was generally more limited in individuals who were seronegative for HIV and CMV, and differed less profoundly between HLA-C2-positive or C2-negative carriers from that cohort (Fig. 2f and Extended Data Fig. 4e,f). Together, these data suggest that CMV co-infection contributes to driving the expansion of KIR2DL1^+^NKG2C^+^ NK cells in PLWH; however, CMV infection by itself did not seem to represent a sufficient factor, as the degree of KIR2DL1^+^ NK cell expansion varied widely among CMV-positive study persons, both in the presence or absence of HIV-1 co-infection. This suggests that viral or host factors other than CMV infection can also modulate KIR2DL1^+^NKG2C^+^ NK cell expansion in PLWH on ART.

### Clonal expansion of adaptive NK cells

**Fig. 3:**
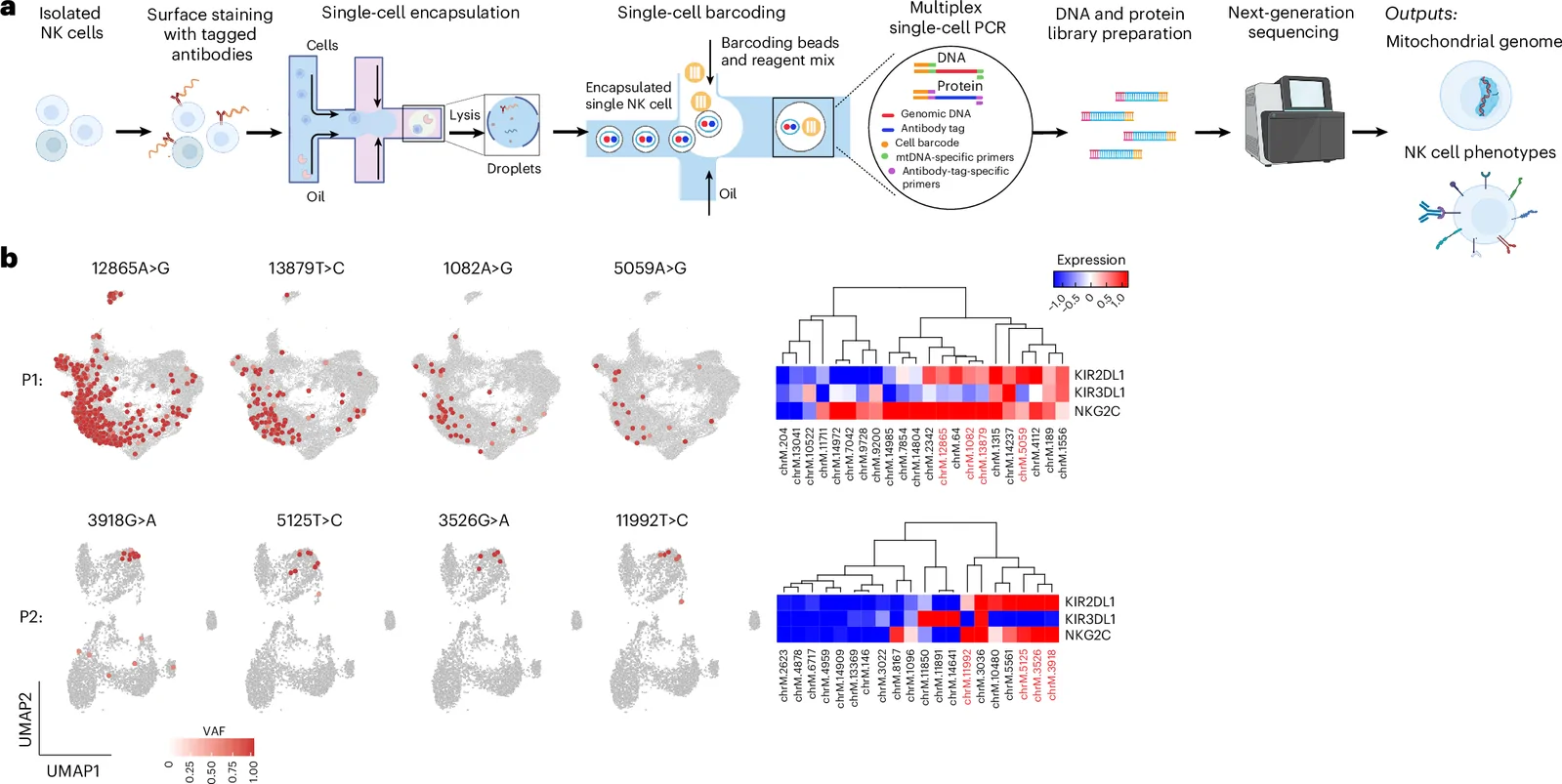
Clonal expansion of KIR2DL1^+^KIR3DL1^−^NKG2C^+^ NK cells. **a**, Schematic diagram depicting the workflow for the single-cell proteogenomic profiling assay to characterize genetic variations in mitochondrial DNA in phenotypically complex NK cell populations. **b**, Global visualization of the phenotypic profile of NK cells by two-dimensional UMAP plots in two study persons (P1, P2). Cells sharing specific mitochondrial sequence variations, used as internal barcodes to infer a common clonal origin, are highlighted. Heatmaps indicate phenotypic expression of KIR2DL1, KIR3DL1 and NKG2C in NK cell subsets sharing indicated mitochondrial DNA variants. VAF, variant allele frequency; chrM., mitochondrial chromosomal DNA. Panel **a** created using graphical elements from BioRender; Tan, T. https://biorender.com/rsssfyf (2026).

For a more detailed analysis of the underlying dynamics of KIR2DL1^+^KIR3DL1^−^NKG2C^+^ NK cells in study persons on ART, we searched for evidence of clonal proliferation within this cell pool, using a single-cell proteogenomic profiling assay to jointly assess mitochondrial sequence variations and the phenotypic properties of single, participant-derived NK cells^55,56^. By exploiting the higher mutation rate of mitochondrial DNA (mtDNA) relative to nuclear DNA^57^, this method tracks somatic mtDNA mutations that function as natural, endogenous barcodes^58,59^ and are inherited by the clonal descendants of NK cells that often share coordinated phenotypic traits. For this analysis, total NK cells isolated from HLA-C2-expressing study participants on ART were stained with oligonucleotide-tagged antibodies and then encapsulated as single cells within a microfluidics device designed to enable multiplex polymerase chain reaction (PCR) amplifications of nearly the entire 16 kb mitochondrial genome, and of antibody-derived oligonucleotide tags corresponding to surface markers (Fig. 3a). Using this approach, we identified multiple larger and smaller clones of KIR2DL1^+^KIR3DL1^−^NKG2C^+^ NK cells (Fig. 3b), each defined by distinct mtDNA sequence variations, in two study participants (P1, P2) subjected to this analysis, with stronger evidence for NK cell clonal proliferation in the individual (P1) with a lower reservoir (0.0882 versus 0.708 genome-intact proviruses per million PBMCs, respectively) and a larger population of KIR2DL1^+^KIR3DL1^−^NKG2C^+^ NK cells (73.6% versus 39.9% of total NK cells, respectively) compared to the other (P2) (Fig. 3b). These results imply that expansion of KIR2DL1^+^KIR3DL1^−^NKG2C^+^ NK cells in HLA-C2-expressing individuals on ART is at least in part driven by clonal proliferation; however, the extent of clonal NK cell proliferation within this specific NK cell subset varied considerably among study participants, suggesting that specific host- or virus-derived activating signals may regulate this process.

### HLA-E-restricted HIV-1 epitope variants and viral reservoir size

**Fig. 4:**
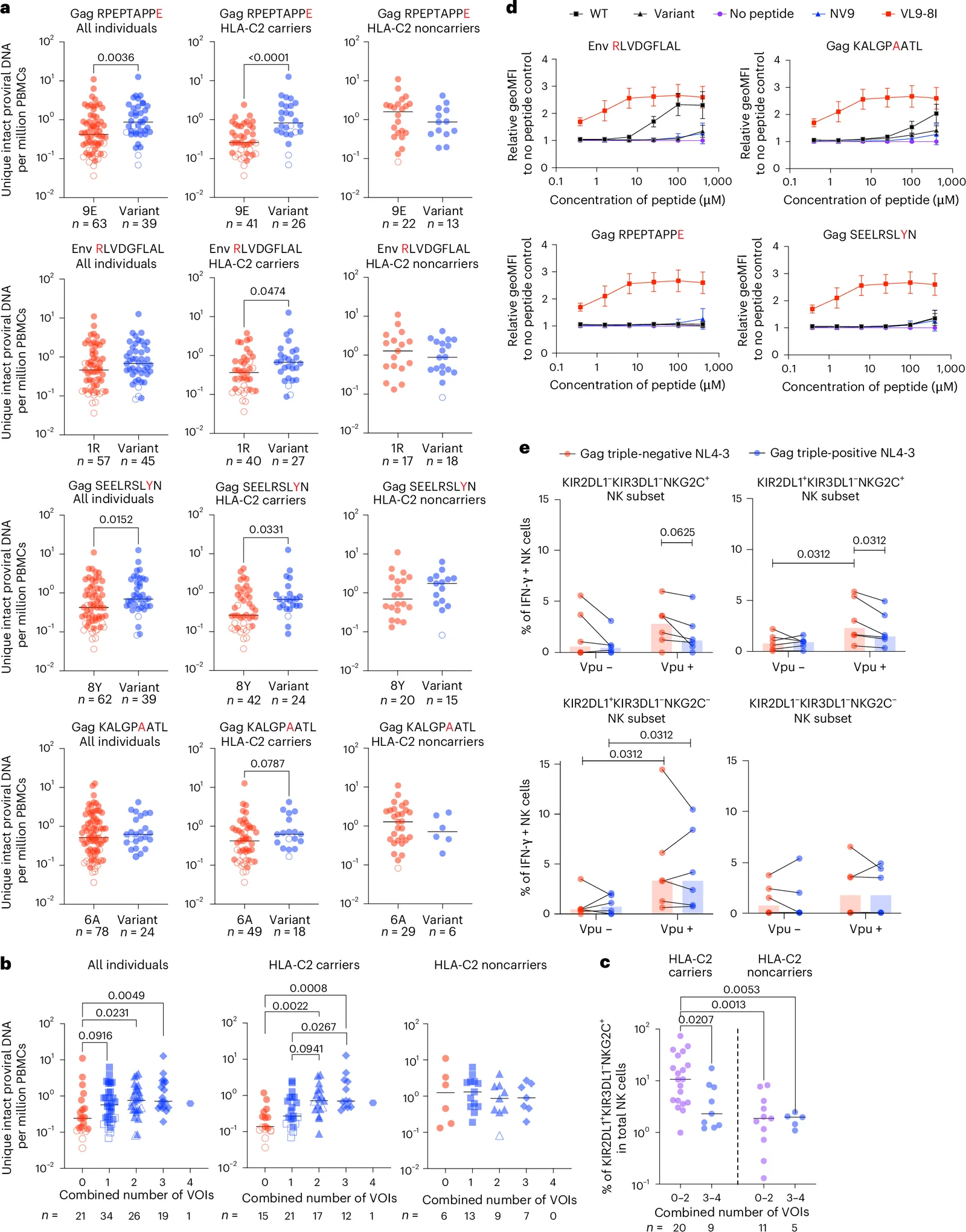
Sequence variations in HLA-E-restricted HIV-1 epitopes are associated with HIV-1 reservoir cell frequencies. **a**, Frequencies of unique intact HIV-1 proviruses in study participants on long-term ART who harbored the wild-type or respective variant sequence in the indicated HLA-E-restricted HIV-1 epitopes. Data are shown for all study persons on long-term ART (left), for all carriers of at least one HLA-C2 allele (middle) and for carriers of no HLA-C2 alleles (right). Two participants from the original cohort of *n* = 104 were excluded due to absence of detectable near-full-length proviruses. One additional participant was excluded from the SN9 epitope analysis due to autologous sequences harboring a small deletion within this epitope. **b**, Cumulative analysis of the frequencies of unique intact HIV-1 proviruses in individuals harboring 0, 1, 2, 3 or 4 of the respective HLA-E epitope variants. Data are shown for all study persons and for HLA-C2^+^ and HLA-C2^−^ individuals. Open circles represent data at the limit of detection. VOIs, variants of interest described in **a**. **c**, Proportions of KIR2DL1^+^KIR3DL1^−^NKG2C^+^ NK cells in individuals harboring 0–2 of the described HLA-E epitope variants, or containing 3–4 of the described HLA-E variants in autologous proviral sequences. Data are shown separately for carriers with or without HLA-C2. Open circles represent data at the limit of detection. Horizontal lines reflect the median. Significance was tested using two-tailed Mann–Whitney *U* tests for comparisons of two groups (**a**) and Kruskal–Wallis with FDR-corrected multiple comparison tests (**b**,**c**). **d**, HLA-E surface stabilization by HIV-1 epitopic peptides and peptide variants. HLA-E expression was determined by flow cytometry on HLA-E-transfected K562 cells after pulsing with serial dilutions of indicated wild-type or variant peptides described in **a**. The VL9-8I peptide was used as a positive control; the NV9 peptide was used a negative control. Data show the geometric mean fluorescence intensity (geoMFI) of BV421-conjugated HLA-E antibody relative to no peptide controls. The mean and standard deviation of three different experiments are shown. **e**, Interferon-γ secretion in indicated NK cell subsets after stimulation with autologous CD4^+^ T cells infected with viral NL4-3 constructs containing Vpu sequences that were able (Vpu +) or unable (Vpu −) to downregulate HLA-C and further engineered to harbor wild-type or variant sequences in the HLA-E-restricted Gag epitopes described in **a**–**c** (Y79F variant in the SN9 epitope, A340G variant in the KL9 epitope and E460A variant in the RE9 epitope). Results show background-corrected data after subtraction of IFN-γ secretion in NK cells exposed to uninfected control CD4^+^ T cells; bars indicate the median. Data from *n* = 6 HLA-C2^+^CMV^+^ healthy donors are shown. Significance was tested using a two-tailed Wilcoxon matched-pairs signed rank test. Source data

**Extended Data Fig. 6:**
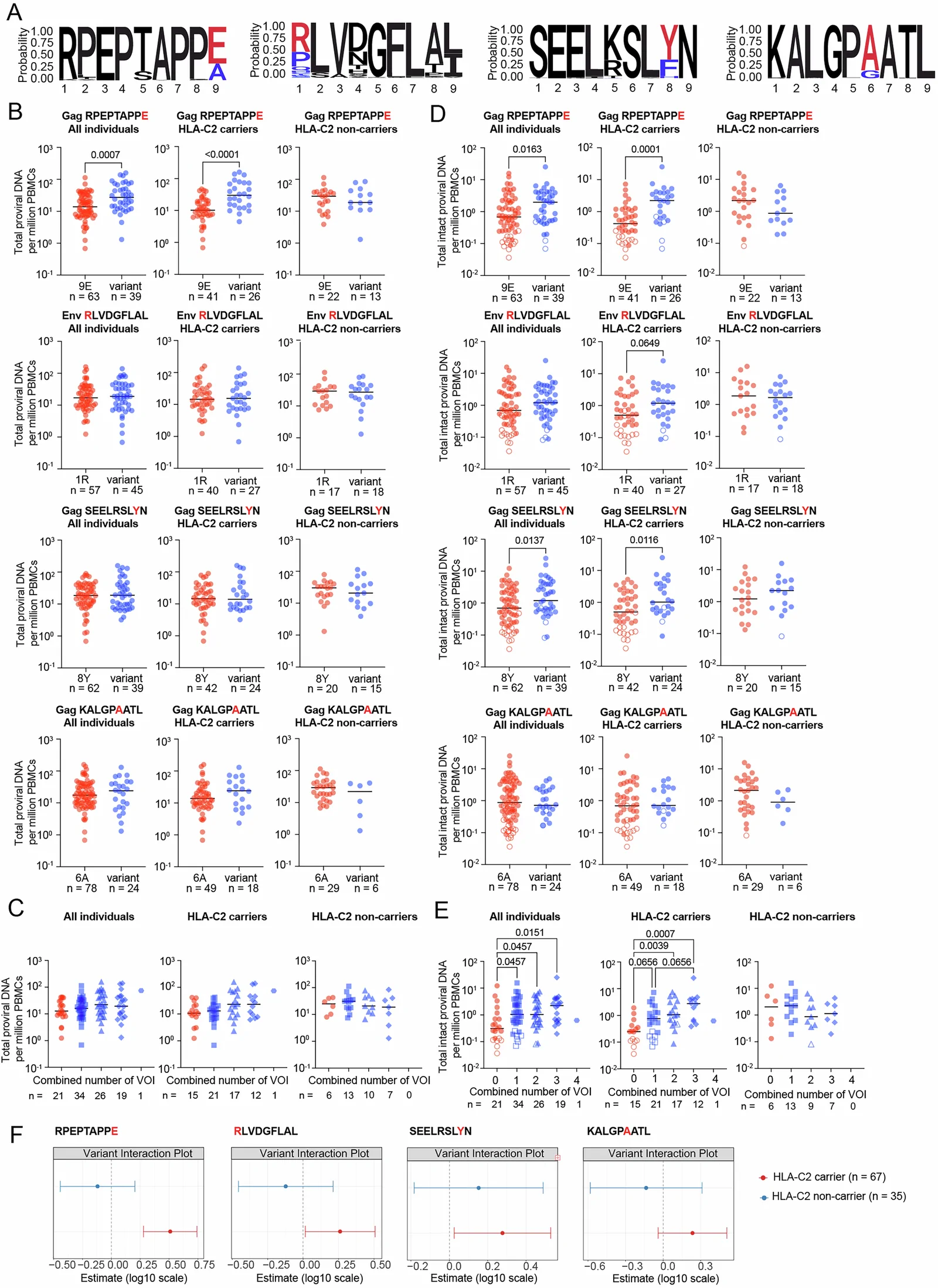
Influence of sequence variations in HLA-E epitopes on HIV-1 reservoir size. **a**, Probability-weighted sequence logos of four 9-mer HLA-E restricted HIV-1 epitopes based on a multiple sequence alignment of all peptide sequences from near-full length proviruses available from n = 102 study persons on long-term ART. Two participants from the original cohort of n = 104 were excluded due to lack of detectable near-full length proviruses. Amino acid residues highlighted in red box are naturally-occurring variants of interest. **b, d** Frequencies of total (**b**) or total intact (**d**) HIV-1 proviruses in study persons on long-term ART who harbored the wild-type or the respective variant sequence in the indicated HLA-E restricted HIV-1 epitopes. **c, e**, Cumulative analysis of the frequencies of total (**c**) or total intact (**e**) HIV-1 proviruses in individuals harboring 0, 1, 2, 3 or 4 of the respective HLA-E epitope variants. Data are shown for all study persons on long-term ART (left panel), for all carriers of at least one HLA-C2 allele (middle panel) and for carriers of no HLA-C2 alleles (right panel). Horizontal lines reflect the median. Significance was tested using two-tailed Mann-Whitney *U* test for comparisons of two groups and Kruskal-Wallis with false-discovery rate (FDR)-corrected multiple comparison tests. **f**, Forest plots showing the effect of each HLA-E–restricted HIV-1 epitope variant on log10-transformed unique intact provirus frequencies (related to Fig. 4a), estimated separately within HLA-C2 carriers and non-carriers. For each variant, a linear regression model was fit with an interaction term between HLA-C2 carrier status and epitope variant. Stratum-specific effects (variant versus wild-type) were derived from the model coefficients using the delta method. The centre of each error bar denotes the point estimate (fitted regression coefficient) of the stratum-specific effect, and error bars represent 95% confidence intervals. Source data

**Extended Data Fig. 7:**
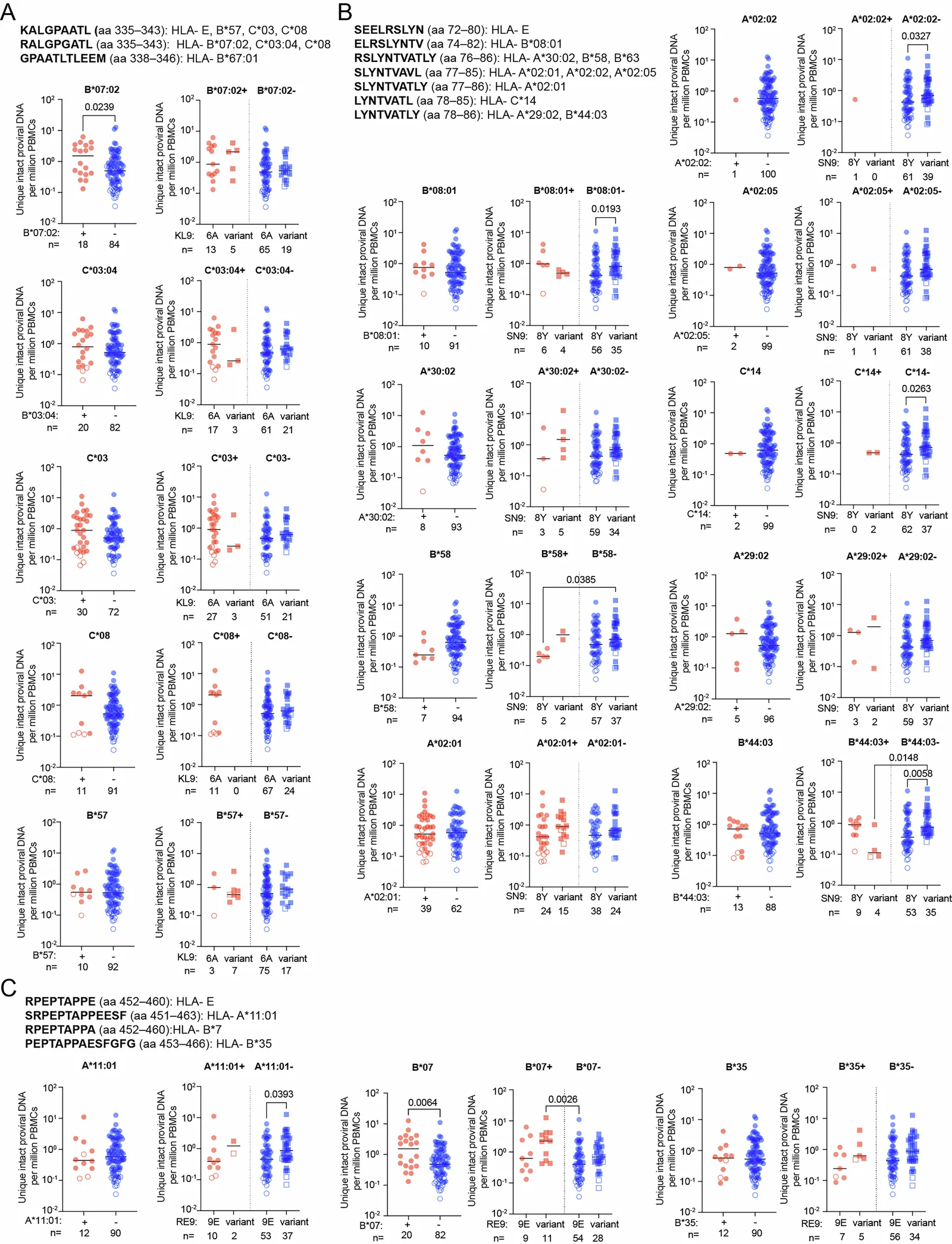
Sequence variations in CTL epitopes restricted by classical HLA class I alleles are not associated with HIV-1 reservoir size. **a-c**, Frequencies of unique intact proviruses in carriers of HLA class I alleles restricting the indicated optimal clade B CTL epitopes overlapping with the HLA-E-restricted KL9 (**a**), SN9 (**b**) and RE9 (**c**) epitopes, relative to non-carriers of the respective HLA alleles. CTL epitopes were identified in the Los Alamos Database (https://www.hiv.lanl.gov/content/immunology/tables/optimal_ctl_summary.html). No optimal clade B CTL epitopes restricted by classical HLA class I alleles were found to overlap with the HLA-E-restricted RL9 epitope. Carriers and non-carriers of these classical HLA class I alleles were further segregated based on the presence or absence of defined sequence variants in the indicated overlapping HLA-E-restricted epitopes. When members of clones of intact proviruses were only counted once, they are referred to as “unique intact proviruses”. n denotes the number of independent study participants. Open circles represent data at limit of detection. Horizontal lines reflect the median. Significance was tested using two-tailed Mann-Whitney *U* test (left panels) and Kruskal-Wallis with false-discovery rate (FDR)-corrected multiple comparison tests (right panels). Source data

We explored mechanisms underlying the clonal expansion of KIR2DL1^+^NKG2C^+^ NK cells by focusing on sequence polymorphisms in viral and host peptides that can modulate HLA-E surface stability. The HLA-B21 M/T signal peptide polymorphism, previously shown to influence HLA-E surface stabilization^60^, was not associated with the proportion of KIR2DL1^+^KIR3DL1^−^NKG2C^+^ NK cells nor with the frequency of intact HIV-1 proviruses in our cohort of PLWH on long-term ART (Extended Data Fig. 2f,g). Since NKG2C–HLA-E interactions can influence NK cell responses against CMV through variant-specific recognition of the canonical CMV UL40-encoded VL9 peptide^56,61–64^, we next investigated whether sequence variations in HIV-1-derived, noncanonical HLA-E-restricted epitopes can impact HIV-1 reservoir size through peptide- or peptide-variant-specific NKG2C engagement during prolonged ART. To address this, we analyzed the amino acid diversity in previously characterized HLA-E-restricted HIV-1 epitopes^47,65,66^ within autologous near-full-length proviral sequences from our original cohort of study participants on ART (Fig. 4a and Extended Data Fig. 6a,b,d). We observed that the E460A Gag variant, located at the HLA-E anchor position 9 of the peptide RPEPTAPPE, was strongly associated with higher frequencies of intact proviruses in HLA-C2 carriers, but not in HLA-C2-negative individuals; moreover, a similar observation was made for the R747P variant, located at position 1 in the HLA-E restricted Env epitope RLVDGFLAL. In addition, for two other previously characterized HLA-E epitopes in Gag, we noted higher reservoirs associated with amino acid variations at the NKG2C contact residues^67^ at position 6 (A340G in KALGPAATL) and position 8 (Y79F in SEELRSLYN), although these differences did not reach statistical significance for the KALGPAATL epitope. These associations were preferentially observed in HLA-C2 carriers, consistent with our hypothesis that NKG2C–HLA-E-dependent activation signals converge with KIRDL1–HLA-C2-mediated NK cell education in shaping the innate cellular immune responses against HIV-1 reservoir cells. These findings were further corroborated by a linear regression model incorporating multiplicative interaction terms, which revealed statistically significant interactions between HLA-C2 and the E460A, R747P and Y79F variants. These results confirmed a synergistic effect, demonstrating that these specific variants in HLA-E-restricted epitopes modify the protective association of the HLA-C2 allele (Extended Data Fig. 6f). Moreover, cumulative analyses demonstrated a dose-dependent relationship, with greater numbers of these HLA-E epitope variants corresponding to progressively higher frequencies of intact proviruses (Fig. 4b and Extended Data Fig. 6c,e) and lower proportions of KIR2DL1^+^NKG2C^+^ NK cells (Fig. 4c) in carriers of HLA-C2, but not in individuals lacking C2 alleles. Because some of these HLA-E-restricted HIV-1 epitopes overlap with viral cytotoxic T cell epitopes restricted by HLA-A, HLA-B or HLA-C alleles, we considered the possibility that associations between the described peptide variants and viral reservoir size reflected HIV-1-specific CD8^+^ T cell effects on HIV-1 reservoir cells. However, a detailed analysis of viral sequence variations in carriers of defined HLA class I alleles found no evidence supporting the hypothesis that HIV-1-specific CD8^+^ T cells restricted by classical HLA class I alleles reduced HIV-1 reservoir cell frequencies (Extended Data Fig. 7a–c). Together, these findings suggest that, in the context of KIR2DL1–HLA-C2 NK cell education, viral sequence diversity within HLA-E-restricted HIV-1 epitopes shapes NKG2C-dependent NK cell activation and, through this mechanism, influences the size of the genome-intact HIV-1 reservoir.

**Extended Data Fig. 8:**
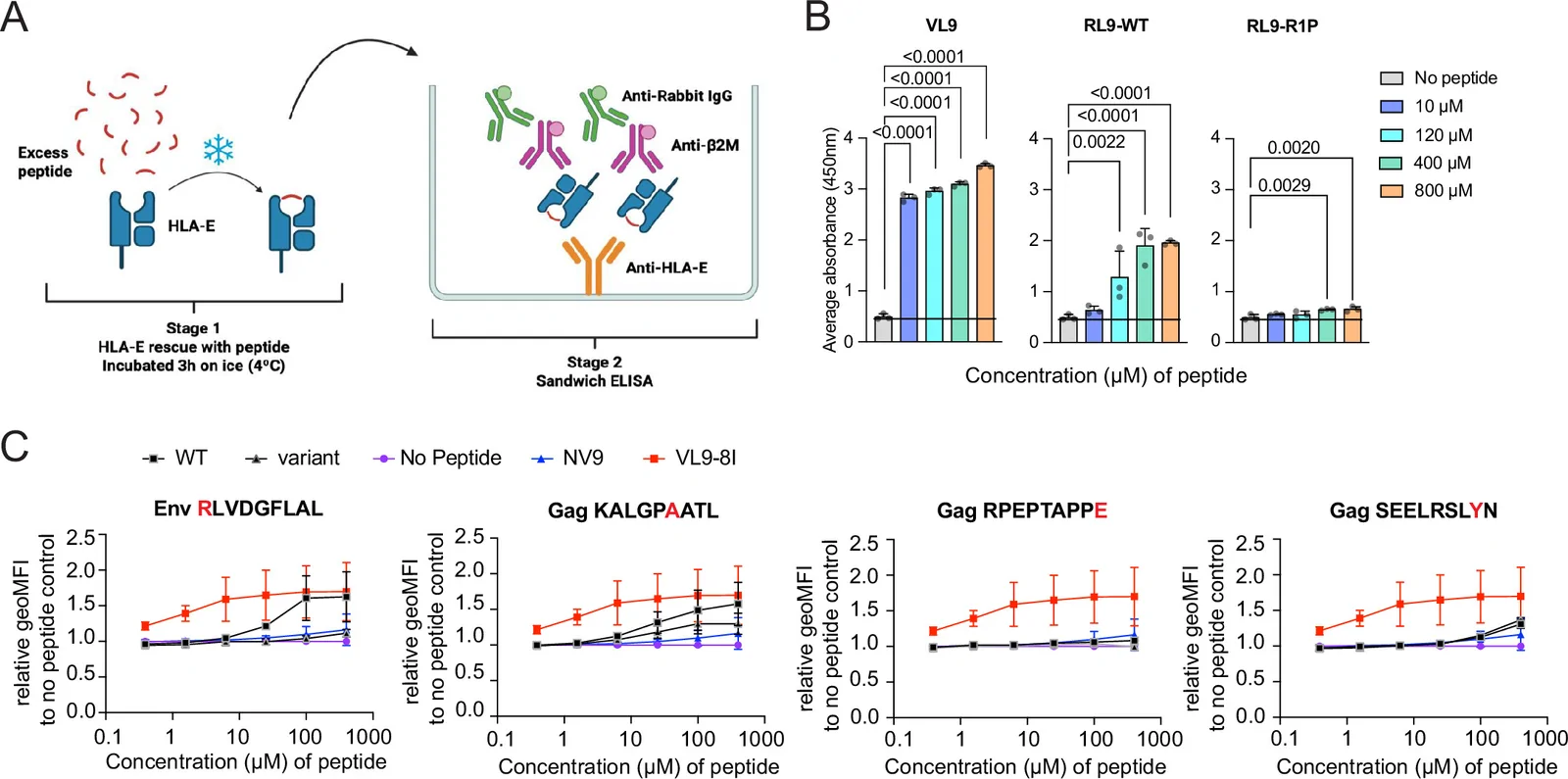
HLA-E binding of wild-type and variant-form HIV-1 peptides. **a**, Schematic sketch of the ELISA-based peptide-binding assay in which peptide-free HLA-E–β2M complexes are incubated with excess test peptide (red). The relative concentration of β2M-associated HLA-E complexes is measured indirectly by sandwich ELISA using anti-HLA-E capture and anti-β2M detection (violet) antibodies, followed by antibody-mediated signal amplification (green), enabling selective detection of peptide-rescued HLA-E-β2M complexes. **b**, Average absorbance at 450 nm is shown as the peptide concentration increases from 0 to 800 μM. Positive control VL9 (VMAPRTLVL), the wild-type Env RL9 epitope, and its variant (R1P) peptides are assayed. The gray bar represents the no-peptide background control. Three biological replicates (n = 3), each consisting of three individual peptide-binding reactions per test peptide, were performed simultaneously, with two technical replicates per biological replicate (technical replicates=2). Bars show means and standard deviations. Significance was tested using an Ordinary One-Way ANOVA with false-discovery rate (FDR)-corrected multiple-comparison tests. **c**, HLA-E surface stabilization by HIV-1 epitopic peptides and peptide variants. HLA-E expression was determined by flow cytometry on HLA-E-transfected TAP-deficient murine RMA-S cells after pulsing with serial dilutions of indicated wild-type or variant peptides described in the manuscript text. The VL9-8I peptide was used as a positive control, while the NV9 peptide was used a negative control. Data show the geometric mean fluorescence intensity (geoMFI) of BV421-conjugated HLA-E antibody relative to no peptide controls. The mean and standard deviation of three different experiments are shown. Source data

**Extended Data Fig. 9:**
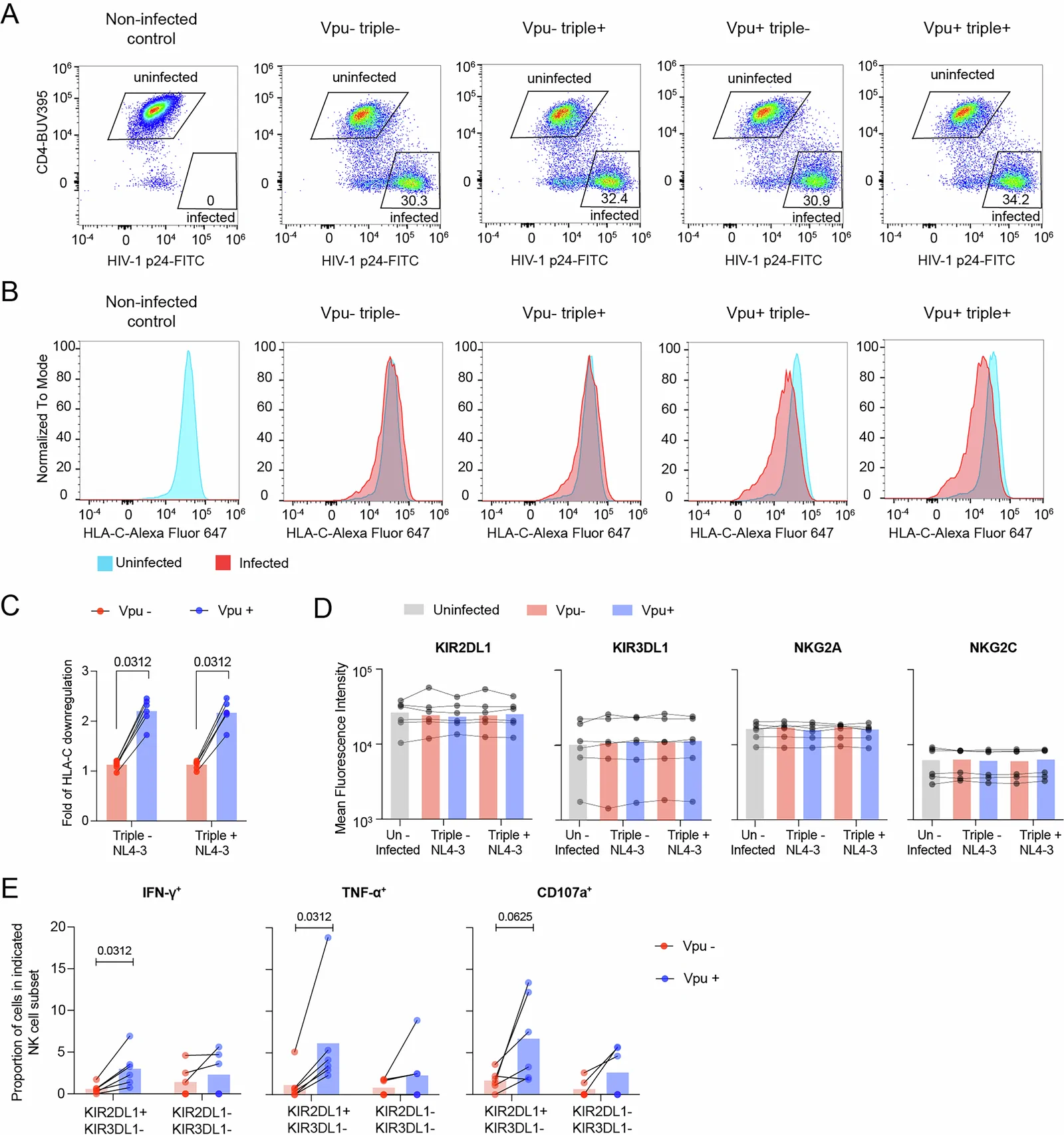
Functional assessments of IFN-γ secretion following NK cell stimulation with autologous CD4 T cells infected with HIV-1 NL4^−^3. **a**, Dot plots reflecting proportions of p24^+^ -enriched T cells, assessed after depletion with CD4 microbeads to enrich for HIV-1^−^infected cells, on day 5 following infection with indicated viral strains. **b**, Mode-normalized histograms depicting HLA-C expression on p24^+^ versus p24^−^ T cells on day 5 following infection with indicated viral strains. Data from one representative experiment are shown. **c**, Fold-difference in HLA-C surface expression in CD4 T cells infected with triple-positive or triple-negative NL4-3 containing Vpu sequences with (Vpu + ) or without (Vpu-) the ability to downregulate HLA-C. **d**, Mean fluorescence intensity of KIR2DL1, KIR3DL1, NKG2A and NKG2C in autologous NK cells after co-culture with p24^+^ -enriched CD4 cells. Data for uninfected CD4 T cells, and for CD4 T cells infected with the triple-positive or triple-negative NL4-3 viral constructs containing Vpu sequences with or without the ability to downregulated HLA-C. **e**, Proportions of KIR2DL1^+^ KIR3DL1^−^ and KIR2DL1^−^ KIR3DL1^−^ NK cells expressing IFN-γ, TNF-α or CD107a following co-incubation with CD4 T cells infected with NL4-3 containing Vpu sequences with (Vpu + ) or without (Vpu-) the ability to downregulate HLA-C. Data from n = 6 HLA-C2 + CMV+ healthy donors are shown. Significance was tested using a two-tailed Wilcoxon matched-pairs signed rank test. Source data

In a subsequent analysis, we mechanistically investigated effects of the sequence variants identified within these HLA-E-restricted HIV-1 epitopes. Using HLA-E stabilization assays with HLA-E-transfected K562 cells, we observed that variants in the RLVDGFLAL and KALGPAATL epitopes reduced HLA-E surface stability compared to their wild-type counterparts, potentially suggesting diminished HLA-E binding affinity; however, these differences were apparent only at high peptide concentrations (Fig. 4d). Similar findings were made when HLA-E-transfected RMA-S cells, derived from a TAP-deficient murine T cell lymphoma cell line, were used for HLA-E stabilization assays and, in the case of the RLVDGFLAL epitope, when ELISA-based assays^68^ were conducted to evaluate peptide binding to HLA-E (Extended Data Fig. 8a–c). By contrast, for the RPEPTAPPE and SEELRSLYN epitopes, pulsing with wild-type or variant peptides induced only minimal changes in HLA-E surface stability, likely because HLA-E interactions with HIV-1 peptides can be transient^69^ and may be difficult to capture in vitro (Fig. 4d and Extended Data Fig. 8c). Thus, while we observed peptide-variant effects for HLA-E stabilization in two cases, our data collectively indicated that HIV-1-derived peptides presented by HLA-E form relatively low-affinity complexes, consistent with prior reports^47,65,70^. Such low-affinity configurations, however, do not preclude biologically relevant recognition by NKG2C, as this activating receptor can tolerate, and potentially favor, low-affinity HLA-E conformers^62^. To further examine the functional effects of sequence variations in HLA-E-restricted viral epitopes, we generated viral NL4-3 constructs encoding either wild-type sequences within the three described HLA-E-restricted Gag epitopes (designated the ‘triple-negative virus’) or the corresponding variants (‘triple-positive virus’). Because viral recognition of HIV-1-infected CD4^+^ T cells by NK cells is strongly influenced by viral protein U (Vpu)-mediated downregulation of HLA-C^71,72^, which creates the ‘missing-self’ activation signal sensed by KIR2DL1^+^ NK cells in C2 donors, both viral constructs were engineered to contain either a functional Vpu sequence (from the primary isolate WITO, previously shown to effectively downregulate HLA-C^71^) or a Vpu sequence that is unable to downregulate HLA-C (as in the original NL4-3 construct). When primary NK cells were exposed to autologous CD4^+^ T cells infected with these constructs, no difference in recognition by KIR2DL1^+^NKG2C^+^ NK cells—measured by IFN-γ secretion—was observed between triple-negative and triple-positive viruses harboring the Vpu sequence that does not downregulate HLA-C (Fig. 4e). However, in the presence of a Vpu sequence capable of inducing HLA-C downregulation (Extended Data Fig. 9a–c), recognition of infected cells by KIR2DL1^+^ NK cells was enhanced (Extended Data Fig. 9e), and this effect was further amplified for the triple-negative virus in KIR2DL1^+^NKG2C^+^ NK cells (Fig. 4e). No HLA-E-restricted peptide-specific effects were detected in KIR2DL1^+^NKG2C^−^ or KIR2DL1^−^NKG2C^−^ NK cell subsets, consistent with our hypothesis of NKG2C involvement in variant-specific recognition of HLA-E epitopes (Fig. 4e). Together, these findings support a model in which HLA-E-restricted HIV-1 epitopes drive NKG2C-dependent activation of NK cells in a peptide-variant-specific manner, although the use of viral constructs incorporating multiple epitope variants precludes us to resolve whether the observed NK cell responses were driven predominantly by a single epitope or arise from additive or epistatic interactions among multiple epitopes. Nonetheless, our findings suggest that effective NKG2C-mediated activation appears to require concurrent missing-self-recognition signals mediated through KIR2DL1, providing a mechanistic explanation for why sequence variations in HLA-E epitopes preferentially influence HIV-1 reservoir size in individuals with KIR2DL1–HLA-C2-dependent NK cell education.

### Vpu sequence variations influence HIV-1 reservoirs

**Fig. 5:**
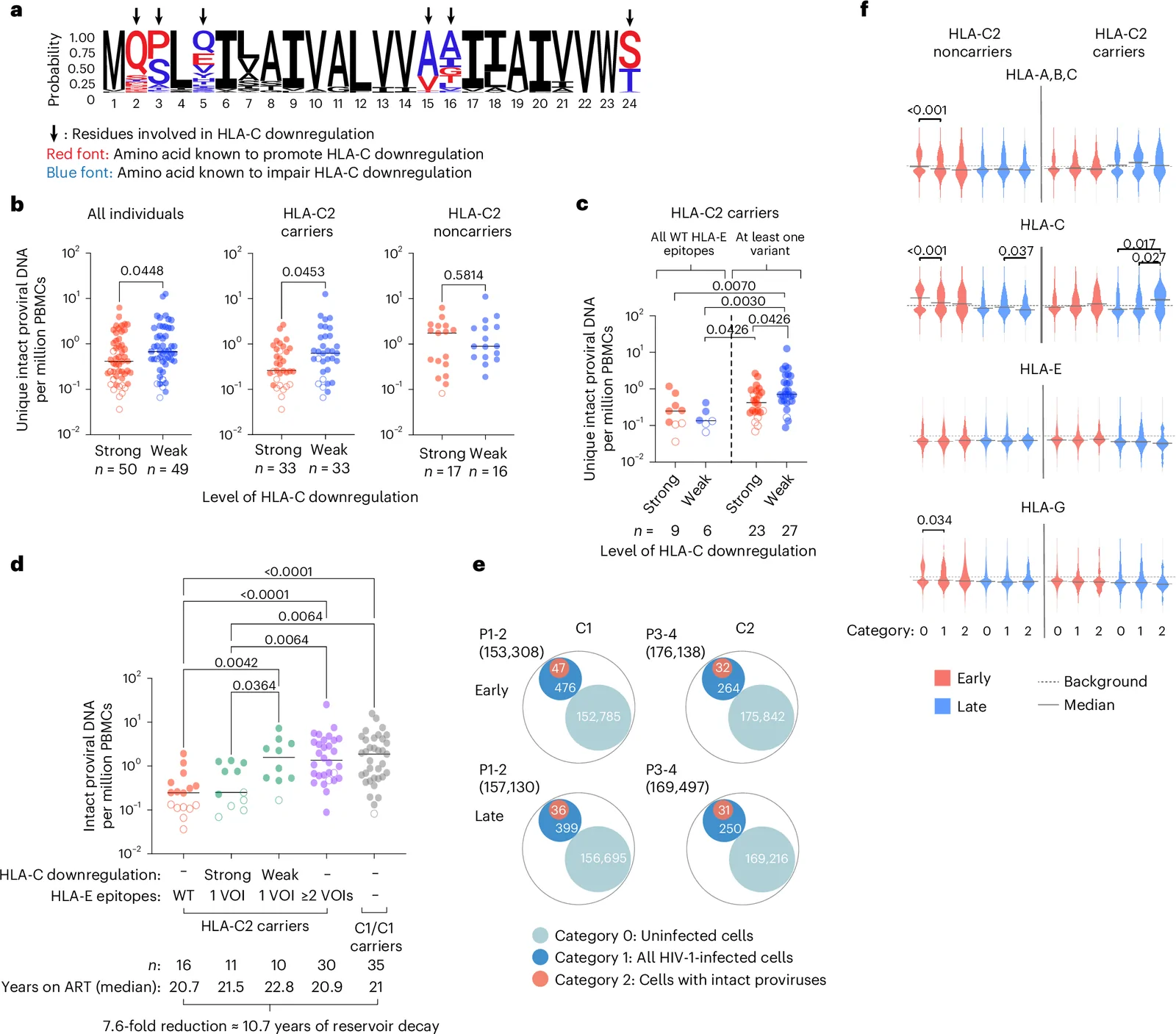
Imprints of NK-cell-dependent immune activity in HIV-1 reservoir cell landscape. **a**, Probability-weighted sequence logo of the first 24 amino acids in Vpu demonstrates the frequency of each indicated amino acid at designated positions, based on a multiple sequence alignment of all Vpu sequences from near-full-length proviruses available from *n* = 99 study persons on long-term ART. Five participants from the original cohort of *n* = 104 were excluded due to absence of detectable near-full-length proviruses or of complete Vpu sequences. Amino acid residues in red are known to effectively downregulate HLA-C; amino acids in blue have poor or absent abilities to downregulate HLA-C. **b**, Frequencies of unique intact proviruses in individuals harboring Vpu variants with 0–2 amino acids (weak) or 3–5 amino acids (strong) known to promote HLA-C downregulation. Data are shown for all individuals, and selectively for HLA-C2^+^ or HLA-C2^−^ study subjects. **c**, Frequencies of unique intact proviruses stratified according to presence of Vpu variants with strong (3–5 favorable Vpu amino acids) or weak (0–2 favorable amino acids) abilities to downregulate HLA-C, and according to the presence or absence of variant amino acids in HLA-E-restricted HIV-1 epitopes described in Fig. 4. **d**, High-level comparison of frequencies of intact proviruses in carriers of indicated host and viral genetic variants in HLA-C, Vpu and HLA-E-restricted HIV-1 epitopes. Hyphens (-) indicate that no further subgrouping was performed based on polymorphisms in Vpu or HLA-E-restricted HIV-1 epitopes. VOIs, variants of interest described in Fig. 4a. Open circles represent data at the limit of detection. Horizontal lines reflect the median. Significance was tested using a two-tailed Mann–Whitney *U* test for comparison of two groups (**b**) and Kruskal–Wallis with FDR-corrected multiple comparison test (**c**,**d**). **e**, Venn diagrams reflecting numbers of cells investigated by single-cell proteogenomic profiling assays early (approximately 1 year) and late (17–20 years) after ART initiation in homozygous carriers of C1 and C2. P1-2 and P3-4 refer to participants 1 and 2 and participants 3 and 4, respectively. **f**, Violin plots (indicating the median) reflecting the surface expression of indicated HLA class I molecules on all uninfected memory CD4^+^ T cells (category 0), memory CD4^+^ T cells harboring any type of HIV-1 DNA (category 1) and memory CD4^+^ T cells harboring proviruses classified as genome-intact (category 2). Significance was tested using two-tailed Student’s *t*-tests. Source data

To assess the in vivo impact of KIR2DL1^+^NKG2C^+^ NK cells on the HIV-1 reservoir, we investigated whether genetic variations in Vpu correlate with proviral reservoir size. Prior studies^71,73^ have shown that the capacity of Vpu to downregulate HLA-C depends on amino acid residues 2, 3, 5, 15, 16 and 24 within the Vpu sequence, which interact with the HLA-C transmembrane domain to reduce surface expression (Fig. 5a). A higher number of Vpu residues known to downregulate HLA-C in autologous near-full-length proviruses correlated with lower frequencies of intact proviruses (Fig. 5b), consistent with NK-cell-mediated immune pressure on reservoir cells. This relationship was restricted to HLA-C2 carriers and was not observed in individuals lacking HLA-C2, suggesting that Vpu-mediated HLA-C downregulation preferentially sensitizes reservoir cells to clearance by KIR2DL1-educated NK cells. Combined analyses further supported a cooperative effect between Vpu-driven HLA-C downregulation and NKG2C–HLA-E-dependent NK cell activation in HLA-C2 carriers, as the largest reservoirs were observed in individuals harboring Vpu variants with limited HLA-C downregulation capacity together with variants in HLA-E-restricted epitopes exhibiting reduced NKG2C engagement (Fig. 5c). Conversely, higher reservoir sizes associated with unfavorable HLA-E epitope variants were partially mitigated by Vpu sequences retaining strong HLA-C downregulation activity. Consistent with these findings, a combined host–proviral genetic polymorphism analysis demonstrated that frequencies of intact proviruses between carriers of the most and the least favorable viral and host sequence polymorphisms in HLA-C, Vpu and HLA-E-restricted HIV-1 epitopes differed by more than sevenfold, equivalent to the decay of intact proviruses observed over 10 years of continuous ART, assuming a half-life of intact proviruses of 44 months^74,75^ (Fig. 5d). These results suggest that coordinated HLA-C-dependent education and NKG2C–HLA-E-dependent activation act synergistically to mediate NK-cell-driven reduction of HIV-1 reservoir cells in vivo.

### HLA-C expression on HIV-1 reservoir cells

For additional validation of in vivo NK-cell-driven immune effects against HIV-1 reservoir cells, we used a single-cell proteogenomic profiling assay, termed PheP-Seq in our prior work^15,76^, to track the longitudinal evolution of HLA-C surface expression on CD4^+^ T cells harboring intact HIV-1 proviruses. Specifically, we analyzed samples from two individuals homozygous for HLA-C1 and two individuals homozygous for HLA-C2, using PBMCs collected approximately one year and 17–20 years after the initiation of suppressive ART. In brief, memory CD4^+^ T cells were isolated and stained with oligonucleotide-tagged antibodies targeting surface proteins, followed by single-cell encapsulation and barcoding using a microfluidics platform. Each cell then underwent multiplex PCR to amplify 18 discrete HIV-1 genomic regions (200–300 bp each; totaling ~4,000 bp), including two sites previously shown to be predictive of viral genome intactness^77^. Amplification products were then pooled, sequenced and computationally deconvoluted to assign sequencing reads to their individual input cells. In total, we analyzed 310,438 and 345,635 memory CD4^+^ T cells from study participants carrying the HLA-C1 or HLA-C2 alleles, respectively. Based on established criteria, 958 and 577 of these cells were classified as HIV-1-infected cells harboring any form of provirus, while 83 and 63 cells were identified as containing intact proviruses in carriers of C1 or C2 alleles, respectively (Fig. 5e). This high-resolution analytical approach revealed no significant differences in HLA-C surface expression between CD4^+^ T cells harboring intact proviruses and uninfected total memory CD4^+^ T cells within one year of ART initiation, regardless of the HLA-C subgroup (Fig. 5f). However, after long-term ART, we observed that in carriers of HLA-C2, but not of HLA-C1, HLA-C surface expression was higher in CD4^+^ T cells harboring intact HIV-1 compared with the overall memory CD4^+^ T cell population. In contrast, surface expression of alternative classical and nonclassical HLA class I alleles did not differ between cells containing intact proviruses and the total memory CD4^+^ T cell pool at any time point (Fig. 5f). Interestingly, in HLA-C1 carriers, HIV-1-infected cells displayed reduced surface expression of HLA-A, HLA-B and HLA-C at one year after ART initiation, possibly suggesting adaptation to CTL-mediated immune selection pressure during early stages of ART; however, this was not detectable at later time points, consistent with limited effects of CTL on viral reservoir cells during long-term ART. Together, these findings support the hypothesis that heightened in vivo immune selection pressure in HLA-C2 carriers preferentially eliminates infected cells with low HLA-C expression, leading to the enrichment of reservoir cells with high HLA-C expression levels and an increased likelihood of evading immune-mediated clearance by KIR2DL1-educated NK cells.

## Discussion

Traditionally, persisting HIV-1 reservoirs have been viewed as a restricted cellular compartment that remains largely invisible to the immune system due to limited or absent viral gene expression, rendering it resistant to host immune responses. For the past one to two decades, this paradigm has strongly shaped the HIV cure field, underpinning the idea that latently infected cells must first be reactivated to become susceptible to immune clearance and driving the so-called shock and kill strategy^78^. Our analysis, to our knowledge one of the most comprehensive integrative analyses of proviral reservoir cell sequences, host immunogenetic factors and antiviral immune responses conducted so far, challenges this paradigm. Our findings suggest that instead, the size of the viral reservoir during long-term ART is regulated by a finely balanced interplay between infected cells and innate cellular immunity. In particular, our data suggest that NK cells educated via KIR2DL1, considered as the strongest driver of NK cell education due to the high avidity and low peptide selectivity of KIR2DL1–HLA-C2 interactions^79^, can exert effective immune selection pressure on the reservoir cell pool, specifically when concurrently activated through peptide-variant-specific NKG2C–HLA-E interactions. This model points to a distinct vulnerability of reservoir cells to NK-cell-mediated recognition, presumably at least in part driven by residual Vpu expression. Even at low levels, Vpu may downregulate HLA-C in reservoir cells when favorable viral residues are present, thereby generating a missing-self NK cell recognition signal on KIR2DL1^+^ NK cells that can then be further amplified through NKG2C-dependent recognition of HLA-E-restricted HIV-1 epitopes. Of note, it is also plausible, if not likely, that differential recognition of HLA-E-restricted CMV epitopes^61^ further influenced activation of KIR2DL1^+^NKG2C^+^ NK cell populations in our study participants; however, our study design did not allow for an assessment of CMV sequence variations to more definitively investigate this.

Our proposed model does not diminish the importance of HIV-1-specific T cells in recognizing and eliminating HIV-1-infected cells, particularly during untreated infection or treatment interruptions, when full viral replication cycles occur and T cell-driven immune pressure shapes viral sequence evolution within targeted epitopes^80^. Rather, our findings suggest a functional dichotomy in which HIV-1-specific T cell responses dominate immune selection during active viral replication off ART, whereas NK-cell-mediated mechanisms become the prevailing form of immune pressure on the viral reservoir under long-term suppressive ART. Notably, our study focused on individuals with very long durations of antiretroviral treatment (median duration of 21 years), and we excluded participants with shorter treatment durations (<10 years). Whether NK cells exert measurable selection pressure on reservoir cells during earlier phases of ART remains unclear and will require investigation in future longitudinal studies. It is also conceivable that mechanisms of immune selection shift progressively over the course of ART, with HIV-1-specific T cells possibly representing a driving force of immune selection during earlier stages of ART, when many proviruses are integrated in genic regions and display higher levels of transcriptional activity^7,12^, potentially enabling immune recognition by T cell receptor-dependent mechanisms at this time. Cells that survive and persist beyond this phase may do so by acquiring immune-evasive features such as HLA class I downregulation; these adaptations, while protective against cytotoxic T lymphocytes, might reciprocally confer increased vulnerability to NK-cell-mediated elimination, effectively shifting the dominant form of immune pressure to NK cells as treatment continues. Such a cooperative dynamic between T cells and NK cells, each exerting complementary but temporally distinct selection pressures on the viral reservoir, warrants further exploration in future longitudinal studies. The precise role of NK cells educated by mechanisms other than HLA-C2–KIR2DL1, such as NK cell education via HLA-Bw4–KIR3DL1 and HLA-E–NKG2A, at earlier stages of ART also merits additional investigation. Of note, the findings presented are concordant with prior studies suggesting a role for KIR-educated NK cells in posttreatment controllers^27^, and additional future studies exploring the role of NK cells in immune-mediated posttreatment control of HIV-1 replication would likely be highly informative.

While our data suggest heightened immune activity of KIR2DL1^+^NKG2C^+^ NK cells against persisting HIV-1-infected cells, consistent with the promising therapeutic activity of single-KIR^+^ adaptive NK cells against KIR–HLA-mismatched leukemia cells^48,49,81,82^, we emphasize that they cannot eradicate viral reservoir cells; rather, surviving reservoir cells appear to adapt by sustaining high-level HLA-C expression, enabling resistance to KIR2DL1^+^ NK cell clearance. Elimination of such a highly selected reservoir cell pool will likely require more targeted interventions that specifically disassemble this protective layer of the reservoir, possibly through pharmacological manipulation of KIR2DL1–HLA-C2-dependent immune checkpoints^83,84^; however, we hypothesize that the profound resilience of HIV-1 reservoir cells may require simultaneous targeting of additional viral vulnerabilities. Importantly, our data strongly supports the hypothesis that viral reservoir cell persistence is heavily influenced by genetic variability within both the host genome and proviral sequences, suggesting that larger cohort studies focusing on integrated assessments of viral and host genetic diversity will yield deeper insight into vulnerabilities and susceptibilities of the reservoir cell pool. When sufficiently powered, such studies may help to disentangle more complex immunogenetic interactions between viral and host sequence variations, including combinatorial effects that were beyond the scope of our study, and may also reveal specific reservoir cell dynamics more definitively associated with age and sex^85^. Ultimately, broader characterizations of viral reservoirs, integrated with evaluations of host and viral genetic variation, will enable more personalized assessments of reservoir biology and may help identify individuals most likely to benefit from precision interventions tailored to specific viral and host factors.

## Methods

### Study participants

Study participants living with HIV-1 on ART were recruited at Massachusetts General Hospital, Brigham and Women’s Hospital, the University of California at San Francisco, Case Western Reserve University, the Women’s Interagency HIV Study (WHIS) and the AIDS Clinical Trials Group (ACTG protocol A5321). No formal sample size calculations, randomizations or investigator blinding operations were performed. Clinical and demographic characteristics of the study participants are summarized in Supplementary Table 1. All study participants provided written consent in accordance with the Declaration of Helsinki before enrolment. Depending on the individual source protocols, study participants were eligible to receive a small stipend for transportation and meal expenses. PBMC samples were collected according to protocols approved by the respective Institutional Review Boards. Data from treatment-naive PLWH were available from a total of *n* = 4,007 study persons from the following US cohorts^44^: AIDS Clinical Trials Group (ACTG, https://actgnetwork.org), ALIVE, HIV Natural History Study (DoD HIV NHS, http://idcrp.usuhs.edu), Ragon Institute Controller Cohort (https://www.broadinstitute.org/hiv-controllers/international-hiv-controllers-study), Study on the Consequences of Protease Inhibitor Era (SCOPE, https://clinicaltrials.ucsf.edu/trial/NCT00187512). Each participant approved the use of their DNA samples for genetic analysis.

### Near full-length proviral sequencing

Genomic DNA from study participants was subjected to HIV-1 near-full-genome amplification using a non-multiplexed single-amplicon as described previously^28,86^. All near-full-length PCR products were subjected to Illumina MiSeq sequencing at the MGH DNA Core facility. Resulting Illumina short reads were de novo assembled using Ultracycler v1.0 and aligned to HXB2 to identify large deleterious deletions (<8,000 bp of the amplicon aligned to HXB2), out-of-frame indels, premature or lethal stop codons, internal inversions, or packaging signal deletions (≥15 bp insertions and/or deletions relative to HXB2), using an automated in-house pipeline written in Python programming language (https://github.com/BWH-Lichterfeld-Lab/Intactness-Pipeline). The presence or absence of APOBEC-3G- and 3F-associated hypermutations was determined using the Los Alamos HIV Sequence Database Hypermut 2.0 program. Viral sequences that lacked all mutations listed above were classified as ‘genome-intact’. Sequence alignments were performed using MAFFT in Geneious Prime software v2023.1 (https://www.geneious.com). Phylogenetic distances between sequences were examined using a PhyML tree^87^ in Geneious Prime software. Viral sequences were considered clonal if they had completely identical consensus sequences; single-nucleotide variations in primer binding sites were not considered for clonality analysis. For HIV-1 reservoir quantification, only sequences generated by a single-amplicon PCR approach were included in the analysis. In addition, proviral sequencing data generated by the five-amplicon approach, preceded by whole-genome amplification reactions^86^, were used when available in selected patients for analyzing proviral sequence diversity. For HIV-1 sequence polymorphism analysis, both HLA-E epitope and Vpu variant calling was performed using genome-intact proviruses; however, for individuals with fewer than three intact proviral sequences, both intact and available near-full-length defective sequences were analyzed to ensure that variant assessment accurately reflected the proviral sequence diversity of each study participant.

### HLA class I and KIR typing

High-resolution HLA class I and KIR typing were performed using a targeted next-generation sequencing method, as previously described^88^.

### Serologic testing for CMV

Testing for CMV IgG antibodies was performed using Arlington Scientific Cytomegalovirus IgG ELISA Test Kit (Cat#22-415-207) or the Abbexa Cytomegalovirus IgG Rapid Test Kit (Cat#ABIN5708603), per the manufacturer’s instructions.

### Phenotypic analysis of NK cells by flow cytometry

**Extended Data Fig. 10:**
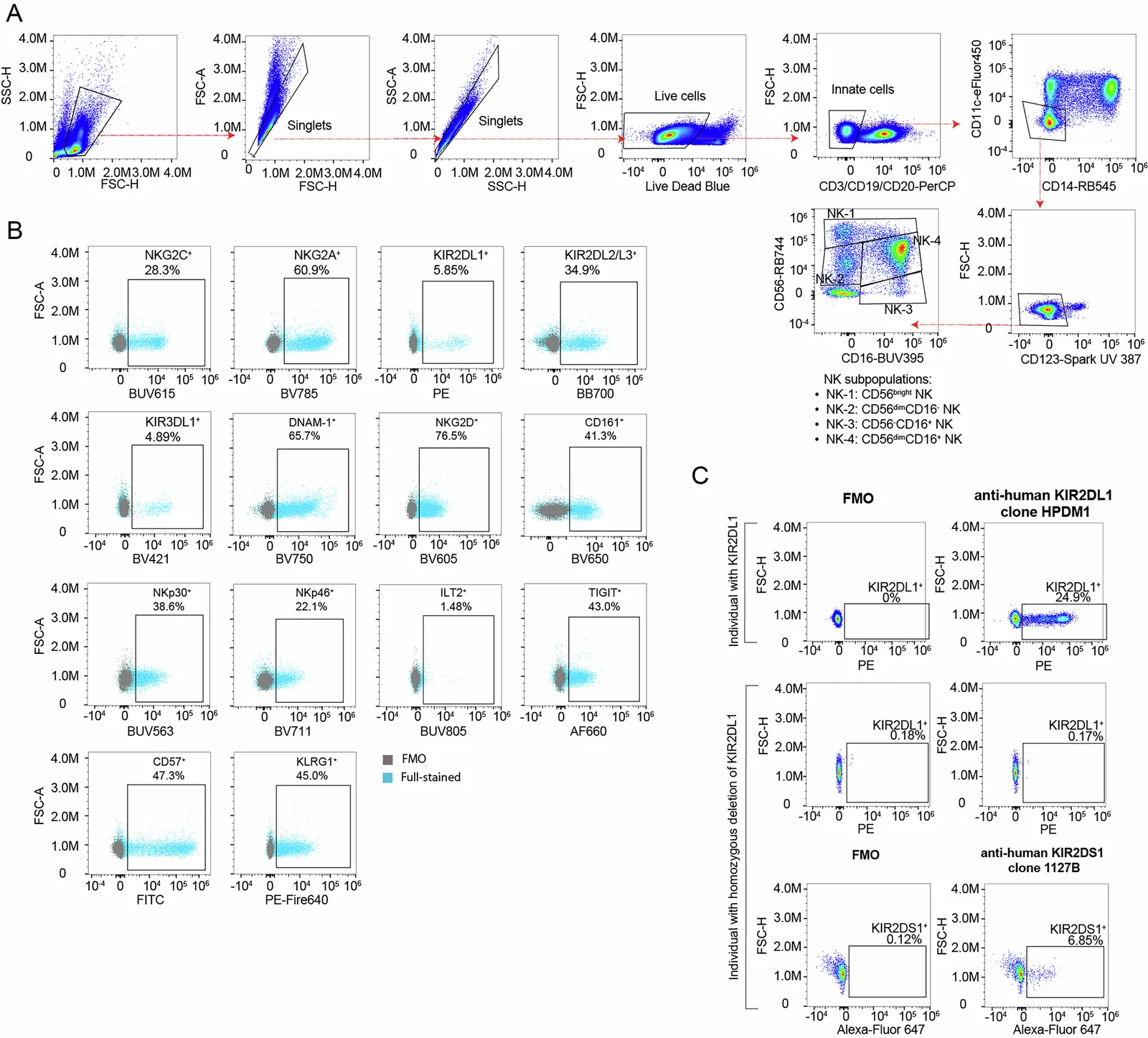
Flow cytometric analysis of NK cells. **a,b**, Flow cytometric gating strategy for analysis of NK cells. **c**, Specificity of the HP-DM1 antibody for detection of KIR2DL1: Staining of NK cells with the KIR2DL1^−^specific antibody (clone HP-DM1) in a study person who is homozygously negative for KIR2DL1; in the same study persons, staining with the KIR2DS1^−^specific antibody clone 1127B revealed a clear signal, indicating that clone HP-DM1 is specific for KIR2DL1 and does not cross-recognize KIR2DS1.

PBMCs were thawed, stained with LIVE/DEAD Blue Viability Dye (Invitrogen) for 10 min and subsequently preincubated for 10 min with an Fc receptor (FcR) blocking reagent (Miltenyi Biotec). Afterward, cells were incubated for 25 min with different combinations of appropriately titrated antibodies directed against NK cell markers as listed in Supplementary Table 4. Subsequently, the cells were fixed in 2% paraformaldehyde in phosphate-buffered saline (PBS) and acquired on an Aurora spectral cytometer (Cytek Biosciences) at the Ragon Institute Flow Cytometry Core Facility. Data were analyzed using FlowJo v10.9.0 software (Tree Star LLC). Gating strategies are summarized in Extended Data Fig. 10a. Fluorescence minus one samples were prepared and used as part of the gating strategy (Extended Data Fig. 10b).

### Functional analysis of antigen-specific T cells

To analyze HIV-1-specific T cell responses, PBMCs were rested in R10 medium for 4 hours at 37°C in 5% CO_2_ and then incubated with an HIV-1 consensus clade B Gag peptide pool (mix of 123 overlapping 15-mer peptides at 2 µg ml^−1^ for each peptide, NIH AIDS Reagent Program #ARP-12425) or Peptivator CMV pp65 and IE-1 at 0.1 µg ml^−1^ each (130-093-435 and 130-093-493, Miltenyi Biotec) in the presence of anti-CD28 and anti-CD49d at 1 µg ml^−1^ (BD Biosciences) and antibodies against CD107a and CD107b (clones H4A3 and H4B4, respectively, BioLegend). After an hour, brefeldin A (5 µg ml^−1^, BioLegend) and monensin (1 µg ml^−1^, BD Biosciences) were added, and cells were incubated further overnight. An unstimulated negative control that lacked antigenic peptides but was otherwise treated identically was included for each sample. After stimulation, cells were washed and stained with LIVE/DEAD Blue Viability Dye (Invitrogen), followed by preincubation with 2 µl of FcR blocking reagent and surface staining for 25 min with appropriately titrated antibodies directed against T cell surface markers, as listed in Supplementary Table 5. Subsequently, cells were washed, fixed and permeabilized using the FoxP3 transcription factor buffer kit (eBioscience) for 30 min at 4°C. Intracellular cytokine staining was performed with appropriately titrated antibodies directed against intracellular cytokines, as listed in Supplementary Table 5. The cells were fixed in 2% paraformaldehyde in PBS and acquired on a Cytek Aurora spectral cytometer (Cytek Biosciences). Fluorescence minus one samples were prepared and used as part of the gating strategy. Unstimulated controls were run for each sample and subtracted as background. Data were analyzed using FlowJo v10.9.0 and the Simplified Presentation of Incredibly Complex Evaluations (SPICE) software v6.0^89^. HIV-1 Gag-specific CD8^+^ T cell polyfunctionality was assessed using the Combinatorial Polyfunctionality Analysis of Antigen-Specific T-cell Single Subsets (COMPASS) framework implemented in R v4.5 (COMPASS Bioconductor package)^90^.

### HLA-E stabilization assays

Selected nonameric peptides corresponding to previously described HLA-E-restricted epitopes in HIV-1^47,65,91^ and their respective variants were synthesized by a commercial supplier at a purity of ≥98%. K562-HLA-E*01:03 (kindly provided by Dr. Michael Birnbaum, MIT) were pulsed with individual synthetic peptides in 4-fold serial dilutions, ranging from 0.39 to 400 µM, for 16 hours at 26°C in Opti-MEM™ I reduced-serum medium. Cell surface expression levels of HLA-E were assessed by flow cytometry staining using a BV421-labeled anti-HLA-E antibody (clone 3D12). The CMV UL40–HLA-Cw3 leader-sequence-derived peptide VL9 (VMAPRTLIL) was used as a positive control, while NLVPMVATV, a CMV-pp65-derived nonamer that does not stabilize HLA-E, was used as a negative control. Cells were acquired on an Aurora spectral cytometer (Cytek Biosciences) at the Ragon Institute Flow Cytometry Core Facility. The area under the curve (AUC) values of each peptide-specific stabilization assay are summarized in Supplementary Table 6.

### ELISA-based HLA-E binding assays

Peptide loading of peptide-free HLA-E complexes was performed as previously described, with minor modifications^68^. Briefly, peptide-free HLA-E–β2-microglobulin (β2M) complexes were generated by macro-refolding in the absence of peptide ligand. Refolding buffer consisted of 100 mM Tris-HCl (pH 8.0), 2 mM EDTA, 5 mM reduced glutathione and 0.5 mM oxidized glutathione. Peptide-loading was performed in polypropylene V-bottom 96-well plates (Greiner Bio-One) and incubated for 3 h on ice. Peptide-loaded HLA-E complexes were quantified using a sandwich ELISA as previously described^64,68,92^. High-binding 96-well plates (Life Technologies) were coated overnight at 4°C with anti-HLA-E monoclonal antibody (clone 3D12; BioLegend) at 10 μg ml^−1^ in coating buffer (BioLegend), washed in PBS and blocked with 2% IgG-free BSA (Sigma-Aldrich) for 2 h at room temperature. Peptide loading reactions were diluted 1:100 in 2% BSA, and 50 μl was added per well before incubation for 1 h at room temperature. Wells containing VL9 peptides were spatially separated to prevent cross-contamination. Following washing (0.05% Tween-20 wash buffer; BioLegend), bound complexes were detected using HRP-conjugated rabbit anti-human β2M antibody (Thermo Fisher Scientific, 0.2 μg ml^−1^) for 30 min at 4°C. Signal was amplified using goat anti-rabbit HRP-conjugated IgG (EnVision+ System-HRP; Agilent; 1:15 dilution in 2% BSA containing 1% mouse serum) for 15 min at room temperature. Plates were developed using TMB substrate (BioLegend) for 10 min, stopped with H_2_SO_4_ stop solution (BioLegend); absorbance was measured at 450 nm.

### Viral constructs

Plasmids of HIV-1 NL4-3 (pNL4-3) and Chimera 5 (pM4-5), in which a 141 bp fragment of the *vpu* sequence derived from the primary HIV isolate WITO was inserted into the NL4-3 genome, were described before^71^ and kindly provided by Dr. Gregory Del Prete (NIH). Base pair variants in HLA-E-restricted Gag epitopes were inserted in the pNL4-3-dEnv plasmid, a kind gift from N. Manel and D. Littman (New York University, New York City, USA)^93^, to create Gag variant Y79F in the SN9 epitope, Gag variant A340G in the KL9 epitope and Gag variant E460A in the RE9 epitope by a commercial laboratory (Azenta). Plasmids containing all three of these HLA-E epitope variants associated with higher HIV-1 reservoir size or all three of the corresponding epitope sequences associated with lower HIV-1 reservoir size were then digested with the restriction enzymes BssHII and SbfI, and the resulting *gag* fragments were ligated into the pNL4-3 and pM4-5 backbones to generate four plasmids. Viral particles were produced by transfecting 293 T cells with the respective HIV-1 plasmids using Lipofectamine™ 3000 Transfection Reagent (Thermo Fisher Scientific) in Opti-MEM™ I reduced-serum medium per the manufacturer’s instructions. Supernatants containing infectious retroviruses were harvested 48 hours after transfection, quantified with the Lenti-X™ p24 Rapid Titer Kit (#631476, Takara) and stored at −80°C.

### Functional NK cell assays

Primary CD4^+^ T cells isolated by negative immunomagnetic enrichment kit (Miltenyi Biotec) were stimulated in RPMI medium supplemented with 10% fetal calf serum and recombinant IL-2 (100 IU ml^−1^) for three days with ImmunoCult™ Human CD3/CD28 T Cell Activator (#10971, Stemcell) at 25 μl ml^−1^, and then infected with the designated viral constructs at a multiplicity of infection of 0.1 for six hours. After 2 washes, cells were resuspended in medium and plated at 3–5 × 10^5^ cells per well in a 24-well plate. Five days later, infected cells were enriched by depletion of CD4^+^ T cells using a positive immunomagnetic selection kit (#17852, Stemcell) and tested for HLA-C surface expression as described in the ‘Flow cytometric assessment of HLA-C expression’ section. Subsequently, cells were mixed with autologous NK cells (isolated by an immunomagnetic enrichment kit, # 130-092-657, Miltenyi Biotec) that had been cultured with recombinant human IL-15 (10 ng ml^−1^) overnight at an effector to target ratio of 1:1 for six hours. Brefeldin A (5 μg ml^−1^) and monensin (1 μg ml^−1^) were added one hour after the beginning of the coculture assay, followed by an additional 5 hours of incubation. Cells were stained with viability dye and antibodies against CD3, CD56, KIR2DL1, KIR3DL1, NKG2C and NKG2A. Cells were then washed, fixed and permeabilized using the FoxP3 transcription factor staining buffer set (eBioscience) for 30 min at 4°C before being subjected to intracellular cytokine staining with antibodies against IFN-γ. Cells were acquired on an Aurora spectral cytometer (Cytek Biosciences) at the Ragon Institute Flow Cytometry Core Facility.

### Flow cytometric assessment of HLA-C expression

To evaluate HLA-C expression on infected and uninfected CD4^+^ T cells, cells were first surface labeled with CD8, CD19, CD20, CD4 and HLA-C (clone DT9) antibodies. Cells were then washed, fixed and permeabilized using the FoxP3 transcription factor staining buffer set (eBioscience) for 30 min at 4°C. Intracellular staining of HIV-1 p24 was then performed using FITC-labeled KC57 antibody against HIV core antigen (#6604665, Beckman Coulter). Following additional washes, cells were acquired on an Aurora spectral cytometer (Cytek Biosciences) at the Ragon Institute Flow Cytometry Core Facility to evaluate the surface expression of HLA-C on p24-positive cells.

### Single-cell proteogenomic profiling of NK cells

NK cells were isolated by negative immunomagnetic enrichment (Miltenyi Biotec), blocked with Human TruStain FcX (#422301; BioLegend) for 15 min on ice and then incubated with oligonucleotide-tagged monoclonal antibodies carrying the Totalseq-D modification (BioLegend) for 30 min on ice. CD16, CD56, KIR2DL1, KIR3DL1, NKG2C and NKG2A antibodies were used, in addition to a panel of additional oligonucleotide-labeled antibodies described in our previous work^76^. Two mouse IgG control antibodies (#400299, #400383; BioLegend), conjugated with distinct TotalSeq-D oligonucleotides were included as isotype controls. After three washes with a prechilled cell-staining buffer, cells were filtered with a 40-μm cell Flowmi strainer (#14-100-150; Thermo Fisher Scientific), counted with an automated cell counter and then loaded into a microfluidic cartridge of the Tapestri analysis platform (Mission Bio) for single-cell multiplex PCR assays. Single-cell amplification of mitochondrial DNA was then performed according to the manufacturer’s protocol^94^, with minor modifications described in our previous work^15,76^. Briefly, viable single cells were encapsulated into droplets and lysed. Droplets containing single-cell barcoding beads (tagged with oligonucleotides carrying the cellular barcodes and custom-designed primers) were fused with encapsulated cell lysates. A panel of primers designed to amplify *n* = 70 adjacent, but nonoverlapping regions of the mitochondrial DNA was then added; primer sequences are summarized in Supplementary Table 7. The droplets were placed under UV light to cleave PCR primers containing unique cell barcodes from beads. To amplify the selected genomic DNA segments and the antibody oligonucleotide tags, droplets were subjected to PCR for 24 cycles with temperature gradients recommended by the manufacturer. Amplification products were then pooled, mixed with AMPure XP beads (#A63882; Beckman Coulter) at a ratio of 0.7, and placed in a magnetic field for separating the DNA and the protein tag libraries. The DNA library bound to AMPure beads was washed with 80% ethanol, while the supernatant containing the protein tag library was aspirated and incubated with a biotinylated oligonucleotide complementary to the 5′ end of the antibody tags, followed by magnetic isolation using streptavidin beads. For library amplification, PCRs were performed with Illumina index primers P5 and P7 on purified DNA and protein libraries, respectively, according to the manufacturer’s protocol; 12 cycles were performed for the DNA library and 18 cycles were run for the protein tag library. The DNA and protein tag libraries were run on a High Sensitivity D1000 ScreenTape instrument (5067-5584; Agilent Technologies) with the Agilent 4200 TapeStation System to evaluate DNA quality. Libraries were quantified by a fluorometer (Qubit 4.0; Invitrogen) and sequenced on Illumina next-generation sequencing platforms with a 20% spike-in of PhiX control DNA (#FC-110-3002; Illumina). DNA libraries were sequenced on a NextSeq2000 instrument (Illumina), using NextSeq 2000 P3 Reagents (300 Cycles) (# 20040561; Illumina), and protein tag libraries were sequenced separately on a NovaSeq X Plus instrument (Illumina), both in 2 × 150 bp paired-end runs.

### Single-cell proteogenomic profiling of CD4^+^ T cells

For single-cell proteogenomic profiling of HIV-1-infected CD4^+^ T cells, total memory CD4^+^ T cells isolated by negative immunomagnetic enrichment (#19157, Stemcell) were labeled with oligonucleotide-tagged antibodies carrying Totalseq-D modification (BioLegend). The following markers were analyzed in this study: HLA-E (clone 3D12), HLA-C (clone DT-9), HLA-G (clone 87 G), HLA-A, B, C (clone W6/32), CD4 (clone RPA-T4), CD3 (clone UCHT1), CCR7 (clone G043H7), CD45RA (clone HI100); two mouse IgG control antibodies (#400299, #400383; BioLegend), conjugated with distinct TotalSeq-D oligonucleotides, were included as isotype controls. Afterwards, viable cells were entered into a microfluidic cartridge for single-cell multiplex PCR assays, as described in our previous work^15^. Single-cell PCR was conducted with 18 primer pairs designed to amplify 18 ~200–300 bp segments in proviral HIV-1 DNA, including primers corresponding to the two target regions of the IPDA^77^. Primer sequences were adjusted to autologous proviral sequences obtained before, and are listed in our previous work^15^. Two primer sets amplifying control genomic regions within the RPP30 gene on chromosome 10 were also included. Single-cell PCR conditions and construction of DNA and protein libraries were performed similarly to those for single-cell proteogenomic profiling of NK cells.

### Bioinformatics analysis

The Tapestri pipeline (v3.4.0 for NK cells and v2.0.1 for CD4^+^ T cells; MissionBio) with minor modifications was used to process sequencing data generated by single-cell proteogenomic profiling assays, as described in our prior work^15,76^. Briefly, for DNA library data, cutadapt (v2.5)^95^ was used to trim 5′ and 3′ adapter sequences and extract 18 bp cell barcode sequences from Read 1. Cell barcodes that aligned to a unique barcode on a whitelist within a Hamming distance of 2 were used for downstream analysis. Using bwa-mem2 (v2.2.1)^96^, sequences were aligned to custom reference genomes built from the human genome (hg38), the mitochondrial genome (NC_012920.1) or participant-specific autologous HIV-1 sequences identified in near-full-length proviral sequencing assays, as appropriate. Single-cell alignments were filtered according to criteria implemented in the Tapestri pipeline, and indexed using samtools (v1.19)^97^. Candidate cells were identified by the CellFinder algorithm built in the Tapestri pipeline. Samtools (v1.19)^98^ was used to call variants and generate consensus sequences. To reduce spurious alignments, viral sequencing reads were only considered valid if they covered at least 80% of the length of the reference sequence for each given amplicon. MGATK^58^ was used to call sequence variants in mitochondrial DNA and to calculate the corresponding variant allele frequencies to estimate the level of heteroplasmy; subsequently, scMitoMut^99^ was utilized to filter and identify mitochondrial mutations that are phylogenetically informative for calling clonal cell lineages. Assessment of NK cell clonality was restricted to cells with no more than one or two high-confidence somatic mutations (VAF ≥ 50%), retaining variants observed in at least five cells. Within these cells, NK cells were considered clonal when they shared the dominant (VAF > 50%) nucleotide variant at polymorphic positions. For identifying cells harboring genome-intact, defective or no HIV-1 DNA, the same criteria were used as described in our prior work^15^; resulting proviral sequence callings were manually reviewed and edited based on available autologous proviral phylogenetic information.

For the analysis of protein library data, cell barcodes were extracted using cutadapt (v2.5). For reads with valid cell barcodes, 15-bp antibody barcodes were extracted from Read 2. Antibody barcode sequences within a Hamming distance of 1 from known antibody barcodes were accepted. Candidate cells appearing in both libraries were processed for downstream multiomic analysis integrating genotypic and phenotypic data. Read counts for each antibody were normalized to the total read count within each cell using a centered log-ratio (CLR) transformation^100^. Cutoffs for a given phenotypic marker to be considered positive were defined by marker-specific read counts >1 mean absolute deviation of the normalized median read count corresponding to unspecific IgG control antibodies. To generate a more homogenous cell population for analysis of HIV-1 reservoir cells, CLR values of all candidate cells that were CD3^−^CD4^−^ (not CD4^+^ T cells) or CCR7^+^CD45RA^+^ (contaminating naive CD4^+^ T cells) were excluded from proteogenomic CD4 T cell data. Phenotypic analysis was performed using the monocle3 R package^101^. Antibody read counts were log-transformed, and cells displaying abnormally high IgG read counts or elevated CD3 values were excluded to improve data quality and achieve a more homogeneous cell population. Cells were embedded in two dimensions using UMAP^102^. To quantify and visualize marker expression of NK cells, median expression values per NK cell clone were calculated and scaled by the mean and standard deviation of cluster means.

### Statistics

Data are presented as pie charts, bar charts, scatter plots with individual values, UMAP plots, violin plots, Venn diagrams and heatmaps. Differences were tested for statistical significance using two-sided Wilcoxon matched-pairs signed rank tests, two-sided Mann–Whitney *U* tests, two-sided Kruskal–Wallis nonparametric tests, two-sided χ tests or Spearman correlation tests, as appropriate. *P* values of <0.05 were considered statistically significant; FDR correction was performed as described^103^. Potential synergistic effects between the HLA-C2 allele and specific HLA-E-restricted epitope variants on HIV DNA levels were evaluated using linear regression models incorporating explicit multiplicative interaction terms. Analyses were performed using Prism v10.6 (GraphPad Software, Inc.) and R v4.5^104^. For evaluating the influence of immunogenetic markers on HIV-1 viral loads and reservoir size (Tables 1 and 2), linear regressions with the function lm() were used in R. Figures were generated using Adobe Illustrator v2026(30.1).

#### Reporting summary

Further information on research design is available in the Nature Portfolio Reporting Summary linked to this article.

## Online content

Any methods, additional references, Nature Portfolio reporting summaries, source data, extended data, supplementary information, acknowledgements, peer review information; details of author contributions and competing interests; and statements of data and code availability are available at https://doi.org/10.1038/s41590-026-02637-w.

## Supplementary information

Supplementary Information Supplementary Tables 1–7.

Reporting Summary

Peer Review File

## Source data

Source Data Fig. 1 Statistical source data.

Source Data Fig. 2 Statistical source data.

Source Data Fig. 4 Statistical source data.

Source Data Fig. 5 Statistical source data.

Source Data Extended Data Fig. 1 Statistical source data.

Source Data Extended Data Fig. 2 Statistical source data.

Source Data Extended Data Fig. 3 Statistical source data.

Source Data Extended Data Fig. 4 Statistical source data.

Source Data Extended Data Fig. 5 Statistical source data.

Source Data Extended Data Fig. 6 Statistical source data.

Source Data Extended Data Fig. 7 Statistical source data.

Source Data Extended Data Fig. 8 Statistical source data.

Source Data Extended Data Fig. 9 Statistical source data.

## Data Availability

Owing to study participant confidentiality concerns, viral sequencing data cannot be publicly released, but will be made available to investigators upon reasonable request and after signing a data-sharing agreement. Access to individual-level data from the Multicenter AIDS Cohort Study (MACS)/Women’s Interagency HIV Study (WIHS) Combined Cohort Study (MWCCS) may be obtained upon review and approval of a MWCCS concept sheet (https://statepi.jhsph.edu/mwccs/work-with-us/). Source data are provided with the paper.
