# Supplementary material for: Innate immune imprints shape HIV-1 reservoir cell persistence during long-term antiretroviral therapy
Source: Nat Immunol. 2026 Sep 1;27(10):2029–42. doi: 10.1038/s41590-026-02637-w (PMC13630574; doi:10.1038/s41590-026-02637-w)
Supplement: Supplementary file 1 [file 41590_2026_2637_MOESM1_ESM.pdf]

nature immunology

Supplementary information

https://doi.org/10.1038/s41590-026-02637-w

Innate immune imprints shape HIV-1 reservoir cell persistence during long-term antiretroviral therapy

In the format provided by the authors and unedited

Supplementary Table 1: Clinical, immunogenetic and demographic characteristics of study participants on long-term ART.

	Total	BBw4+C2+	BBw4-C2+	BBw4+C2-	BBw4-C2-
**Number of participants**	104	56	13	18	17
**Age in years**	58.5 (34 – 82)	60.5 (43 – 76)	56 (34 – 73)	57.5 (43 – 82)	58 (48 – 81)
**Ethnicity (white %)**	61.54%	58.93%	46.15%	55.56%	88.24%
**Female (%)**	36.54%	39.29%	38.46%	38.89%	23.53%
**HLA-B*57/*27 (allelic %)**	9.13%	14.29%	0%	8.33%	0%
**HLA-B*35Px (allelic %)**	5.77%	9.82%	3.85%	0%	0%
**CD4 counts**	702 (294 – 1649)	685.5 (294 – 1452)	726 (461 – 1649)	597.5 (394 – 876)	735 (385 – 1110)
**Time since ART (years)**	21 (10 – 27)	21 (11 – 26)	23 (10 – 25)	22 (11 – 27)	19 (14 – 27)
**CMV seropositive status (+/-)**	102/2	55/1	13/0	18/0	16/1
**KIR2DL1 (presence/absence)**	103/1	56/0	13/0	17/1	17/0
**KIR2DL2 (presence/absence)**	47/57	21/35	5/8	9/9	12/5
**KIR2DL3 (presence/absence)**	99/5	54/2	12/1	17/1	16/1
**KIR3DL1 (presence/absence)**	102/2	55/1	13/0	18/0	16/1
**KIR3DL2 (presence/absence)**	104/0	56/0	13/0	18/0	17/0
**KIR3DL3 (presence/absence)**	104/0	56/0	13/0	18/0	17/0

Supplementary Table 2: Demographic characteristics of participants selected for immunophenotyping experiments.

	HIV-CMV+		HIV+CMV+		HIV-CMV-	
	**BBw4+C2+**	**BBw4-C2-**	**BBw4+C2+**	**BBw4-C2-**	**BBw4+C2+**	**BBw4-C2-**
**Number of participants**	12	10	30	12	15	14
**Age in years**	56.5 (34 – 76)	55.5 (30 – 68)	59.5 (43 – 75)	57.0 (48 – 81)	58 (32 – 74)	60.5 (34 – 73)
**Ethnicity (white %)**	75.0%	70%	56.67%	83.3%	100%	100%
**Female (%)**	33.33%	40.0%	26.67%	16.67%	53.33%	64.29%
**CD4 count**	NA	NA	634 (295 – 1452)	695 (385 – 925)	NA	NA
**Years on ART**	NA	NA	21.8 (12 – 26)	18.3 (15 – 27)	NA	NA

Supplementary Table 3: HLA class-1 alleles and classification of HLA-C alleles group in all participants described in Supplementary Table 2

Group	HLA-A_1	HLA-A_2	HLA-B_1	HLA-B_2	HLA-C_1	HLA-C_2	Residue 80 (allele 1/2)	Cgroups (allele1/2)
HIV-CMV+ BBw4+C2+ (n = 12)	A*11:01	A*11:01	B*27:02	B*55:01	C*02:02	C*03:03	Lys/Asn	C2/C1
	A*30:02	A*68:02	B*47:01	B*57:03	C*07:01	C*18:02	Asn/Lys	C1/C2
	A*02:01	A*32:01	B*44:02	B*47:01	C*05:01	C*06:02	Lys/Lys	C2/C2
	A*02:01	A*68:03	B*35:01	B*44:05	C*02:02	C*07:02	Lys/Asn	C2/C1
	A*02:01	A*02:01	B*35:01	B*44:02	C*01:02	C*05:01	Asn/Lys	C1/C2
	A*03:01	A*30:01	B*07:02	B*13:02	C*06:02	C*07:02	Lys/Asn	C2/C1
	A*02:01	A*11:01	B*44:02	B*58:01	C*05:01	C*12:03	Lys/Asn	C2/C1
	A*26:01	A*31:01	B*35:01	B*38:01	C*04:01	C*12:03	Lys/Asn	C2/C1
	A*31:01	A*66:01	B*35:01	B*53:01	C*04:01	C*04:01	Lys/Lys	C2/C2
	A*03:01	A*30:02	B*07:02	B*53:01	C*06:02	C*07:02	Lys/Asn	C2/C1
	A*01:01	A*29:02	B*44:03	B*57:01	C*06:02	C*16:01	Lys/Asn	C2/C1
	A*01:01	A*01:01	B*18:01	B*37:01	C*06:02	C*07:01	Lys/Asn	C2/C1
HIV-CMV+ BBw4-C2- (n = 10)	A*03:01	A*31:01	B*07:02	B*40:01	C*03:04	C*07:02	Asn/Asn	C1/C1
	A*02:01	A*03:01	B*07:02	B*39:05	C*07:02	C*07:02	Asn/Asn	C1/C1
	A*02:01	A*34:02	B*39:10	B*45:01	C*12:03	C*16:01	Asn/Asn	C1/C1
	A*01:01	A*32:01	B*08:01	B*40:01	C*03:04	C*07:01	Asn/Asn	C1/C1
	A*23:01	A*33:01	B*14:02	B*14:02	C*08:02	C*08:02	Asn/Asn	C1/C1
	A*29:10	A*32:01	B*14:02	B*55:01	C*03:03	C*08:02	Asn/Asn	C1/C1
	A*24:02	A*30:01	B*07:02	B*15:10	C*03:04	C*07:02	Asn/Asn	C1/C1
	A*01:01	A*03:01	B*07:02	B*07:02	C*07:02	C*07:02	Asn/Asn	C1/C1
	A*24:02	A*24:10	B*18:02	B*40:01	C*03:04	C*07:04	Asn/Asn	C1/C1
	A*03:01	A*03:01	B*08:01	B*14:02	C*07:02	C*08:02	Asn/Asn	C1/C1
HIV+CMV+ BBw4+C2+ (n = 30)	A*31:01	A*31:01	B*27:05	B*51:01	C*02:02	C*14:02	Lys/Asn	C2/C1
	A*02:01	A*29:02	B*13:02	B*44:03	C*06:02	C*16:01	Lys/Asn	C2/C1
	A*33:03	A*33:03	B*37:01	B*44:03	C*06:02	C*07:01	Lys/Asn	C2/C1
	A*29:02	A*68:02	B*53:01	B*58:01	C*04:01	C*07:01	Lys/Asn	C2/C1
	A*23:01	A*68:02	B*15:16	B*51:01	C*02:02	C*16:01	Lys/Asn	C2/C1
	A*01:01	A*02:01	B*44:02	B*57:01	C*06:02	C*12:03	Lys/Asn	C2/C1
	A*02:17	A*29:02	B*44:03	B*51:01	C*15:02	C*16:01	Lys/Asn	C2/C1
	A*01:01	A*34:02	B*35:01	B*37:01	C*06:02	C*06:02	Lys/Lys	C2/C2
	A*23:01	A*34:02	B*15:03	B*57:03	C*02:10	C*18:02	Lys/Lys	C2/C2
	A*02:01	A*30:02	B*27:05	B*35:17	C*02:02	C*04:01	Lys/Lys	C2/C2
	A*02:01	A*03:01	B*15:01	B*44:03	C*04:01	C*16:01	Lys/Asn	C2/C1
	A*11:01	A*24:02	B*07:02	B*44:02	C*05:01	C*07:02	Lys/Asn	C2/C1
	A*11:01	A*24:02	B*15:21	B*38:02	C*04:03	C*07:02	Lys/Asn	C2/C1
	A*01:01	A*03:01	B*07:02	B*57:01	C*06:02	C*07:02	Lys/Asn	C2/C1
	A*02:01	A*03:01	B*18:01	B*58:02	C*06:02	C*07:04	Lys/Asn	C2/C1

	A*23:01	A*25:01	B*18:01	B*58:02	C*06:02	C*12:03	Lys/Asn	C2/C1
	A*33:03	A*33:03	B*15:10	B*53:01	C*03:04	C*04:01	Asn/Lys	C1/C2
	A*01:01	A*30:01	B*08:01	B*13:02	C*06:02	C*07:01	Lys/Asn	C2/C1
	A*01:01	A*01:01	B*15:01	B*37:01	C*03:04	C*06:02	Asn/Lys	C1/C2
	A*02:01	A*03:01	B*14:02	B*44:02	C*05:01	C*08:02	Lys/Asn	C2/C1
	A*66:01	A*74:01	B*07:02	B*44:03	C*04:01	C*07:02	Lys/Asn	C2/C1
	A*03:01	A*36:01	B*35:02	B*57:03	C*04:01	C*07:01	Lys/Asn	C2/C1
	A*29:02	A*31:01	B*35:12	B*44:03	C*04:01	C*16:01	Lys/Asn	C2/C1
	A*23:01	A*29:02	B*15:01	B*53:01	C*03:03	C*04:01	Asn/Lys	C1/C2
	A*01:01	A*25:01	B*08:01	B*57:01	C*06:02	C*07:01	Lys/Asn	C2/C1
	A*02:01	A*02:01	B*07:02	B*44:02	C*05:01	C*07:02	Lys/Asn	C2/C1
	A*02:01	A*32:01	B*14:01	B*44:02	C*05:01	C*08:02	Lys/Asn	C2/C1
	A*30:02	A*66:01	B*15:10	B*57:03	C*03:04	C*18:02	Asn/Lys	C1/C2
	A*02:01	A*03:01	B*18:01	B*44:05	C*02:02	C*07:01	Lys/Asn	C2/C1
	A*02:01	A*03:01	B*15:01	B*44:02	C*03:04	C*05:01	Asn/Lys	C1/C2
**HIV+CMV+ BBw4-C2- (n = 12)**	A*02:01	A*25:06	B*18:01	B*18:01	C*07:01	C*12:03	Asn/Asn	C1/C1
	A*23:01	A*32:01	B*14:01	B*15:01	C*03:04	C*08:02	Asn/Asn	C1/C1
	A*02:01	A*31:01	B*14:02	B*40:01	C*03:04	C*08:02	Asn/Asn	C1/C1
	A*02:01	A*03:01	B*07:02	B*08:01	C*07:01	C*07:02	Asn/Asn	C1/C1
	A*03:01	A*24:02	B*07:02	B*39:06	C*07:02	C*07:02	Asn/Asn	C1/C1
	A*02:01	A*24:02	B*18:01	B*18:01	C*12:03	C*12:03	Asn/Asn	C1/C1
	A*29:01	A*32:01	B*07:02	B*40:01	C*03:04	C*07:02	Asn/Asn	C1/C1
	A*02:01	A*02:01	B*15:01	B*40:01	C*03:03	C*03:04	Asn/Asn	C1/C1
	A*11:01	A*68:01	B*15:01	B*18:03	C*03:03	C*07:01	Asn/Asn	C1/C1
	A*01:01	A*31:01	B*08:01	B*39:01	C*07:01	C*07:02	Asn/Asn	C1/C1
	A*02:02	A*30:01	B*07:02	B*35:01	C*07:02	C*07:05	Asn/Asn	C1/C1
	A*02:01	A*11:01	B*40:01	B*55:01	C*03:03	C*03:04	Asn/Asn	C1/C1
**HIV-CMV BBw4+C2+ (n =15)**	A*23:01	A*24:02	B*35:03	B*37:01	C*04:01	C*04:01	Lys/Lys	C2/C2
	A*24:02	A*26:01	B*38:01	B*57:01	C*06:02	C*12:03	Lys/Asn	C2/C1
	A*24:02	A*68:03	B*40:02	B*44:05	C*02:02	C*03:05	Lys/Asn	C2/C1
	A*03:01	A*03:01	B*07:02	B*37:01	C*06:02	C*07:02	Lys/Asn	C2/C1
	A*02:01	A*03:01	B*14:01	B*57:01	C*06:02	C*08:02	Lys/Asn	C2/C1
	A*01:01	A*68:01	B*08:01	B*44:03	C*04:01	C*07:01	Lys/Asn	C2/C1
	A*01:01	A*11:01	B*35:01	B*51:01	C*04:01	C*15:02	Lys/Lys	C2/C2
	A*01:01	A*24:02	B*44:03	B*57:01	C*06:02	C*16:01	Lys/Asn	C2/C1
	A*02:01	A*23:01	B*50:02	B*51:01	C*04:01	C*16:02	Lys/Lys	C2/C2
	A*02:01	A*24:02	B*15:01	B*44:02	C*03:03	C*05:01	Asn/Lys	C1/C2
	A*01:01	A*32:01	B*40:02	B*57:01	C*02:02	C*06:02	Lys/Lys	C2/C2
	A*02:01	A*11:01	B*35:01	B*37:01	C*04:01	C*06:02	Lys/Lys	C2/C2
	A*24:02	A*68:01	B*13:02	B*40:01	C*03:04	C*06:02	Asn/Lys	C1/C2
	A*03:01	A*30:01	B*56:01	B*57:01	C*01:02	C*06:02	Asn/Lys	C1/C2

	A*01:01	A*03:01	B*14:02	B*27:05	C*02:02	C*08:02	Lys/Asn	C2/C1
**HIV-CMV BBw4-C2- (n = 14)**	A*02:01	A*02:01	B*08:01	B*08:01	C*07:01	C*07:01	Asn/Asn	C1/C1
	A*01:01	A*01:01	B*08:01	B*08:01	C*07:01	C*07:01	Asn/Asn	C1/C1
	A*02:01	A*03:01	B*07:02	B*15:01	C*03:03	C*07:02	Asn/Asn	C1/C1
	A*01:01	A*01:01	B*08:01	B*08:01	C*07:01	C*07:01	Asn/Asn	C1/C1
	A*01:01	A*03:01	B*07:02	B*08:01	C*07:01	C*07:02	Asn/Asn	C1/C1
	A*02:01	A*03:01	B*07:02	B*55:01	C*03:03	C*07:02	Asn/Asn	C1/C1
	A*24:02	A*32:01	B*07:02	B*55:01	C*03:04	C*07:02	Asn/Asn	C1/C1
	A*02:01	A*26:08	B*15:01	B*39:01	C*03:04	C*12:03	Asn/Asn	C1/C1
	A*01:01	A*02:01	B*08:01	B*08:01	C*07:01	C*07:01	Asn/Asn	C1/C1
	A*01:01	A*31:01	B*08:01	B*40:01	C*03:04	C*07:01	Asn/Asn	C1/C1
	A*03:01	A*24:02	B*07:02	B*08:01	C*07:01	C*07:02	Asn/Asn	C1/C1
	A*01:01	A*02:01	B*07:02	B*56:01	C*01:02	C*07:02	Asn/Asn	C1/C1
	A*02:01	A*03:01	B*07:02	B*07:02	C*07:02	C*07:02	Asn/Asn	C1/C1
	A*02:01	A*32:01	B*07:02	B*15:01	C*03:03	C*07:02	Asn/Asn	C1/C1

Supplementary Table 4: Antibodies used for flow cytometric assessments of innate immune responses.

No of color	Marker	Clone	Fluorochrome	Dilution	Vendor	Ref No.
**1**	CD3	UCHT1	PerCP	1:100	Biolegend	300428
	CD19	H1B19		1:50	Biolegend	302228
	CD20	2H7		1:100	Biolegend	302324
**2**	CD14	M5E2	RB545	1:50	BD Biosciences	569259
**3**	CD16	3G8	BUV395	1:50	BD Biosciences	563784
**4**	CD56	MY31	RB744	1:25	BD Biosciences	757213
**5**	NKG2C (CD159c)	134591	BUV615	1:200	BD Biosciences	751059
**6**	NKp30 (CD337)	p30-15	BUV563	1:50	BD Biosciences	749129
**7**	NKp46 (CD335)	900	BV711	1:200	Biolegend	331936
**8**	NKG2D (CD314)	1D11	BV605	1:25	Biolegend	320832
**9**	DNAM-1 (CD226)	11A8	BV750	1:200	BD Biosciences	752656
**10**	CD161	DX12	BV650	1:33	BD Biosciences	563864
**11**	CD57	HNK-1	FITC	1:100	Biolegend	359604
**12**	NKG2A (CD159a)	S19004C	BV785	1:100	Biolegend	375138
**13**	CD69	FN50	Spark Red 718	1:100	Biolegend	310968
**14**	PD-1 (CD279)	A17188B	APC-Fire 810	1:50	Biolegend	621620
**15**	TIGIT	MBSA43	Alexa Fluor 660	1:50	Thermo Fisher Scientific	606-9500-41
**16**	CD158e1 (KIR3DL1)	DX9	BV421	1:50	Biolegend	312714
**17**	CD158b (KIR2DL2/DL3)	DX27	BB700	1:50	BD Biosciences - OptiBuild	746006
**18**	CD158a (KIR2DL1)	HP-DM1	PE	1:50	Biolegend	374904
**19**	CD158f (KIR2DL5)	UP-R1	Alexa Fluor 594	1:50	Novus Biologicals	NBP2-62222AF594
**20**	CD158d (KIR2DL4)	mAb 33 (33)	PE-Cy7	1:25	Thermo Fisher Scientific	MA5-38751
**21**	CD158k (KIR3DL2)	539304	APC-Cy7	1:100	Novus Biologicals	FAB2878APCCY7
**22**	LILRB1 (CD85j)	GHI/75	BUV805	1:33	BD Biosciences	748454
**23**	CD158i (KIR2DS4)	179315	Alexa Fluor 647	1:50	BD Biosciences	564375
**24**	KLRG1	SA231A2	PE-Fire 640	1:200	Biolegend	367742
**25**	MHC Class II (HLA-DR)	G46-6	BUV496	1:100	BD Biosciences	569674
**26**	CD11c	3.9	eFluor 450	1:100	Thermo Fisher Scientific	48-0116-41
**27**	CD123	S18016F	Spark UV 387	1:50	Biolegend	396726
**28**	CD64	10.1	BV480	1:50	BD Biosciences	566129
**29**	PD-L1 (CD274)	29E.2A3	cFluor-BYG710	1:50	Cytek Biosciences	R7-20613
**30**	Blue -fluorescence reactive dyes	NA	Live Dead Blue	1:1000	Invitrogen	L23105A

Supplementary Table 5. Antibodies used for flow cytometric assessments of antigen-specific T cell responses.

No of color	Marker	Clone	Fluorochrome	Dilution	Vendor	Ref No.
**1**	CD3	UCHT1	FITC	1:100	BD Biosciences	555332
**2**	CD4	RPA-T4	BUV395	1:100	BD Biosciences	564724
**3**	CD8a	RPA-T8	Pacific Blue	1:50	Biolegend	301033
**4**	IFN-gamma	4S.B3	BV785	1:33	Biolegend	502542
**5**	IL-2	MQ1-17H12	BV650	1:100	Biolegend	500334
**6**	TNF-alpha	MAb11	APC	1:50	Biolegend	502912
**7**	CD107a	H4A3	BV421	1:200	Biolegend	328626
	CD107b	H4B4		1:200	Biolegend	354308
**8**	Perforin	B-D48	PE-Cy7	1:100	Biolegend	353316
**9**	Granzyme A	CB9	Alexa Fluor 700	1:400	Biolegend	507210
**10**	Granzyme B	GB11	BV510	1:100	BD Biosciences	563388
**11**	Blue - fluorescence reactive dyes	NA	Live Dead Blue	1:1000	Invitrogen	L23105A

Supplementary Table 6: The area under the curve values for each peptide-specific stabilization assay in HLA-E transduced K562 (related to Figure 4d) or RMA-S (related to Extended Figure 8c) cell lines.

Peptide	K562-HLA-E	RMA-S-HLA-E
No Peptide	0	0
VL9-8I-positive control	650.1	273.4
NV9-negative control	61.66	45.49
KL9-WT	264.6	194.4
KL9-A6G variant	113.9	109.9
RE9-WT	28.29	25.86
RE9-E9A variant	13.02	7.382
SN9-WT	74.93	71.29
SN9-Y8F variant	82.46	84.97
RL9-WT	477.7	218.3
RL9-R1P variant	65.56	24.82

Supplementary Table 7: Primer pairs for mitochondrial DNA amplification in single-cell assays.

chr	Amplicon start	Amplicon end	Reference Genome	Forward primer (5' - 3')	Reverse primer (5' - 3')
chrM	14	263	hg38	TCACCCTATTAACCACTCACGG	TGGCTGTGCAGACATTCAATT
chrM	264	522	hg38	CTTTCCACACAGACATCATAACAAAA	GTGTGTGTGCTGGGTAGGAT
chrM	523	772	hg38	ACCGCTGCTAACCCCATAC	TTGCTGCGTGCTTGATGCT
chrM	773	1021	hg38	TGCAGCTCAAAACGCTTAGC	AGTCTATTTTGTGTCAACTGGAGTT
chrM	1022	1270	hg38	ACGAAAGTGGCTTTAACATATCTGA	AAGATGGCGGTATATAGGCTGA
chrM	1271	1466	hg38	CAGCAAACCCTGATGAAGGC	GGCCCTGTTCAACTAAGCAC
chrM	1467	1694	hg38	CTGAAGCGCGTACACACC	AGTGGGTTTGGGGCTAGGTT
chrM	1695	1934	hg38	CCACCTTACTACCAGACAACCT	AGCTGTTCTTAGGTAGCTCGT
chrM	1935	2184	hg38	AAAAGAGCACACCCGTCTATGT	TGCTTTTAGGCCTACTATGGGT
chrM	2185	2433	hg38	GCCACCAATTAAGAAAGCGTTC	GTGTTGGGTTGACAGTGAGG
chrM	2434	2644	hg38	AGGCATGCTCATAAGGAAAGGT	TCGTGGAGCCATTCATACAGG
chrM	2645	2856	hg38	GGGTTCAGCTGTCTCTTACTTTT	GCATGTACTGCTCGGAGGT
chrM	2860	3100	hg38	GACTTCACCAGTCAAAGCGAA	AGAAACCGACCTGGATTACTCC
chrM	3108	3321	hg38	TTCAAATTCCTCCCTGTACGAAAG	GTTGGCCATGGGTATGTTGT
chrM	3322	3547	hg38	CTCCTACTCCTCATTGTACCCA	TGGTGAGAGCTAAGGTCGG
chrM	3548	3797	hg38	TCGCTCTTCTACTATGAACCCC	GTGGAGAGGTTAAAGGAGCCA
chrM	3798	4002	hg38	CCTTATCACAACACAAGAACACCT	AATAATGTTTGTGTATTCGGCTATGAAG
chrM	4010	4259	hg38	ACACCCTCACCACTACAATCT	GTTTGAGGGGGAATGCTGG
chrM	4271	4521	hg38	GTCTGATAAAAGAGTTACTTTGATAGAGTAAAT	GTGTGCCTGCAAAGATGGT
chrM	4530	4798	hg38	GCGCTAAGCTCGCACTGA	GGGGCTATTCCTAGTTTTATTGCT
chrM	4800	4996	hg38	TTTCACTTCTGAGTCCCAGAGG	CGTAGCTGGGTTTGGTTTAATCC
chrM	5003	5253	hg38	CTTAGCATACTCCTCAATTACCCAC	GCAAAAAGCCGGTTAGCG
chrM	5259	5470	hg38	TGGGCCATTATCGAAGAATTCAC	GTGGTAAGGGCGATGAGTGT
chrM	5471	5721	hg38	GCTACTCCTACCTATCTCCCCT	AGCCAGTTGATTAGGGTGCT
chrM	5724	5982	hg38	AATCTACTTCTCCCGCCGC	CAGCTCATGCGCCGAATAA
chrM	5986	6247	hg38	TCCTAGGCACAGCTCTAAGC	GATGCGAGCAGGAGTAGGA
chrM	6248	6438	hg38	TGCTATAGTGGAGGCCGGA	ATTGGGTTATGGCAGGGGGT
chrM	6439	6649	hg38	ACCAAACGCCCCTCTTCG	GGTAGGATAAGAATATAAACTTCAGGGT
chrM	6650	6884	hg38	AGGCTTCGGAATAATCTCCCAT	GAGTGTGGCGAGTCAGCTA
chrM	6885	7134	hg38	CACGGAAGCAATATGAAATGATCTG	TGGCGTAGGTTTGGTCTAGG
chrM	7150	7399	hg38	TCATATTCATCGGCGTAAATCTAACT	GGGCATCCATATAGTCACTCCA
chrM	7410	7673	hg38	CACACATTCGAAGAACCCGT	TTATGAGGGCGTGATCATGAAAG
chrM	7675	7903	hg38	CATTTTCCTTATCTGCTTCCTAGTCC	TCAGTACCATTGGTGGCCA
chrM	7904	8129	hg38	ACCTACGAGTACACCGACTACG	TTTGGTTTAGACGTCCGGGA
chrM	8130	8371	hg38	CCACTTTCACCGCTACACG	GGGCATTTCACTGTAAAGAGGT

chrM	8372	8561	hg38	CAACTAAATACTACCGTATGGCCC	GGGGCAATGAATGAAGCGA
chrM	8563	8789	hg38	ACAATCCTAGGCCTACCCG	AGGAGTCCGAGGAGGTTAGT
chrM	8790	9037	hg38	GCCTCACTCATTTACACCAACC	TGAGTAGGTGGCCTGCAGTA
chrM	9038	9265	hg38	TGCACCTAATTGGAAGCGC	CCTGTTAGGGGTCATGGGCT
chrM	9267	9507	hg38	GCCCTCTCAGCCCTCCTAAT	AGGCTCAGAAAAATCCTGCG
chrM	9509	9758	hg38	TTACCACTCCAGCCTAGCC	AGACTCGAAGTACTCTGAGGC
chrM	9759	9950	hg38	CCCTTCACCATTTCCGACG	AACCACATCTACAAAATGCCAGT
chrM	9951	10197	hg38	TGACTATTTCTGTATGTCTCCATCTATTG	CGGGGGATATAGGGTCGAAG
chrM	10199	10453	hg38	CCGCGTCCCTTTCTCCATA	TCATAATTTAATGAGTCGAAATCATTCGT
chrM	10455	10679	hg38	AATCATATTTACCAAATGCCCCTCA	TTCGCAGGCGGCAAAGAC
chrM	10689	10920	hg38	GGCCTAGCCCTACTAGTCTCA	GGGGAACAGCTAAATAGGTTGTT
chrM	10921	11152	hg38	AACCTTTTCCTCCGACCCC	AGCCAAGGTGGGGATAAGTG
chrM	11154	11400	hg38	TCATCACCCGATGAGGCAA	AAGTGGAGTCCGTAAAGAGGT
chrM	11401	11639	hg38	ATGACTCCCTAAAGCCCATGT	TGTGGCTGATTGAAGAGTATGC
chrM	11641	11868	hg38	AGCCCTCGTAGTAACAGCC	GGTAAGGCGAGGTTAGCGA
chrM	11869	12117	hg38	CCCCACTATTAACCTACTGGGA	ATGTCGGGGTTGAGGGATAG
chrM	12118	12365	hg38	CATTACCGGGTTTTCCTCTTGT	AGGGTGGTTATAGTAGTGTGCA
chrM	12366	12557	hg38	AACCCTGACTTCCCTAATTCCC	GTTGTTTGGGTTGTGGCTCA
chrM	12558	12754	hg38	CCAGCTCTCCCTAAGCTTCA	GTTGGAATAGGTTGTTAGCGGT
chrM	12755	12969	hg38	TGTTCATCGGCTGAGAGGG	GGTGAGGCTTGGATTAGCGT
chrM	12970	13206	hg38	CCACTACTAGGCCTCCTCCT	GACTGCTGCGAACAGAGTG
chrM	13207	13453	hg38	TGCGCCCTTACACAAAATGAC	GGGAGGTTGAAGTGAGAGGTA
chrM	13454	13694	hg38	TCACCATTGGCAGCCTAGC	GGGTTTAGTAGGGTGGGGTT
chrM	13695	13940	hg38	CATTAAACGCCTGGCAGCC	CGGTGTGTGATGCTAGGGT
chrM	13941	14132	hg38	CACAATCCCCTATCTAGGCCT	AGGGTTAGGATGAGTGGGAAG
chrM	14133	14376	hg38	ACTCCTAATCACATAACCTATTCCCC	GTAGGATTGGTGCTGTGGGT
chrM	14377	14613	hg38	CTCCATCGCTAACCCCACT	TCTAAGCCTTCTCCTATTTATGGGG
chrM	14614	14860	hg38	AGAAAACCCCACAAACCCCAT	GCCAAGGAGTGAGCCGAA
chrM	14861	15108	hg38	GCCTGCCTGATCCTCCAAAT	AGCAGGAGGATAATGCCGAT
chrM	15109	15350	hg38	TGCAACTATAGCAACAGCCTTC	CGTGCAAGAATAGGAGGTGGA
chrM	15351	15592	hg38	AAACGGGATCAAACAACCCC	TCGGAGAATTGTGTAGGCGA
chrM	15593	15842	hg38	TCCGTCCCTAACAAACTAGGAG	TTAGGATTAGGATTGTTGTGAAGTATAGT
chrM	15843	16092	hg38	TACCAACTATCTCCCTAATTGAAAACA	ATACATAGCGGTTGTTGATGGG
chrM	16094	16340	hg38	TCGTACATTACTGCCAGCCA	TGTGCTATGTACGGTAAATGGC
chrM	16343	16567	hg38	ACAGTCAAATCCCTTCTCGTCC	TCGTGATGTCTTATTTAAGGGGAAC
