# Supplementary material for: Innate immune imprints shape HIV-1 reservoir cell persistence during long-term antiretroviral therapy
Source: Nat Immunol. 2026 Sep 1;27(10):2029–42. doi: 10.1038/s41590-026-02637-w (PMC13630574; doi:10.1038/s41590-026-02637-w)
Supplement: Supplementary file 2 [file 41590_2026_2637_MOESM2_ESM.pdf]

nature portfolio

Corresponding author(s): Mathias Lichterfeld

Last updated by author(s): 7/13/2026

Reporting Summary

Nature Portfolio wishes to improve the reproducibility of the work that we publish. This form provides structure for consistency and transparency in reporting. For further information on Nature Portfolio policies, see our Editorial Policies and the Editorial Policy Checklist.

Statistics

For all statistical analyses, confirm that the following items are present in the figure legend, table legend, main text, or Methods section.

n/a	Confirmed	
☐	☒	The exact sample size (*n*) for each experimental group/condition, given as a discrete number and unit of measurement
☐	☒	A statement on whether measurements were taken from distinct samples or whether the same sample was measured repeatedly
☐	☒	The statistical test(s) used AND whether they are one- or two-sided<br>*Only common tests should be described solely by name; describe more complex techniques in the Methods section.*
☒	☐	A description of all covariates tested
☐	☒	A description of any assumptions or corrections, such as tests of normality and adjustment for multiple comparisons
☐	☒	A full description of the statistical parameters including central tendency (e.g. means) or other basic estimates (e.g. regression coefficient) AND variation (e.g. standard deviation) or associated estimates of uncertainty (e.g. confidence intervals)
☐	☒	For null hypothesis testing, the test statistic (e.g. *F*, *t*, *r*) with confidence intervals, effect sizes, degrees of freedom and *P* value noted<br>*Give P values as exact values whenever suitable.*
☐	☒	For Bayesian analysis, information on the choice of priors and Markov chain Monte Carlo settings
☒	☐	For hierarchical and complex designs, identification of the appropriate level for tests and full reporting of outcomes
☐	☒	Estimates of effect sizes (e.g. Cohen's *d*, Pearson's *r*), indicating how they were calculated

Our web collection on statistics for biologists contains articles on many of the points above.

Software and code

Policy information about availability of computer code

Data collection: Tapestri (MissionBio, v2.0.1); SpectroFlo CS 1.2.0 (Cytek Biosciences)

Data analysis:
Graphpad prism (https://www.graphpad.com/scientific-software/prism/, version 8.2.1),
MEGA (https://www.megasoftware.net, version 11.0.13),
Adobe Illustrator (https://www.adobe.com/products/illustrator.html),
FlowJo (v10.10),
R (https://www.r-project.org, version 4.1),
SPICE (https://niaid.github.io/spice/),
Highlighter (https://www.hiv.lanl.gov/content/sequence/HIGHLIGHT/highlighter_top.html),
MAFFT (https://mafft.cbrc.jp/alignment/software, version 7),
MEGA (https://www.megasoftware.net, version 7.0.26),
MUSCLE (http://www.drive5.com/muscle, version 3.8.1551),
Bioconductor (https://bioconductor.org/, version 3.14),
Cutadapt (https://cutadapt.readthedocs.io/en/stable/, version 2.5),
Python (https://www.python.org, version 3.6),
bwa (https://github.com/bwa-mem2/bwa-mem2, version 2.2.1),
monocle3 (https://cole-trapnell-lab.github.io/monocle3/, version 1.0.0),
samtools and bcftools (http://www.htslib.org/, version 1.9),
MGATK (https://github.com/caleblareau/mgatk, version 0.6.1)
scMitoMut (https://github.com/wenjie1991/scMitoMut)

ggseqlogo (https://github.com/omarwagih/ggseqlogo)
COMPASS (https://www.bioconductor.org/packages/devel/bioc/html/COMPASS.html)

For manuscripts utilizing custom algorithms or software that are central to the research but not yet described in published literature, software must be made available to editors and reviewers. We strongly encourage code deposition in a community repository (e.g. GitHub). See the Nature Portfolio guidelines for submitting code & software for further information.

Data

Policy information about availability of data

All manuscripts must include a data availability statement. This statement should provide the following information, where applicable:

- Accession codes, unique identifiers, or web links for publicly available datasets
- A description of any restrictions on data availability
- For clinical datasets or third party data, please ensure that the statement adheres to our policy

Owing to study participant confidentiality concerns, viral sequencing data cannot be publicly released, but will be made available to investigators upon reasonable request and after signing a data sharing agreement. Correspondence and requests for materials should be addressed to Dr. Mathias Lichterfeld (mlichterfeld@partners.org). Access to individual-level data from the MACS/WIHS Combined Cohort Study (MWCCS) may be obtained upon review and approval of a MWCCS concept sheet (https://statepi.jhsph.edu/mwccs/work-with-us/).

Research involving human participants, their data, or biological material

Policy information about studies with human participants or human data. See also policy information about sex, gender (identity/presentation), and sexual orientation and race, ethnicity and racism.

Reporting on sex and gender	Sex and gender of participants are indicated in supplementary Table 1 and 2.
Reporting on race, ethnicity, or other socially relevant groupings	Race is indicated in supplementary Table 1 and 2.
Population characteristics	Demographic and clinical characteristics of study participants are reported in supplementary Table 1
Recruitment	Samples collected from individuals recruited into observational cohort studies at outpatient infectious disease clinics were used, as described in Methods sections of the manuscript. All participants provided informed consent.
Ethics oversight	Sample collection was approved by the respective institutional review boards.

Note that full information on the approval of the study protocol must also be provided in the manuscript.

Field-specific reporting

Please select the one below that is the best fit for your research. If you are not sure, read the appropriate sections before making your selection.

☒ Life sciences ☐ Behavioural & social sciences ☐ Ecological, evolutionary & environmental sciences

For a reference copy of the document with all sections, see nature.com/documents/nr-reporting-summary-flat.pdf

Life sciences study design

All studies must disclose on these points even when the disclosure is negative.

Sample size	A total of n=104 ART-treated patients were analyzed in data described in Figure 1-5. Sample sizes were based on specimen availability, reflecting the scarcity of the study population (people living with HIV who have been durably suppressed on antiretroviral therapy for more than 10 years).
Data exclusions	No data from the described individuals were excluded.
Replication	Replication of experiments was conducted when described in figure legends.
Randomization	No randomization was performed, because we performed a cross-sectional analysis of study participants enrolled in an observational study.
Blinding	Coded samples from study participants were used throughout the study; laboratory personnel was not blinded with regard to the respective study subjects, since this was a non-interventional, observational study. All sequencing reactions were performed at a local core facilities; core facility employees were fully blinded.

Reporting for specific materials, systems and methods

We require information from authors about some types of materials, experimental systems and methods used in many studies. Here, indicate whether each material, system or method listed is relevant to your study. If you are not sure if a list item applies to your research, read the appropriate section before selecting a response.

Materials & experimental systems

n/a	Involved in the study
☐	☒ Antibodies
☐	☒ Eukaryotic cell lines
☒	☐ Palaeontology and archaeology
☒	☐ Animals and other organisms
☒	☐ Clinical data
☒	☐ Dual use research of concern
☒	☐ Plants

Methods

n/a	Involved in the study
☒	☐ ChIP-seq
☐	☒ Flow cytometry
☒	☐ MRI-based neuroimaging

Antibodies

Antibodies used	All antibodies and their respective clone names, catalogue numbers, vendors and dilutions are provided in the methods section of the manuscript.
Validation	All antibodies were validated by the respective vendors.

Eukaryotic cell lines

Policy information about cell lines and Sex and Gender in Research

Cell line source(s)	HEK293T cell line was sourced from the Ragon Institute. HLA-E transfected K562 cell line (sex: female) and RMA-S mouse cell line (sex: unspecified) was a kind gift from M. Birnbaum (MIT, Cambridge, MA, USA).
Authentication	Phenotypic analysis by flow cytometry (e.g. staining of the protein product encoded by the transgene) and functional validation in assays.
Mycoplasma contamination	Mycoplasma contamination testing was performed per institutional guidelines of the Ragon Institute
Commonly misidentified lines (See ICLAC register)	*Name any commonly misidentified cell lines used in the study and provide a rationale for their use.*

Plants

Seed stocks	*Report on the source of all seed stocks or other plant material used. If applicable, state the seed stock centre and catalogue number. If plant specimens were collected from the field, describe the collection location, date and sampling procedures.*
Novel plant genotypes	*Describe the methods by which all novel plant genotypes were produced. This includes those generated by transgenic approaches, gene editing, chemical/radiation-based mutagenesis and hybridization. For transgenic lines, describe the transformation method, the number of independent lines analyzed and the generation upon which experiments were performed. For gene-edited lines, describe the editor used, the endogenous sequence targeted for editing, the targeting guide RNA sequence (if applicable) and how the editor was applied.*
Authentication	*Describe any authentication procedures for each seed stock used or novel genotype generated. Describe any experiments used to assess the effect of a mutation and, where applicable, how potential secondary effects (e.g. second site T-DNA insertions, mosiacism, off-target gene editing) were examined.*

Flow Cytometry

Plots

Confirm that:

☒ The axis labels state the marker and fluorochrome used (e.g. CD4-FITC).

☒ The axis scales are clearly visible. Include numbers along axes only for bottom left plot of group (a 'group' is an analysis of identical markers).

☒ All plots are contour plots with outliers or pseudocolor plots.

☒ A numerical value for number of cells or percentage (with statistics) is provided.

Methodology

Sample preparation	Sample preparation is described in the Methods section.

Instrument	Cytek Aurora (Aurora)
Software	SpectroFlo (Cytek Biosciences), FlowJo
Cell population abundance	No cell sorting was performed.
Gating strategy	Gating strategy was described in Extended Data Figure 10.

☒ Tick this box to confirm that a figure exemplifying the gating strategy is provided in the Supplementary Information.
