# Supplementary material for: Innate immune imprints shape HIV-1 reservoir cell persistence during long-term antiretroviral therapy
Source: Nat Immunol. 2026 Sep 1;27(10):2029–42. doi: 10.1038/s41590-026-02637-w (PMC13630574; doi:10.1038/s41590-026-02637-w)
Supplement: Supplementary file 3 [file 41590_2026_2637_MOESM3_ESM.pdf]

nature portfolio

Peer Review File

Innate immune imprints shape HIV-1 reservoir cell persistence during long-term antiretroviral therapy

Corresponding Author: Professor Mathias Lichterfeld

This file contains all editorial decision letters in order by version, followed by all author rebuttals in order by version.

Version 0:

Reviewer comments:

Reviewer #1

(Remarks to the Author)
In this study by Tan et al, the authors interrogated the impact of NK cells on HIV reservoir clearance, using the frequency of intact HIV provirus (versus defective HIV provirus) as a surrogate to measure the correlation between HLA-C2, HLA-E, and HLA-BBw4 recognition by NK inhibitory receptors KIR2DL, CD94/NKG2G, and KIR3DL1, respectively. The authors found that people having HLA-C2 and HLA-BBw4 have lower frequency of intact HIV proviruses, likely because of immune selection pressure, not initial infectivity (given that initial viral setpoint is not different). Using full-length HIV proviral sequencing and HLA typing, the authors found that HIV Vpu mutations correlate with lower frequency of intact HIV. The author continued and examined the E460A Gag variant (HLA-E presenting peptide) that correlates with the frequency of intact HIV. This is an innovative idea to explore how NK cells impose immune selection pressure on HIV, using HIV proviral sequence as a surrogate to identify footprints of NK selection pressure. The authors used a previously published method to examine NK cell proliferation by mitochondrial DNA sequence, which is innovative. Overall, this is an important and innovative study describing impact of NK cell on HIV reservoir. This study is already highly polished with no weaknesses found. I do not have suggestions for additional edits.

Reviewer #2

(Remarks to the Author)
A. Summary of the key results
Tan, Sun, Gao et al. present a tour-de-force analysis of proviral reservoir sequences, host immunogenetic, and antiviral immune responses in the setting of HIV infection that challenges the existing paradigm that the HIV reservoir is largely invisible to the immune system. In this fascinating manuscript, they instead suggest that NK cells, not T cells, play a role in recognizing reservoir cells, and that this recognition is through a combination of missing-self responses through KIR2DL1 and is aided by NKG2C-dependent recognition of HLA-E-restricted HIV-1 epitopes. In addition to challenging the paradigm of reservoir 'silence' this paper highlights a fundamental and previously unappreciated aspect of HIV biology—the fact that the immune responses important in untreated infection, where T cell responses are the most important, are quite distinct from those during treated infection when NK cells dominate. This paper therefore has important ramifications for our fundamental understanding of HIV biology during treatment and for our approach to cure strategies. This paper highlights the complex interplay between viral (particularly vpu) and host genetics and indeed opens the door for more personalized treatment strategies. Overall, I feel this paper is of great interest to the field. There are a few places where the conclusions could be strengthened as outlined below.

B. Originality and significance: if not novel, please include reference
This is a highly original and significant paper.
C. Data & methodology: validity of approach, quality of data, quality of presentation
Overall, the data and methodology are very clear with excellent presentation. However, the following major and minor comments would significantly improve the presentation and support the claims better.
Major comments
1. Linking claims to displayed data (Fig. 3b; Fig. 5f)
In Fig. 3b, the text links stronger clonal proliferation in P1 vs P2 to P1 having a lower reservoir, but reservoir measurements are not shown in that panel. Please add a cross-reference to where these reservoir metrics are displayed or temper the wording.

In Fig. 5f, since the main text emphasizes the C2-carrier interpretation, please briefly describe the pattern in C2 non-carriers and discuss why intact-provirus cells can show lower (or not higher) HLA-C than the broader HIV-DNA+ pool in some strata (biological or technical explanations).

2. C2-specificity as effect modification (Fig. 4a; optionally Fig. 5f)

Because a central claim is that variant–reservoir associations are preferentially observed in HLA-C2 carriers, it would strengthen the presentation to report a formal interaction/effect-modification test (variant × HLA-C2) or otherwise provide stratum-specific effect sizes (with CIs) to complement p-values.

3. Mechanistic inference around NKG2C/HLA-E and missing-self (Fig. 4d–e)

Fig. 4d/ED8 support generally weak HLA-E stabilization by HIV peptides and modest WT–variant differences for selected epitopes. Please consider reporting quantitative curve summaries (EC50/max/AUC) and use “surface stabilization” language consistently, reserving “affinity” for cases with direct binding evidence.

Fig. 4e/ED9 is a strong test of the missing-self + HLA-E/NKG2C model (with HLA-C downregulation and infection gating controls). To tighten causality, consider tempering language to “consistent with NKG2C involvement” unless receptor/ligand perturbation (e.g., NKG2C or HLA-E blockade) is available. Also, please cite ED9E (TNF-α, CD107a) in the main-text interpretation.

Minor comments

Extended Data Fig. 4i is descriptive within the KIR2DL1+KIR3DL1− gate; if possible, add an enrichment vs a reference NK subset (within-donor) or slightly temper the “adaptive NK” phrasing.

A. Extended comments (figure-linked, detailed)

Extended Figure 4i:

Extended Data Fig. 4i provides useful descriptive information on marker expression within the KIR2DL1+KIR3DL1− subset across strata. To better support the statement that these cells show “adaptive-like” features, it would be helpful to explicitly indicate whether these markers are enriched relative to an internal reference NK population (e.g., total CD56dim or another KIR-defined subset), ideally using a within-donor comparison or an enrichment metric. If additional canonical “adaptive” markers (e.g., CD62L and/or NKG2A) are available in the dataset, briefly referencing them alongside the current markers would strengthen the interpretation; otherwise, the text could be slightly tempered to indicate that the phenotype is ‘consistent with’ rather than definitive for adaptive NK cells.

Figure 3b:

The P1 vs P2 example is a clear illustration of mtDNA-defined NK clonality. However, the Results text links the stronger clonal proliferation in P1 to P1 having a lower reservoir (citing Fig. 3b), while Fig. 3b itself does not display reservoir measurements. Please either add a cross-reference to where the reservoir metric for these two participants is shown (main or supplement) or adjust the wording to keep Fig. 3b focused on clonality/phenotype without implying a reservoir difference that is not displayed in that figure.

Figure 4a:

Fig. 4a clearly presents variant–reservoir associations stratified by HLA-C2 status and supports the stated “C2-specific” pattern for several variants. To strengthen this claim statistically (and to help readers interpret whether apparent differences between strata reflect true effect modification), it would be helpful to report a formal interaction/effect-modification test (variant × HLA-C2) using an appropriate model for the reservoir outcome. If such modelling is not feasible, please state this explicitly and consider reporting effect sizes (with confidence intervals) within each stratum to complement the p-values.

Figure 4d:

Fig. 4d, together with Extended Data Fig. 8, supports the authors’ conclusion that HIV-derived HLA-E epitopes generally form relatively weak/low-stability HLA-E complexes compared with the VL9 control, and that WT–variant differences are modest and largely evident at higher peptide concentrations for RLVDGFLAL and KALGPAATL. To improve interpretability and cross-epitope comparison, please consider reporting quantitative curve summaries (e.g., EC50 and maximal stabilization or AUC) for WT vs variant peptides. Additionally, given that stabilization is an indirect readout influenced by peptide loading/exchange kinetics, it may be preferable to consistently describe the findings in terms of “HLA-E surface stabilization/steady-state expression,” while reserving “binding affinity” language for cases directly supported by the ELISA-based binding data (as shown for RLVDGFLAL). For epitopes with minimal stabilization differences, a short note clarifying whether this reflects assay sensitivity/kinetic limitations versus truly negligible effects would help align these in vitro results with the downstream functional assay in Fig. 4e.

Figure 4e:

Fig. 4e, together with Extended Data Fig. 9, provides a coherent experimental test of the “missing-self + HLA-E/NKG2C” model. The inclusion of Vpu constructs that do versus do not downregulate HLA-C, as well as the accompanying HLA-C downregulation measurements and comparable infection gating (p24+), are strengths and address key confounders.

1. To further tighten the causal interpretation that the peptide-variant effect is mediated specifically through NKG2C–HLA-E, it would be helpful to either (i) modestly temper the wording from “mediated by NKG2C” to “consistent with NKG2C involvement,” or (ii) include (if available) a receptor/ligand perturbation such as NKG2C or HLA-E blockade. While not strictly required given the subset-level specificity, such perturbation would strengthen the mechanistic claim.
2. Extended Data Fig. 9E includes additional functional readouts (TNF-α and CD107a), which is helpful. Please consider explicitly citing these data in the main-text interpretation of Fig. 4e so that the conclusion is not based solely on IFN-γ.
3. Because the functional assay uses “triple” Gag constructs, the experiment convincingly addresses the combined effect of multiple epitope variants but does not resolve whether one epitope dominates or whether effects are additive versus epistatic. Please briefly acknowledge this as an interpretive limitation when connecting the mechanistic assay to the individual-variant reservoir associations shown in Fig. 4a/b

Figure 5f:

Fig. 5f is helpful in stratifying HLA-C expression by C2 status and proviral category. Since the text focuses on the interpretation in C2 carriers, it would improve clarity to briefly describe the pattern in C2 non-carriers as well. Additionally,

because intact-provirus cells can show lower (or not higher) HLA-C relative to the broader HIV-DNA+ pool in some strata, a short discussion of potential biological and/or technical contributors (e.g., differences in infected-cell composition/activation state, history of viral gene expression, or classification/coverage bias) would be valuable.

D. Appropriate use of statistics and treatment of uncertainties
Except as noted above, statistics and uncertainties were treated well.
E. Conclusions: robustness, validity, reliability
Conclusions were supported except as noted above.
F. Suggested improvements: experiments, data for possible revision
Clarifications as noted above including new analyses. Blocking experiments could be consider but not necessary. Mostly analyses.
G. References: appropriate credit to previous work?
yes
H. Clarity and context: lucidity of abstract/summary, appropriateness of abstract, introduction and conclusions
Very clear

Reviewer #3

(Remarks to the Author)
Tan et al. Innate innate immune imprints shape HIV-1 reservoir cell persistence during long-term antiretroviral therapy

In this work the authors study NK cell biology in the context of reservoir decay under ART. Through an extensive series of experiments, the authors find that carriers of the HLA-C2 allele, along with expansion of HLA-E interacting NKG2C/KIR2DL1+ NK cells, are associated with reservoir decay of peripheral blood CD4+ T cells with intact HIV proviral DNA from donors on extended ART (cross-sectional cohort). HLA-E restricted peptides from HIV gag and Env are examined in detail for evidence of viral evolution or selection under ART. HIV vpu is shown to induce some degree of HLA-C downregulation, and vpu variants that differentially induce HLA-C downregulation are studied. The authors conclude that HIV reservoir decay occurring in HLA-C2+ donors on long term ART is associated with NK-cell mediated activity mediated through the KIR2DL1+NKG2C+ populations, wherein increased frequencies and activity of these cells is associated with decreased intact proviral DNA in the circulating CD4+ T cell reservoir.

I previously reviewed this manuscript for Nature, and note that this version has addressed a few of my concerns; however, the manuscript is largely unchanged in content despite some reorganization to match journal style. It appears that some data previously included as supplemental figures are now found in the main manuscript, and some figures have been reorganized. Some additional text and information have been added as well. However, most of my comments and concerns from the previous round were not addressed and therefore still stand.

The expanded discussion raises new points that are important advancements in the field. While I maintain my statement below regarding actionable directions, it is nevertheless important to consider that NK cells might have some influence on the reservoir during ART. The authors bring up that these data may revise our thoughts on the need for 'shock and kill' strategies, but I am not so convinced of this. To some degree, it depends on what one considers the 'latent' reservoir, a definition that varies depending on whom you ask. If one considers a full-length provirus integrated into heterochromatic DNA, with itself packaged into heterochromatin matching the background region such that no viral transcripts of any sort are produced, one may need a shock to make this virus visible to the immune system.

The new paragraph (lines 427-453) is a very nice encapsulation of how T cells and NK cells could work together at different phases after ART initiation to impose differential pressure on the evolving state of the HIV reservoir. The points raised reinforce my previous comment that this study needs longitudinal, rather than cross-sectional, assessments. I appreciate that this is extremely difficult given the need for long term follow up and availability of samples. This may not be achievable—but at the very least it should be addressed.

General comments:
1. It is unclear how these findings provide actionable directions for the development of HIV cure strategies. While interesting from the pathogenesis and immunity standpoint, it is not immediately apparent that these studies will have a broad impact on the HIV cure field. What are the authors proposing to make these findings clinically valuable?
2. The data shown in the manuscript, while technically well performed and extensive, demonstrate associations, and not mechanisms, of potential control mediated by NK cells. All cohort based studies are cross-sectional in nature, yet interpret a longitudinal effect on the reservoir. No association is shown demonstrating time on ART with reservoir level, where one would expect a progressive decay with longer time on ART. Notably, it is expected that intact proviral DNA will decay during extended ART as has been shown by others. Is this accelerated in individuals with HLA-C2?
3. It is not clear that the statistical differences observed in many of the analyses are biologically meaningful. The frequency of intact proviral DNA, for example, appears quite similar between the cohorts in many instances. Are these differences meaningful? Would one expect viral rebound to be delayed in HLA-C2+ individuals due to the slightly lower reservoir? Lessons from non-human primates and other human studies suggest that circulating reservoir frequencies do not translate well to rebound time (unless the reservoir is extremely low). Likewise, it is unclear that the frequencies of the different NK cell functions between cohorts are biologically impactful. Is there evidence at a change in peripheral blood NK cell subset

frequency of a few percent can have such a potential effect longitudinally on the HIV reservoir? Do these populations exhibit activity in tissues where the reservoir resides? The HLA-C 'down-regulation' observed with vpu appears minimal (1-2 tenths of a log, statistical significance unknown). Is this sufficient to alter the recognition of HLA-C restricted epitopes by T cells or prevent NK cell recognition through NKG2C?
4. The cross-sectional design of the study reduces the strength of the results. The optimal study here would be to show preferential decay of intact proviral reservoirs over time within HLA-C2+ vs HLA-C2- donors on long term ART. Within these donors there would also need to be extensive analysis to rule out potential effects of other immune mechanisms, specifically T cells (see point 5).
5. Analysis of adaptive responses, especially T cells, is insufficient to rule out a potential effect of T cell mediated immunity on reservoir decay, as only responses to Gag were measured.
6. The expansion of NKG2C+ NK cells, as stated by the authors, is known to be associated with CMV. CMV is also associated with increased KIR2DL1+ NK cells. While the authors state that "CMV infection did not seem to represent a sufficient factor", I feel that CMV is a major driver of the effect given the well-documented effect on these subsets of NK cells after CMV acquisition.
7. Do the authors feel that there is evidence of vpu sequence evolution taking place, or vpu sequence selection? Presuming under long term fully suppressive ART, depending on who we believe in the literature, there should not be ongoing rounds of viral replication. As such, how would vpu be evolving under innate immune pressure? Or is this selection occurring within the reservoir? Here, it would be important to understand the variation of this region of vpu prior to ART initiation when the reservoir presumably forms. I understand the complexity of this question, but this issue is central to interpretation of the entire manuscript, and needs to be carefully considered.

Technical comments.
1. The mitoseq and phepseq studies, while interesting and technically demanding, provide insufficient support for their respective conclusions due to the low n (2 per cohort) studied. The value of these analyses to the overall conclusion is thus limited.
2. The sequence analysis of vpu in Figure 5A appears to show an insert at position 7 relative to the consensus clade B vpu sequence available on the LANL database. If the sequences are correct as shown, and an insertion is truly present, there is concern whether vpu function in these donors is altered given that this area has multiple transmembrane regions.

Decision Letter:

27th May 2026

Dear Dr Lichterfeld,

Your Article, "Innate immune imprints shape HIV-1 reservoir cell persistence during long-term antiretroviral therapy" has now been seen by 3 referees. You will see from their comments below that while they find your work of interest, some important points are raised. We are very interested in the possibility of publishing your study in Nature Immunology, but would like to consider your response to these concerns in the form of a revised manuscript before we make a final decision on publication.

As you will see Reviewer #6 would like you to do further analyses to strengthen the conclusions. Please address the issues raised by Reviewer #6 and the remaining issues from Reviewer #4 where possible. After we receive the revised version of the manuscript, it will be sent back to Reviewer #6 only.

We therefore invite you to revise your manuscript taking into account all reviewer and editor comments. Please highlight all changes in the manuscript text file in Microsoft Word format.

We are committed to providing a fair and constructive peer-review process. Do not hesitate to contact us if there are specific requests from the reviewers that you believe are technically impossible or unlikely to yield a meaningful outcome.

When revising your manuscript:

* Include a "Response to referees" document detailing, point-by-point, how you addressed each referee comment. If no action was taken to address a point, you must provide a compelling argument. This response will be sent back to the referees along with the revised manuscript.

* If you have not done so already please begin to revise your manuscript so that it conforms to our Article format instructions at http://www.nature.com/ni/authors/index.html. Refer also to any guidelines provided in this letter.

* Please include a revised version of any required reporting checklist. It will be available to referees to aid in their evaluation of the manuscript goes back for peer review. They are available here:

Reporting summary:
https://www.nature.com/documents/nr-reporting-summary.pdf

When submitting the revised version of your manuscript, please pay close attention to our

href="https://www.nature.com/nature-portfolio/editorial-policies/image-integrity">Digital Image Integrity Guidelines. and to the following points below:

-- that unprocessed scans are clearly labelled and match the gels and western blots presented in figures.
-- that control panels for gels and western blots are appropriately described as loading on sample processing controls
-- all images in the paper are checked for duplication of panels and for splicing of gel lanes.

Please note, Extended Data figures and tables are online-only (appearing in the online PDF and full-text HTML version of the paper), peer-reviewed display items that provide essential background to the Article but are not included in the printed version of the paper due to space constraints or being of interest only to a few specialists. A maximum of ten Extended Data display items (figures and tables) is typically permitted. When re-submitting your manuscript, please ensure that any supplementary figures and tables that are more critical to the manuscript's conclusions are converted to Extended data to increase these data's visibility.

Finally, please ensure that you retain unprocessed data and metadata files after publication, ideally archiving data in perpetuity, as these may be requested during the peer review and production process or after publication if any issues arise.

Please use the link below to submit your revised manuscript and related files:

Link Redacted

Note: This URL links to your confidential home page and associated information about manuscripts you may have submitted, or that you are reviewing for us. If you wish to forward this email to co-authors, please delete the link to your homepage.

We hope to receive your revised manuscript within two weeks. If you cannot send it within this time, please let us know. We will be happy to consider your revision so long as nothing similar has been accepted for publication at Nature Immunology or published elsewhere.

Please do not hesitate to contact me if you have any questions or would like to discuss these revisions further.

Nature Immunology is committed to improving transparency in authorship. As part of our efforts in this direction, we are now requesting that all authors identified as 'corresponding author' on published papers create and link their Open Researcher and Contributor Identifier (ORCID) with their account on the Manuscript Tracking System (MTS), prior to acceptance. ORCID helps the scientific community achieve unambiguous attribution of all scholarly contributions. You can create and link your ORCID from the home page of the MTS by clicking on 'Modify my Springer Nature account'. For more information please visit please visit www.springernature.com/orcid.

We look forward to seeing the revised manuscript and thank you for the opportunity to review your work.

Sincerely,
Paula

Paula Jauregui, PhD
Senior Editor
Nature Immunology

Referee expertise:

Referee #1: Spatial transcriptomics, HIV (Previous reviewer #1 at Nature)

Referee #4: HIV immunopathogenesis, T cells (Previous reviewer #4 at Nature)

Referee #6: HIV pathogenesis and immunology (Substitute for reviewer #5 at Nature that could not re-review)

Reviewers' Comments:

Reviewer #1 (Remarks to the Author):

In this study by Tan et al, the authors interrogated the impact of NK cells on HIV reservoir clearance, using the frequency of intact HIV provirus (versus defective HIV provirus) as a surrogate to measure the correlation between HLA-C2, HLA-E, and HLA-BBw4 recognition by NK inhibitory receptors KIR2DL, CD94/NKG2G, and KIR3DL1, respectively. The authors found that people having HLA-C2 and HLA-BBw4 have lower frequency of intact HIV proviruses, likely because of immune selection pressure, not initial infectivity (given that initial viral setpoint is not different). Using full-length HIV proviral sequencing and HLA typing, the authors found that HIV Vpu mutations correlate with lower frequency of intact HIV. The

author continued and examined the E460A Gag variant (HLA-E presenting peptide) that correlates with the frequency of intact HIV. This is an innovative idea to explore how NK cells impose immune selection pressure on HIV, using HIV proviral sequence as a surrogate to identify footprints of NK selection pressure. The authors used a previously published method to examine NK cell proliferation by mitochondrial DNA sequence, which is innovative. Overall, this is an important and innovative study describing impact of NK cell on HIV reservoir. This study is already highly polished with no weaknesses found. I do not have suggestions for additional edits.

Reviewer #4 (Remarks to the Author):

Tan et al. Innate innate immune imprints shape HIV-1 reservoir cell persistence during long-term antiretroviral therapy

In this work the authors study NK cell biology in the context of reservoir decay under ART. Through an extensive series of experiments, the authors find that carriers of the HLA-C2 allele, along with expansion of HLA-E interacting NKG2C/KIR2DL1+ NK cells, are associated with reservoir decay of peripheral blood CD4+ T cells with intact HIV proviral DNA from donors on extended ART (cross-sectional cohort). HLA-E restricted peptides from HIV gag and Env are examined in detail for evidence of viral evolution or selection under ART. HIV vpu is shown to induce some degree of HLA-C downregulation, and vpu variants that differentially induce HLA-C downregulation are studied. The authors conclude that HIV reservoir decay occurring in HLA-C2+ donors on long term ART is associated with NK-cell mediated activity mediated through the KIR2DL1+NKG2C+ populations, wherein increased frequencies and activity of these cells is associated with decreased intact proviral DNA in the circulating CD4+ T cell reservoir.

I previously reviewed this manuscript for Nature, and note that this version has addressed a few of my concerns; however, the manuscript is largely unchanged in content despite some reorganization to match journal style. It appears that some data previously included as supplemental figures are now found in the main manuscript, and some figures have been reorganized. Some additional text and information have been added as well. However, most of my comments and concerns from the previous round were not addressed and therefore still stand.

The expanded discussion raises new points that are important advancements in the field. While I maintain my statement below regarding actionable directions, it is nevertheless important to consider that NK cells might have some influence on the reservoir during ART. The authors bring up that these data may revise our thoughts on the need for ‘shock and kill’ strategies, but I am not so convinced of this. To some degree, it depends on what one considers the ‘latent’ reservoir, a definition that varies depending on whom you ask. If one considers a full-length provirus integrated into heterochromatic DNA, with itself packaged into heterochromatin matching the background region such that no viral transcripts of any sort are produced, one may need a shock to make this virus visible to the immune system.

The new paragraph (lines 427-453) is a very nice encapsulation of how T cells and NK cells could work together at different phases after ART initiation to impose differential pressure on the evolving state of the HIV reservoir. The points raised reinforce my previous comment that this study needs longitudinal, rather than cross-sectional, assessments. I appreciate that this is extremely difficult given the need for long term follow up and availability of samples. This may not be achievable—but at the very least it should be addressed.

General comments:
1. It is unclear how these findings provide actionable directions for the development of HIV cure strategies. While interesting from the pathogenesis and immunity standpoint, it is not immediately apparent that these studies will have a broad impact on the HIV cure field. What are the authors proposing to make these findings clinically valuable?
2. The data shown in the manuscript, while technically well performed and extensive, demonstrate associations, and not mechanisms, of potential control mediated by NK cells. All cohort based studies are cross-sectional in nature, yet interpret a longitudinal effect on the reservoir. No association is shown demonstrating time on ART with reservoir level, where one would expect a progressive decay with longer time on ART. Notably, it is expected that intact proviral DNA will decay during extended ART as has been shown by others. Is this accelerated in individuals with HLA-C2?
3. It is not clear that the statistical differences observed in many of the analyses are biologically meaningful. The frequency of intact proviral DNA, for example, appears quite similar between the cohorts in many instances. Are these differences meaningful? Would one expect viral rebound to be delayed in HLA-C2+ individuals due to the slightly lower reservoir? Lessons from non-human primates and other human studies suggest that circulating reservoir frequencies do not translate well to rebound time (unless the reservoir is extremely low). Likewise, it is unclear that the frequencies of the different NK cell functions between cohorts are biologically impactful. Is there evidence at a change in peripheral blood NK cell subset frequency of a few percent can have such a potential effect longitudinally on the HIV reservoir? Do these populations exhibit activity in tissues where the reservoir resides? The HLA-C ‘down-regulation’ observed with vpu appears minimal (1-2 tenths of a log, statistical significance unknown). Is this sufficient to alter the recognition of HLA-C restricted epitopes by T cells or prevent NK cell recognition through NKG2C?
4. The cross-sectional design of the study reduces the strength of the results. The optimal study here would be to show preferential decay of intact proviral reservoirs over time within HLA-C2+ vs HLA-C2- donors on long term ART. Within these donors there would also need to be extensive analysis to rule out potential effects of other immune mechanisms, specifically T cells (see point 5).
5. Analysis of adaptive responses, especially T cells, is insufficient to rule out a potential effect of T cell mediated immunity on reservoir decay, as only responses to Gag were measured.
6. The expansion of NKG2C+ NK cells, as stated by the authors, is known to be associated with CMV. CMV is also

associated with increased KIR2DL1+ NK cells. While the authors state that “CMV infection did not seem to represent a sufficient factor”, I feel that CMV is a major driver of the effect given the well-documented effect on these subsets of NK cells after CMV acquisition.
7. Do the authors feel that there is evidence of vpu sequence evolution taking place, or vpu sequence selection? Presuming under long term fully suppressive ART, depending on who we believe in the literature, there should not be ongoing rounds of viral replication. As such, how would vpu be evolving under innate immune pressure? Or is this selection occurring within the reservoir? Here, it would be important to understand the variation of this region of vpu prior to ART initiation when the reservoir presumably forms. I understand the complexity of this question, but this issue is central to interpretation of the entire manuscript, and needs to be carefully considered.

Technical comments.
1. The mitoseq and phepseq studies, while interesting and technically demanding, provide insufficient support for their respective conclusions due to the low n (2 per cohort) studied. The value of these analyses to the overall conclusion is thus limited.
2. The sequence analysis of vpu in Figure 5A appears to show an insert at position 7 relative to the consensus clade B vpu sequence available on the LANL database. If the sequences are correct as shown, and an insertion is truly present, there is concern whether vpu function in these donors is altered given that this area has multiple transmembrane regions.

Reviewer #2 (Remarks to the Author):

A. Summary of the key results
Tan, Sun, Gao et al. present a tour-de-force analysis of proviral reservoir sequences, host immunogenetic, and antiviral immune responses in the setting of HIV infection that challenges the existing paradigm that the HIV reservoir is largely invisible to the immune system. In this fascinating manuscript, they instead suggest that NK cells, not T cells, play a role in recognizing reservoir cells, and that this recognition is through a combination of missing-self responses through KIR2DL1 and is aided by NKG2C-dependent recognition of HLA-E-restricted HIV-1 epitopes. In addition to challenging the paradigm of reservoir ‘silence’ this paper highlights a fundamental and previously unappreciated aspect of HIV biology—the fact that the immune responses important in untreated infection, where T cell responses are the most important, are quite distinct from those during treated infection when NK cells dominate. This paper therefore has important ramifications for our fundamental understanding of HIV biology during treatment and for our approach to cure strategies. This paper highlights the complex interplay between viral (particularly vpu) and host genetics and indeed opens the door for more personalized treatment strategies. Overall, I feel this paper is of great interest to the field. There are a few places where the conclusions could be strengthened as outlined below.

B. Originality and significance: if not novel, please include reference
This is a highly original and significant paper.
C. Data & methodology: validity of approach, quality of data, quality of presentation
Overall, the data and methodology are very clear with excellent presentation. However, the following major and minor comments would significantly improve the presentation and support the claims better.
Major comments
1. Linking claims to displayed data (Fig. 3b; Fig. 5f)
In Fig. 3b, the text links stronger clonal proliferation in P1 vs P2 to P1 having a lower reservoir, but reservoir measurements are not shown in that panel. Please add a cross-reference to where these reservoir metrics are displayed or temper the wording.
In Fig. 5f, since the main text emphasizes the C2-carrier interpretation, please briefly describe the pattern in C2 non-carriers and discuss why intact-provirus cells can show lower (or not higher) HLA-C than the broader HIV-DNA+ pool in some strata (biological or technical explanations).
2. C2-specificity as effect modification (Fig. 4a; optionally Fig. 5f)
Because a central claim is that variant–reservoir associations are preferentially observed in HLA-C2 carriers, it would strengthen the presentation to report a formal interaction/effect-modification test (variant × HLA-C2) or otherwise provide stratum-specific effect sizes (with CIs) to complement p-values.
3. Mechanistic inference around NKG2C/HLA-E and missing-self (Fig. 4d–e)
Fig. 4d/ED8 support generally weak HLA-E stabilization by HIV peptides and modest WT–variant differences for selected epitopes. Please consider reporting quantitative curve summaries (EC50/max/AUC) and use “surface stabilization” language consistently, reserving “affinity” for cases with direct binding evidence.
Fig. 4e/ED9 is a strong test of the missing-self + HLA-E/NKG2C model (with HLA-C downregulation and infection gating controls). To tighten causality, consider tempering language to “consistent with NKG2C involvement” unless receptor/ligand perturbation (e.g., NKG2C or HLA-E blockade) is available. Also, please cite ED9E (TNF-α, CD107a) in the main-text interpretation.

Minor comments
Extended Data Fig. 4i is descriptive within the KIR2DL1+KIR3DL1– gate; if possible, add an enrichment vs a reference NK subset (within-donor) or slightly temper the “adaptive NK” phrasing.

A. Extended comments (figure-linked, detailed)
Extended Figure 4i:

Extended Data Fig. 4i provides useful descriptive information on marker expression within the KIR2DL1+KIR3DL1− subset across strata. To better support the statement that these cells show "adaptive-like" features, it would be helpful to explicitly indicate whether these markers are enriched relative to an internal reference NK population (e.g., total CD56dim or another KIR-defined subset), ideally using a within-donor comparison or an enrichment metric. If additional canonical "adaptive" markers (e.g., CD62L and/or NKG2A) are available in the dataset, briefly referencing them alongside the current markers would strengthen the interpretation; otherwise, the text could be slightly tempered to indicate that the phenotype is 'consistent with' rather than definitive for adaptive NK cells.
Figure 3b:
The P1 vs P2 example is a clear illustration of mtDNA-defined NK clonality. However, the Results text links the stronger clonal proliferation in P1 to P1 having a lower reservoir (citing Fig. 3b), while Fig. 3b itself does not display reservoir measurements. Please either add a cross-reference to where the reservoir metric for these two participants is shown (main or supplement) or adjust the wording to keep Fig. 3b focused on clonality/phenotype without implying a reservoir difference that is not displayed in that figure.
Figure 4a:
Fig. 4a clearly presents variant–reservoir associations stratified by HLA-C2 status and supports the stated "C2-specific" pattern for several variants. To strengthen this claim statistically (and to help readers interpret whether apparent differences between strata reflect true effect modification), it would be helpful to report a formal interaction/effect-modification test (variant × HLA-C2) using an appropriate model for the reservoir outcome. If such modelling is not feasible, please state this explicitly and consider reporting effect sizes (with confidence intervals) within each stratum to complement the p-values.
Figure 4d:
Fig. 4d, together with Extended Data Fig. 8, supports the authors' conclusion that HIV-derived HLA-E epitopes generally form relatively weak/low-stability HLA-E complexes compared with the VL9 control, and that WT–variant differences are modest and largely evident at higher peptide concentrations for RLVDGFLAL and KALGPAATL. To improve interpretability and cross-epitope comparison, please consider reporting quantitative curve summaries (e.g., EC50 and maximal stabilization or AUC) for WT vs variant peptides. Additionally, given that stabilization is an indirect readout influenced by peptide loading/exchange kinetics, it may be preferable to consistently describe the findings in terms of "HLA-E surface stabilization/steady-state expression," while reserving "binding affinity" language for cases directly supported by the ELISA-based binding data (as shown for RLVDGFLAL). For epitopes with minimal stabilization differences, a short note clarifying whether this reflects assay sensitivity/kinetic limitations versus truly negligible effects would help align these in vitro results with the downstream functional assay in Fig. 4e.
Figure 4e:
Fig. 4e, together with Extended Data Fig. 9, provides a coherent experimental test of the "missing-self + HLA-E/NKG2C" model. The inclusion of Vpu constructs that do versus do not downregulate HLA-C, as well as the accompanying HLA-C downregulation measurements and comparable infection gating (p24+), are strengths and address key confounders.
1. To further tighten the causal interpretation that the peptide-variant effect is mediated specifically through NKG2C–HLA-E, it would be helpful to either (i) modestly temper the wording from "mediated by NKG2C" to "consistent with NKG2C involvement," or (ii) include (if available) a receptor/ligand perturbation such as NKG2C or HLA-E blockade. While not strictly required given the subset-level specificity, such perturbation would strengthen the mechanistic claim.
2. Extended Data Fig. 9E includes additional functional readouts (TNF-α and CD107a), which is helpful. Please consider explicitly citing these data in the main-text interpretation of Fig. 4e so that the conclusion is not based solely on IFN-γ.
3. Because the functional assay uses "triple" Gag constructs, the experiment convincingly addresses the combined effect of multiple epitope variants but does not resolve whether one epitope dominates or whether effects are additive versus epistatic. Please briefly acknowledge this as an interpretive limitation when connecting the mechanistic assay to the individual-variant reservoir associations shown in Fig. 4a/b
Figure 5f:
Fig. 5f is helpful in stratifying HLA-C expression by C2 status and proviral category. Since the text focuses on the interpretation in C2 carriers, it would improve clarity to briefly describe the pattern in C2 non-carriers as well. Additionally, because intact-provirus cells can show lower (or not higher) HLA-C relative to the broader HIV-DNA+ pool in some strata, a short discussion of potential biological and/or technical contributors (e.g., differences in infected-cell composition/activation state, history of viral gene expression, or classification/coverage bias) would be valuable.

D. Appropriate use of statistics and treatment of uncertainties
Except as noted above, statistics and uncertainties were treated well.
E. Conclusions: robustness, validity, reliability
Conclusions were supported except as noted above.
F. Suggested improvements: experiments, data for possible revision
Clarifications as noted above including new analyses. Blocking experiments could be consider but not necessary. Mostly analyses.
G. References: appropriate credit to previous work?
yes
H. Clarity and context: lucidity of abstract/summary, appropriateness of abstract, introduction and conclusions
Very clear

Version 1:

Reviewer comments:

Reviewer #2

(Remarks to the Author)
In this revised manuscript, the authors have fully addressed my prior concerns. The new analyses added have further strengthened the manuscript, which I find to be impactful and important for the field. I respectfully disagree with the co-reviewer's premise that a longitudinal study is required (as that would be an entirely different study and such samples are incredibly difficult to acquire) and that this study is of no value if it does not provide immediate actionable directions for HIV cure strategies. This manuscript describes a complex interaction between KIR2DL1 and NKG2C on NK cells in response to the HIV reservoir which is adding new knowledge, and is therefore important.

Decision Letter:

Our ref: NI-A43024A

22nd Jun 2026

Dear Dr. Lichterfeld,

Thank you for submitting your revised manuscript "Innate immune imprints shape HIV-1 reservoir cell persistence during long-term antiretroviral therapy" (NI-A43024A). It has now been seen by the original referees and their comments are below. The reviewers find that the paper has improved in revision, and therefore we'll be happy in principle to publish it in Nature Immunology, pending minor revisions to satisfy the referees' final requests and to comply with our editorial and formatting guidelines.

We will now perform detailed checks on your paper and will send you a checklist detailing our editorial and formatting requirements in a couple of weeks. Please do not upload the final materials and make any revisions until you receive this additional information from us.

If you had not uploaded a Word file for the current version of the manuscript, we will need one before beginning the editing process; please email that to immunology@us.nature.com at your earliest convenience.

Thank you again for your interest in Nature Immunology Please do not hesitate to contact me if you have any questions.

Sincerely,
Paula

Paula Jauregui, PhD
Senior Editor
Nature Immunology

Reviewer #2 (Remarks to the Author):

In this revised manuscript, the authors have fully addressed my prior concerns. The new analyses added have further strengthened the manuscript, which I find to be impactful and important for the field. I respectfully disagree with the co-reviewer's premise that a longitudinal study is required (as that would be an entirely different study and such samples are incredibly difficult to acquire) and that this study is of no value if it does not provide immediate actionable directions for HIV cure strategies. This manuscript describes a complex interaction between KIR2DL1 and NKG2C on NK cells in response to the HIV reservoir which is adding new knowledge, and is therefore important.

INTERNAL USE ONLY AIP modules required: ["Life Science"]

Open Access This Peer Review File is licensed under a Creative Commons Attribution 4.0 International License, which permits use, sharing, adaptation, distribution and reproduction in any medium or format, as long as you give appropriate credit to the original author(s) and the source, provide a link to the Creative Commons license, and indicate if changes were made.
In cases where reviewers are anonymous, credit should be given to 'Anonymous Referee' and the source.
The images or other third party material in this Peer Review File are included in the article's Creative Commons license, unless indicated otherwise in a credit line to the material. If material is not included in the article's Creative Commons license and your intended use is not permitted by statutory regulation or exceeds the permitted use, you will need to obtain permission directly from the copyright holder.
To view a copy of this license, visit https://creativecommons.org/licenses/by/4.0/

Response to Reviewers: We sincerely thank all three reviewers for their thoughtful evaluation of our manuscript and for their insightful comments and suggestions. In response, we have incorporated additional experimental data, performed further analyses, and refined several sections of the text to improve clarity and precision. We hope that these revisions satisfactorily address the reviewers' suggestions and strengthen the manuscript's contribution to understanding how immune mechanisms shape HIV persistence.

Referee #1

In this study by Tan et al, the authors interrogated the impact of NK cells on HIV reservoir clearance, using the frequency of intact HIV provirus (versus defective HIV provirus) as a surrogate to measure the correlation between HLA-C2, HLA-E, and HLA-BBw4 recognition by NK inhibitory receptors KIR2DL, CD94/NKG2G, and KIR3DL1, respectively. The authors found that people having HLA-C2 and HLA-BBw4 have lower frequency of intact HIV proviruses, likely because of immune selection pressure, not initial infectivity (given that initial viral setpoint is not different). Using full-length HIV proviral sequencing and HLA typing, the authors found that HIV Vpu mutations correlate with lower frequency of intact HIV. The author continued and examined the E460A Gag variant (HLA-E presenting peptide) that correlates with the frequency of intact HIV. This is an innovative idea to explore how NK cells impose immune selection pressure on HIV, using HIV proviral sequence as a surrogate to identify footprints of NK selection pressure. The authors used a previously published method to examine NK cell proliferation by mitochondrial DNA sequence, which is innovative (although limited in applications, as only 2 participants went through this examination). However, the Vpu functional significance on HLA-C downregulation and NK-mediated killing should be validated in vitro beyond sequence correlations. Overall, this is an important study describing impact of NK cell on HIV reservoir. Yet, orthogonal functional validations beyond sequence correlations should be performed to justify the correlation-based nature of the observations.

Major comments

1. The correlative nature of this observation should be rigorously tested mechanistically. The impact of Vpu variants on HLA-C downregulation has been previously described by Mary Carrington group, which was cited by the authors. The authors should reconstruct Vpu variants, express in primary cells from people having HLA-C1 or HLA-C2, examine whether HLA-C is indeed downregulated (to what extent), infect these cells with HIV, and test whether HIV-infected cells in these Vpu variants induce same or different levels of NK cell killing. The authors should co-culture NK cells and HIV-infected autologous CD4 (with different reconstructed Vpu variants) and test NK cell activation, NK KIR2DL1 expression, KIR3DL1 expression, NKG2C expression, infected cell HLA-C expression level (by different Vpu variants, PMID: 27173934), and killing of HIV-infected cells (of different Vpu variants), NK cell proliferation (cell trace dilution) using flow cytometry. The authors should also test whether this killing is MHC I dependent by adding MHC blockers in these NK-infected cell co-culture killing assays.

We appreciate Reviewer 1's interest in the mechanistic link between vpu sequence variation and HIV-1 reservoir size. Our study shows that vpu variants shown to mediate strong HLA-C downregulation are associated with lower reservoir size specifically in carriers of HLA-C2. The identification of vpu variants with the capacity to downregulate HLA-C is grounded in extensive prior mechanistic work from our collaborators, Dr. Mary Carrington and Dr. Richard Apps. In that work (Bachtel et al., Plos Pathog 2018), they analyzed 128 vpu molecules isolated from ART-suppressed individuals and an additional 195 vpu sequences from treatment-naïve individuals, enabling a large-scale functional mapping of vpu sequence to HLA-C downregulation. This analysis identified six key amino acid residues that determine the ability of vpu to downregulate HLA-C. Since Apps and Carrington analyzed clade B viral variants, the overlap of residues of interest between their vpu sequences and the vpu sequences analyzed in our study (also in people living with clade B) is high (approximately 88%). The HLA-C downregulation capacity of these variants is therefore experimentally well documented.

To directly address the functional consequences of vpu-mediated HLA-C downregulation, we expanded the in vitro infection assays described in our original manuscript. We synthesized two viral constructs based on the NL4-3 backbone: (i) a virus containing the NL4-3 wild-type vpu sequence, which does not downregulate HLA-C, and (ii) a complementary virus in which NL4-3 vpu was replaced with the WITO-derived vpu sequence, which efficiently downregulates HLA-C, as shown in our work (Extended Data Fig. 9b,c) and in prior studies (ref 70 in the revised manuscript). In functional co-culture assays using autologous NK cells and CD4 T cells infected with these viruses, we demonstrate that KIR2DL1+ NK cells more efficiently recognize and respond to autologous CD4 T cells infected with the WITO-vpu–containing virus, consistent with enhanced susceptibility of these targets to KIR2DL1+ NK cell–mediated control due to "missing self" signal related to HLA-C downregulation.

In the revised manuscript, we have expanded these data as requested by the reviewer. Specifically, we now include (i) KIR3DL1, KIR2DL1 and NKG2C expression on NK cells in these co-cultures, and (ii) a broader assessment of NK cell function, including IFN-γ and TNF-α secretion as well as CD107 surface expression as a marker of cytotoxic degranulation (Extended Data Fig. 9d, e). We also include cumulative data on HLA-C expression on cells infected with the WITO-derived and the NL4-3-derived vpu sequence (Extended Data Fig. 9c). Together, these new experiments provide additional functional support for the proposed link between vpu sequence variation, modulation of HLA-C, and NK cell–mediated recognition of HIV-1–infected cells.

2. Similarly, the impact of Gag variants on HLA-E presentation to NK cells should be functionally tested as described above. The authors attempted with K562 cell transfection, which is a myeloid adherent cell line, not relevant to CD4 T cell-based reservoir profiling in this study. The authors should use primary CD4 T cell transfection (which may have more physiologically relevant levels of peptide presentation, given high levels of peptide presentation in myeloid cell lines versus modest level of peptide presentation in CD4 T cells.

While we recognize that HLA-E–transfected K562 cells are of myeloid origin, we consider this system the most appropriate reductionist model to interrogate peptide binding to HLA-E, because the lack of endogenous classical and non-classical HLA class I molecules minimizes confounding by alternative peptide–HLA interactions. By contrast, primary CD4 T cells express a broad repertoire of HLA class I and non-classical HLA molecules, which makes it technically challenging to specifically attribute observed effects to peptide binding and presentation by HLA-E alone.

In our revised manuscript, we have nonetheless expanded our dataset beyond the K562 system. First, we now include HLA-E stabilization experiments performed in HLA-E transfected RMA-S cells, a TAP-deficient mouse T cell line expressing HLA-E (and no other HLA alleles), which corroborate the differential stabilization capacity of the tested peptides. Second, we added an ELISA-based, cell-free peptide–HLA-E binding assay that independently confirms that variants of the RL9 epitope associated with higher reservoir size exhibit reduced HLA-E binding. Together, these complementary approaches strengthen our conclusion that specific sequence variants in HLA-E-restricted HIV-1 epitopes impair HLA-E presentation and thereby have the potential to modulate NK cell recognition.

3. The functional significance of these Vpu variants should be further examined regarding their significance, such as tetherin level [PMID: 23633949], SLC38A1 downregulation (PMID: 29324226), NF-κB inhibition, MHC downregulation [PMID: 30180214], and CD4 downregulation.

We thank the reviewer for highlighting the functional diversity of Vpu and suggesting further characterization of the identified variants across their known activities. While we agree that comprehensive testing of tetherin antagonism (PMID: 23633949), SLC38A1 downregulation (PMID: 29324226), NF-κB inhibition, MHC downregulation (PMID: 30180214), and CD4 downregulation would be informative, we respectfully submit that this extensive functional panel is beyond the scope of the current revision.

These additional Vpu activities—while critical for active viral spread—are mechanistically distinct from the HLA-C/NK cell recognition axis central to our study. Tetherin and SLC38A1 antagonism primarily enhance virion release and modulate T cell metabolism during acute replication such as in the case of untreated infection, respectively, rather than directly influencing NK-mediated immune pressure on reservoir cells under ART suppression. Our vpu variants were specifically selected based on their established effects on HLA-C downregulation, rigorously mapped by our collaborators (Drs. Carrington and Apps) through large-scale functional screening of participant-derived sequences. A broader Vpu functional atlas would be better suited to a dedicated study, to be conducted in future work.

4. Impact of NK cells on intact versus defective HIV-infected cells: In Figure 4, can the authors clarify if these Vpu sequences are solely from intact HIV? If not, do the authors mean cells harboring defective HIV can also be recognized by NK? Please separate Vpu

sequences identified from intact from defective HIV to dissect this question (whether the impact of NK on intact versus defective HIV is the same or different).

We thank the reviewer for this insightful question regarding the proviral origin of the Vpu sequences shown in Figure 4 (Fig. 5 in the revised manuscript) and the potential for differential NK cell recognition of intact versus defective HIV reservoirs. In response to this excellent suggestion, we performed paired analyses within the same 104 study participants, comparing key Vpu variants—specifically the six residues associated with HLA-C downregulation—between intact and defective proviral sequences. These new data show high concordance: in only one of the 104 participants did the analysis of intact proviruses yield a different "strong" or "weak" classification compared with the corresponding defective proviral sequences. We agree with the reviewer's perspective that defective proviruses may still be expressed and thereby influence HLA-C downregulation. Moreover, Vpu sequences from defective proviruses could reflect those present in intact proviruses that were not sampled, given that defective proviruses must have originated from once-intact ancestors during the evolutionary history of the proviral landscape. To incorporate the reviewer's suggestion, we have refined our analytical approach as follows: when three or more distinct intact proviruses were available for a given participant, Vpu sequences from these intact proviruses were used to determine the final "strong" or "weak" HLA-C downregulation classification, as we think they adequately represent the participant's proviral landscape. When fewer than three independent intact proviruses were available, the classification was based on Vpu sequences from both intact and defective proviruses, to better capture the overall Vpu sequence diversity in that participant. This adjustment results in only a minor change in the statistical analysis. We are grateful to the reviewer for highlighting this nuanced yet important point.

5. Impact of defective packaging signal on Vpu expression: recent studies of HIV low level viremia have identified the importance of packaging signals on the definition of intact HIV, which also affects Vpu RNA expression (Vpu expression requires splicing, which requires an intact HIV splice site within the packaging signal). The authors should align the packaging signal sequence of 366 intact HIV proviruses as a supplement figure and examine whether they are indeed intact, particularly the at the major splice donor site.

In response to the reviewer's point, we have analyzed our collection of 366 intact proviruses that were classified as intact. We observed that the major splice donor site was not present in 7 of these sequences, however, in these sequences, the upstream PCR primer site was also missing, indicating that these sequence defects likely reflect sequencing artifacts. This is a recognized phenomenon in studies related to near-full length proviral sequencing and has been observed in prior studies (e. g. Bruner, Siliciano et al, Nat Med 2016); the termed used for these proviruses in the literature is "inferred intact". Since this affects only a minor proportion of all proviruses (7/366), and these sequences were observed both in individuals with and without HLA-C2, these findings are unlikely to influence the final conclusions.

6. Line 171. The authors did a great job teasing out whether the decreased frequency of

intact HIV provirus is because of CD8+ T cell immune selection pressure, as the field currently sees. The authors examined and excluded that it's because of the typical alleles seen in elite controllers. However, the methods used to justify that these CD8+ T cell function across participants is not the cause of immune selection is week – the authors used TNF, IL-2, and IFNg staining, which is NOT what the field's consensus on CD8+ T cell function. The authors should measure CD8+ T cell proliferation (cell trace dilution), lytic granule expression (granzyme B, perforin), and killing assays.

We thank the reviewer for acknowledging our efforts to dissect CD8+ T cell versus NK cell contributions to frequencies of intact proviruses and for suggesting more comprehensive functional readouts. In response, we have expanded our assessment of HIV-specific CD8+ T cell functionality with a multi-parameter flow cytometry panel on PBMC from our study participants. These new data, now included in the revised manuscript (Extended Data Fig. 3a-d), quantify:

- ***Cytotoxic granule content**: Intracellular perforin and granzyme B staining in HIV-specific CD8+ T cells after stimulation with HIV gag, revealing no significant enrichment of lytic potential in participants with lower intact provirus frequency.*
- ***Degranulation capacity**: CD107a surface mobilization following peptide pool stimulation, as a direct functional readout of cytotoxic exocytosis.*

These assays confirm that participants with reduced intact provirus do not exhibit superior CD8+ T cell cytotoxic function by any measured parameter, robustly excluding CD8+ T cell pressure as the primary driver of our principal observation. Importantly, these readouts complement our original cytokine staining (IFN-γ, TNF, IL-2). Together, these enhancements strengthen our conclusion that NK cell-mediated selection provides the most parsimonious explanation for the observed provirus landscape.

Minor comments:
1. The authors should make a supplement table of the exact HLA haplotypes (A, B, C) of each study participant (including both alleles of each participant). Supplementary Table S4 is not sufficient for readers to recognized shared features versus differences across participants. In another column, list the lysine versus asparagine usage in the HLA-C allotypes. Do these allotypes follow typical textbook descriptions?
- KIR2DL1 (and KIR2DS1): Recognize HLA-C2 allotypes (e.g., HLA-Cw2, -Cw4, -Cw5, -Cw6, -Cw7, -Cw8, -Cw15, -Cw17, -Cw18), characterized by a Lysine (Lys80).
- KIR2DL2/KIR2DL3 (and KIR2DS2): Primarily recognize HLA-C1 allotypes (e.g., HLA-Cw1, -Cw3, -Cw7, -Cw8, -Cw12, -Cw14, -Cw16), characterized by an Asparagine (Asn80).

We have generated a new supplemental Table 3 summarizing the HLA class I alleles for all study participants, and the classification as C1 or C2 based on variations at position 80 is indicated.

2. uM should be corrected to μM.

3. Gag as a protein needs to have capitalized G; gag as a viral gene/RNA should be all lower case, italicized. Same for other viral nomenclature.

These minor comments were addressed as suggested by reviewer 1.

Referee #2

In this work the authors study NK cell biology in the context of reservoir decay under ART. Through an extensive series of experiments, the authors find that carriers of the HLA-C2 allele, along with expansion of HLA-E interacting NKG2C/KIR2DL1+ NK cells, are associated with reservoir decay of peripheral blood CD4+ T cells with intact HIV proviral DNA from donors on extended ART (cross-sectional cohort). HLA-E restricted peptides from HIV gag and Env are examined in detail for evidence of viral evolution or selection under ART. HIV vpu is shown to induce some degree of HLA-C downregulation, and vpu variants that differentially induce HLA-C downregulation are studied. The authors conclude that HIV reservoir decay occurring in HLA-C2+ donors on long term ART is associated with NK-cell mediated activity mediated through the KIR2DL1+NKG2C+ populations, wherein increased frequencies and activity of these cells is associated with decreased intact proviral DNA in the circulating CD4+ T cell reservoir.

General comments:

1. It is unclear how these findings provide actionable directions for the development of HIV cure strategies. While interesting from the pathogenesis and immunity standpoint, it is not immediately apparent that these studies will have a broad impact on the HIV cure field. This is alluded to in the discussion paragraph of the manuscript.

Actionable directions related to this work could involve pharmacological administration of NK cell immune checkpoint inhibitors (which may be associated with more limited toxicity compared to T cell checkpoint inhibitors), or CAR-NK cells. Nevertheless, we prefer to use cautious language in the discussion and emphasize the complexity and difficulty of targeting viral reservoir cells. We also submit that a combination of interventions may ultimately be necessary to effectively eliminate HIV-1 reservoir cells persisting during ART.

2. The data shown in the manuscript, while technically well performed and extensive, demonstrate associations, and not mechanisms, of potential control mediated by NK cells. All cohort based studies are cross-sectional in nature, yet interpret a longitudinal effect on the reservoir. No association is shown demonstrating time on ART with reservoir level, where one would expect a progressive decay with longer time on ART. Notably, it is expected that intact proviral DNA will decay during extended ART as has been shown by others. Is this accelerated in individuals with HLA-C2?

Longitudinal studies were indeed not part of our studies, all study participants in our work included people on long-term ART (median: 21 years). We have emphasized the need for longitudinal studies in the revised discussion of the manuscript. We would like to point out that surface phenotyping of HIV-infected cells (Fig. 5e-f) involved longitudinal studies.

3. It is not clear that the statistical differences observed in many of the analyses are biologically meaningful. The frequency of intact proviral DNA, for example, appears quite similar between the cohorts in many instances. Are these differences really meaningful? Would one expect viral rebound to be delayed in HLA-C2+ individuals due to the slightly lower reservoir? Lessons from nonhuman primates and other human studies suggest that circulating reservoir frequencies do not translate well to rebound time (unless the reservoir is extremely low). Likewise, it is unclear that the frequencies of the different NK cell functions between cohorts are biologically impactful. Is there evidence at a change in peripheral blood NK cell subset frequency of a few percent can have such a potential effect longitudinally on the HIV reservoir? Do these populations exhibit activity in tissues where the reservoir resides? The HLA-C 'downregulation' observed with vpu appears minimal (1-2 tenths of a log, statistical significance unknown). Is this sufficient to alter the recognition of HLA-C restricted epitopes by T cells or prevent NK cell recognition through NKG2C?

We now have stratified our study participants more comprehensively according to the host and viral genetic variations described in our manuscript. These results showed there was an approximately 7-fold difference in frequencies of intact proviruses between people with the most favorable and the least favorable host and viral genotypes. Such a difference is equivalent to the decay rate of intact proviruses during 10 years of ART, assuming a half-life of intact proviruses of 44 months (Fig. 5d). Tissue studies were not included in our work, and it is unfortunately impossible to collect lymphoid tissues from such a large cohort of ART-treated persons. The functional assays included in our manuscript suggest that HLA-C downregulation by Vpu is functionally relevant for NK cell recognition, as higher levels of NK cell immune recognition was noted when target cells were infected with HIV-1 containing vpu sequences able to downregulate HLA-C, relative to HIV-1 with vpu sequences that had no effect on HLA-C. This observation is further supported by lower reservoirs in persons with vpu variants most effective in downregulating HLA-C.

4. The cross-sectional design of the study reduces the strength of the results. The optimal study here would be to show preferential decay of intact proviral reservoirs over time within HLA-C2+ vs HLA-C2- donors on long term ART. Within these donors there would also need to be extensive analysis to rule out potential effects of other immune mechanisms, specifically T cells (see point 5).

We appreciate this comment and generally agree that longitudinal studies would be important for better understanding immune activities against the viral reservoir cell pool. Nevertheless, please note that we include strong population-level data showing that HLA-C2 had no protective effect during untreated HIV-1 infection, strongly supporting our hypothesis that the lower frequencies of intact proviruses in carriers of HLA-C2 reflect NK-cell mediated immune effects against HIV-1 reservoir cells. In addition, longitudinal studies are included for surface phenotyping of HIV-1 reservoir cells. We agree with the reviewer that longitudinal studies in the entire study cohort will be difficult to interpret, as both NK and T cells might act on reservoir cells, possibly at different time periods. We have amended our discussion to highlight the possibility that during early stages of ART,

viral reservoir cells may be predominantly under immune pressure from T cells, given that proviruses at this stage may be more transcriptionally active; infected cells surviving this stage may then be more adjusted to T cells (through e. g. HLA class I downmodulation), which would subsequently increase susceptibility to NK cells. We propose to conduct such longitudinal studies in future work.

5. Analysis of adaptive responses, especially T cells, is insufficient to rule out a potential effect of T cell mediated immunity on reservoir decay. Many of the HLAE associated peptides studied are also within known CD8+ T cell epitopes restricted by a broad array of HLA-A and B alleles. Notably, some of the same regions are associated with HLA-B57/58 epitopes. The T cell studies do not adequately rule out the possible role of T cell mediated pressure on the same regions.

As demonstrated in our manuscript, HLA-B27, -B57, and -B35Px—alleles associated with distinct clinical trajectories during untreated HIV infection—were not detectably associated with HIV-1 reservoir size, arguing against a major contribution of T cell–mediated immunity to reservoir control. However, we acknowledge the possibility that CTL epitopes restricted by classical HLA class I alleles and overlapping with HLA-E–restricted epitopes could confound our interpretation. To address this, we performed a detailed analysis of such overlapping epitopes. Notably, sequence variants within these regions were not associated with viral reservoir size when individuals carrying the relevant classical HLA alleles (that restrict CTL epitopes overlapping with HLA-E-restricted epitopes) were compared to non-carriers (Extended Data Fig. 7a–c).
Moreover, differences in reservoir size between individuals harboring variant versus wild-type HLA-E epitope sequences were not restricted to carriers of the corresponding classical HLA alleles. This pattern would be expected if the observed effects were driven by CTL responses. More broadly, it appears unlikely that CTL-mediated immunity accounts for our findings, given the substantial heterogeneity of classical HLA alleles across our cohort of 104 individuals, in contrast to the uniform presence of HLA-E as a shared immunological feature. Effects linked to HLA-E are therefore more consistent with cohort-wide mechanisms.
Finally, the impact of sequence variation within HLA-E–restricted epitopes on reservoir size was observed exclusively among HLA-C2 carriers, but not in non-carriers. This observation further supports the NK cell education model proposed in our study.

6. The expansion of NKG2C+ NK cells, as stated by the authors, is known to be associated with CMV. CMV is also associated with increased KIR2DL1+ NK cells. While the authors state that “CMV infection did not seem to represent a sufficient factor”, I feel that CMV is a major driver of the effect given the welldocumented effect on these subsets of NK cells after CMV acquisition.

We agree that CMV plays an important role for driving the expansion of NKG2C+ NK cells, as almost our entire study cohort (>98%) consisted of people with HIV/CMV co-infection. However, the proportion of NKG2C+ NK cells varied profoundly between CMV-positive carriers, ranging from 1.37% to 79.5% in total NK cells. This, in our opinion, indicates that factors other than CMV are involved in co-determining expansion and clonal

proliferation of NKG2C+ NK cells. We agree with the reviewer that CMV infection is clearly one aspect that drives expansion of NKG2C+ NK cells, and future studies will be helpful to better understand how CMV co-infection influences immune activity against HIV reservoir cells.

7. Do the authors feel that there is evidence of vpu sequence evolution taking place, or vpu sequence selection? Presuming under long term fully suppressive ART, depending on who we believe in the literature, there should not be ongoing rounds of viral replication. As such, how would vpu be evolving under innate immune pressure? Or is this selection occurring within the reservoir? Here, it would be important to understand the variation of this region of vpu prior to ART initiation when the reservoir presumably forms. I understand the complexity of this question, but this issue is central to interpretation of the entire manuscript,
and needs to be carefully considered.

We agree that these are very important questions, and that longitudinal studies would be required to address them. Our lab is now involved in conducting such longitudinal work in great detail, however, such additional work is beyond what can be included in the current manuscript.

Technical comments.
1. The mitoseq and phepseq studies, while interesting and technically demanding, provide insufficient support for their respective conclusions due to the low n (2 per cohort) studied. The value of these analyses to the overall conclusion is thus limited.

We emphasize that these studies are sophisticated and difficult, as well as expensive, to conduct. In our opinion, these results strongly support the proposed hypotheses.

2. It is not possible to determine the validity of the flow-based assays due to the absence of flow gating examples to ensure that the large number of NK cell markers can be individually resolved. Use of the SPICE program is archaic; there are more robust ways to represent T cell function data.

We have added additional flow diagrams to demonstrate our flow cytometric gating strategy (Extended Data Fig. 10). We have added additional data in the flow cytometric analysis of HIV-specific CD8 T cell responses; please see also comments to reviewer 1 (Extended Data Fig. 3).

3. The sequence analysis of vpu in Figure 4A appears to show an insert at position 7 relative to the consensus clade B vpu sequence available on the LANL database. If the sequences are correct as shown, and an insertion is truly present, there is concern whether vpu function in these donors is altered given that this area has multiple transmembrane regions.

Figure 4A reflects a summary of vpu sequence diversity in our large study cohort of ART-treated persons. Comparing these participant-derived sequences to the clade B

consensus sequence from LANL, we do not see evidence of an insertion at position 7 in our data. It is known that laboratory strains such as the NL4-3 sequence (which cannot downregulate HLA-C) is missing a residue at position 5, relative to the clade B consensus sequence.

Referee #3

In their study, Seng Tan and colleagues have explored the impact of immune responses on the decay of the HIV-1 reservoir during ART. In particular, they found that the presence of HLA B and C alleles with Bw4 and C2 motifs, known to influence NK cell education, was associated with reductions in the levels of total and/or intact proviruses. They further observed that the frequency of NK cells expressing KIR2DL1, which recognizes HLA-C2, and in particular those co-expressing NKG2C, was negatively correlated with the level of intact proviruses in people with HIV-1 on long-term treatment. To support the role of these cells in actively reducing the viral reservoir, the authors showed that the presence of HIV-1 vpu variants that downregulate HLA-C were associated with smaller reservoirs in individuals with HLA-C2 alleles, whereas escaping variants in epitopes restricted by HLA-E, the ligand for NKG2C, were associated with higher frequencies of intact proviruses. These results support an important role for NK cells in the dynamics of the viral reservoir during ART. This is a strong study in which the authors have made a substantial effort to consolidate their observations; however, several points would benefit from further clarification.

One limitation of the study is the absence of data from the period before ART initiation in the study cohort. In this context, it is not possible to exclude that NK cells may have influenced the seeding of the viral reservoir prior to ART initiation. The authors have addressed this limitation by showing that no differences were observed in viremia levels among seroconverters carrying or not carrying the motifs of interest. However, there is not an absolute equivalence between viremia and the level of proviruses. How reliable is the correspondence between viremia and viral DNA during seroconversion? Moreover, it is not clear whether the individuals primarily analyzed here were treated during seroconversion or at later time points, which could have allowed prolonged NK cell activity against cells with active viral replication. Therefore, the sentence in the abstract "we found that carriers of HLA-C2 allotypes, which promote NK cell education via interactions with KIR2DL1, exhibited lower frequencies of intact proviruses, whereas no such associations were observed during untreated infection" is not entirely accurate considering the data presented in the manuscript.

We thank the reviewer for this thoughtful and important comment regarding the absence of pre-ART data and the potential influence of NK cells on reservoir establishment prior to treatment initiation. We fully agree that without longitudinal samples spanning the untreated phase, it is not possible to definitively exclude an early role of NK cells in shaping the reservoir. Our intention was not to imply the contrary but rather to highlight that, based on available data, we did not detect evidence supporting such an effect.

Specifically, we compared plasma viremia levels among untreated individuals carrying versus not carrying the HLA-C2 motif and observed no significant differences. While we acknowledge that plasma viremia does not perfectly mirror the frequency or composition of infected cells, we argue that during untreated infection, viral load represents the best marker reflecting antiviral immune activity. Nevertheless, we recognize that the correspondence between viral load and intact proviruses has not formally been demonstrated (to our knowledge), and residual differences at the cellular level cannot be excluded.

Regarding treatment timing, none of the study participants from the untreated group had received ART at the time of sample collection. We have revised the Abstract and Results sections to clarify this point and to temper our interpretation. The updated sentence now reads: "By analyzing more than 6,000 viral DNA amplicons from 104 individuals on long-term ART, we found that carriers of HLA-C2 allotypes, which promote NK cell education via interactions with KIR2DL1, exhibited lower frequencies of intact proviruses, whereas no protective effects of HLA-C2 alleles were observed during untreated infection, suggesting a selective vulnerability of reservoir cells to NK cell-mediated immune activity under ART. "

While the analyses in figure S2C exclude individuals with HLA-B*27, B*57 and B*35PX alleles, the analyses in figure 1E do not consider the latter. were there not any individuals carrying these alleles in that cohort?

*We considered this analysis for Figure 1e; however, it is difficult to meaningfully conduct such an analysis because HLA-B*35Px allele frequencies are strongly confounded by race in the untreated cohort. As an alternative, we performed logistic regression adjusting for HLA-B*27, B*57, and B*35Px alleles alongside race, with results presented in Table 2. This analysis revealed no significant association between viral load control and the presence of C2 or BBw4 alleles.*

The authors found lower reservoir levels in individuals carrying HLA-B alleles with Bw4 motifs; however, a few HLA-A alleles also contain the Bw4 epitope. Was this taken into account in the analyses, either when evaluating the effect of Bw4 or C2 alleles?

We have analyzed possible viral reservoirs in individuals carrying HLA-A Bw4 alleles; however, there was no statistically significant effect (Extended Data Fig. 2e), most likely because HLA-ABw4 alleles have a lesser effect on NK cell education. We have edited the manuscript to address this.

There appears to be a synergistic effect of Bw4 and C2 on the size of the viral reservoir; however, the contribution of Bw4 is not thoroughly explored in the manuscript. Although the specific role of Bw4 might warrant a dedicated study, its potential relevance is surprisingly underexplored in the discussion of the results. Moreover, a recent report from the VISCONTI study identified an immunogenetic signature combining the presence of Bw4 and C2 alleles, which were found to be more frequent among post-treatment

controllers (Essat et al., Med, 2025). Could this association be related to the findings presented here by the authors?

We have cited the work by Essat et al. in our paper and argue that both papers suggest a role for KIR-educated NK cells in enhancing antiviral immune activity. Given that we did not find a correlation between KIR3DL1-expressing NK cells and HIV reservoir size, we argue in our manuscript that NK cell education by HLA-BBw4-KIR3DL1 interactions is less relevant for influencing HIV-1 reservoir cells, however, we have edited the manuscript to indicate that additional studies will be necessary to better explore the effect of HLA-BBw4 on HIV persistence.

The authors analyzed the effect of HIV-1 peptides that have been reported to influence the stability of HLA-E molecules; however, they did not consider previous work (including by some of the same authors) on the potential impact of HLA class I signal peptides on HLA-E expression (Horowitz 2016; Ramsuran 2018; Lin 2023).

Many thanks for this comment. We have now included additional data to show that the HLA-B21 M/T signal peptide polymorphism has no influence on viral reservoir cell frequencies (Extended Data Fig. 2f,g).

The authors performed an elegant analysis to explore the dynamics of NK cells in individuals on ART, and their results "imply that expansion of KIR2DL1$^+$ KIR3DL1$^-$ NKG2C$^+$ NK cells in C2-expressing individuals on ART is at least in part driven by clonal proliferation." However, the authors had mentioned that these cells displayed features of adaptive NK cells, which generally exhibit limited or absent proliferative potential. Could the authors assess the expression of CD57 in this experiment? It would also be interesting to evaluate the fate of KIR2DL1$^+$ KIR3DL1$^-$ NKG2A$^+$ NK cells, as these cells share the same ligands but differ in their activating versus inhibitory signaling.

The KIR2DL1+ NKG2C+ KIR3DL1- NK cells also frequently co-expressed CD57, consistent with being highly educated NK cells; we have now expanded our data to highlight CD57 expression on NK cells (supplemental Figure 4I). We have investigated evidence for clonal expansion within the NKG2A+ NK cell pool, both within KIR2DL1+ and KIR3DL1+ cells, but we have not found evidence for clonal proliferation in this cell population, as evidenced by mitochondrial DNA sequence variations, in our study.

Along these lines, to further strengthen the proposed role of NKG2C$^+$ cells, it would be valuable to evaluate the impact of vpu variants and HLA-E epitopes on the recognition of infected cells by KIR2DL1$^+$ NKG2A$^+$ NK cells.

In our in vitro infection assays, we noticed that the population of NKG2A+ KIR2DL1+ NK cells is very small (around 2% of all NK cells), which makes it difficult to analyze the specific responsiveness of this cell population. With these limitations, we observed that NKG2A+ KIR2DL1+ did not differentially respond to autologous CD4 T cells infected with vpu sequences that were or were not able to downregulate HLA-C. Moreover, there was no evidence that NKG2A+ KIR2DL1+ NK cells responded differentially to cells infected

with viruses that encoded variants in HLA-E-restricted epitopes. This is, in our opinion, not unexpected, as NKG2A seems to have a weaker ability to recognize HLA-E epitopes in a peptide variant-specific fashion.

The presence of HLA-C2 was associated with a decrease in all reservoir cells (containing either defective or intact proviruses). I wonder whether, among the defective proviruses, a distinction could be made between those carrying defects affecting vpu and those without.

We regret that we are unable to address this since many of defective proviruses were not sequenced. Per our standard protocol, we only sequenced proviruses that are near full-length (>8 kb) in a gel electrophoresis assay. Our study involved more than 6000 proviruses, and sequencing all of these amplification products (many of them may have vpu deletions) individually is unfortunately not possible with the sequencing resources available to our lab.

Lines 203-205:"Interestingly, KIR2DL1+ KIR3DL1- NK cells also frequently expressed DNAM-1, CD57, KLRG1, while having limited surface expression of the inhibitory HLA-E receptor NKG2A". The NKG2C/NKG2A ratio appeared to be strongly influenced by CMV infection, as previously reported.

In response to this comment, we have analyzed the ratio of NKG2C/NKG2A NK cells in our datasets; however, we failed to detect an association between this ratio and viral reservoir cells. This was true for both the KIR2DL1+ and the KIR2DL1- NK cell subset.

Minor comments

Panels in figure 4E are not easy to evaluate, it appears to exist a trend towards reduced HLA-E expression in individuals with HLA-C2? (in particular in "late" samples). Are there changes in relative HLA-C expression, even among non infected cells, early vs late in C2+ and in C2- individuals?

In this complex analysis, we did not observe differences in HLA-E expression between individuals with or without HLA-C2. Comparisons across timepoints in this experimental system are challenging, as samples are processed on separate microfluidic cartridges. Within this technical limitation, we found no statistically significant differences in HLA-C expression between early and late timepoints.

the medians are difficult to appreciate in many of the scattered plot panels.

We have revised the figures to improve the graphical presentation of the medians.

Supplemental table 1: CMV seropositive status should read 13/0 & 18/0 in the Bw4-C2+ & Bw4+C2- columns

Many thanks for picking this up - we have corrected this as recommended.

The authors should provide basic information concerning HIV-1 infection (at least duration on ART) for individuals described in Supplementary table 4.

We have edited supplementary Table 4 (now Supplemental Table 2) as suggested.

Reviewer #1 (Remarks to the Author):

In this study by Tan et al, the authors interrogated the impact of NK cells on HIV reservoir clearance, using the frequency of intact HIV provirus (versus defective HIV provirus) as a surrogate to measure the correlation between HLA-C2, HLA-E, and HLA-BBw4 recognition by NK inhibitory receptors KIR2DL, CD94/NKG2G, and KIR3DL1, respectively. The authors found that people having HLA-C2 and HLA-BBw4 have lower frequency of intact HIV proviruses, likely because of immune selection pressure, not initial infectivity (given that initial viral setpoint is not different). Using full-length HIV proviral sequencing and HLA typing, the authors found that HIV Vpu mutations correlate with lower frequency of intact HIV. The author continued and examined the E460A Gag variant (HLA-E presenting peptide) that correlates with the frequency of intact HIV. This is an innovative idea to explore how NK cells impose immune selection pressure on HIV, using HIV proviral sequence as a surrogate to identify footprints of NK selection pressure. The authors used a previously published method to examine NK cell proliferation by mitochondrial DNA sequence, which is innovative. Overall, this is an important and innovative study describing impact of NK cell on HIV reservoir. This study is already highly polished with no weaknesses found. I do not have suggestions for additional edits.

We thank Reviewer#1 for the positive and encouraging comments on our work and for recognizing the novelty and potential significance of our findings. We greatly appreciate the reviewer's thoughtful and insightful assessment of the study.

Reviewer #4 (Remarks to the Author):

Tan et al. Innate innate immune imprints shape HIV-1 reservoir cell persistence during long-term antiretroviral therapy.

In this work the authors study NK cell biology in the context of reservoir decay under ART. Through an extensive series of experiments, the authors find that carriers of the HLA-C2 allele, along with expansion of HLA-E interacting NKG2C/KIR2DL1+ NK cells, are associated with reservoir decay of peripheral blood CD4+ T cells with intact HIV proviral DNA from donors on extended ART (cross-sectional cohort). HLA-E restricted peptides from HIV gag and Env are examined in detail for evidence of viral evolution or selection under ART. HIV vpu is shown to induce some degree of HLA-C downregulation, and vpu variants that differentially induce HLA-C downregulation are studied. The authors conclude that HIV reservoir decay occurring in HLA-C2+ donors on long term ART is associated with NK-cell mediated activity mediated through the KIR2DL1+NKG2C+ populations, wherein increased frequencies and activity of these cells is associated with decreased intact proviral DNA in the circulating CD4+ T cell reservoir.

I previously reviewed this manuscript for Nature, and note that this version has addressed a few of my concerns; however, the manuscript is largely unchanged in content despite some reorganization to match journal style. It appears that some data previously included as supplemental figures are now found in the main manuscript, and some figures have been reorganized. Some additional text and information have been added as well.

However, most of my comments and concerns from the previous round were not addressed and therefore still stand.

The expanded discussion raises new points that are important advancements in the field. While I maintain my statement below regarding actionable directions, it is nevertheless important to consider that NK cells might have some influence on the reservoir during ART. The authors bring up that these data may revise our thoughts on the need for 'shock and kill' strategies, but I am not so convinced of this. To some degree, it depends on what one considers the 'latent' reservoir, a definition that varies depending on whom you ask. If one considers a full-length provirus integrated into heterochromatic DNA, with itself packaged into heterochromatin matching the background region such that no viral transcripts of any sort are produced, one may need a shock to make this virus visible to the immune system.

The new paragraph (lines 427-453) is a very nice encapsulation of how T cells and NK cells could work together at different phases after ART initiation to impose differential pressure on the evolving state of the HIV reservoir. The points raised reinforce my previous comment that this study needs longitudinal, rather than cross-sectional, assessments. I appreciate that this is extremely difficult given the need for long term follow up and availability of samples. This may not be achievable—but at the very least it should be addressed.

General comments:

1. It is unclear how these findings provide actionable directions for the development of HIV cure strategies. While interesting from the pathogenesis and immunity standpoint, it is not immediately apparent that these studies will have a broad impact on the HIV cure field. What are the authors proposing to make these findings clinically valuable?
2. The data shown in the manuscript, while technically well performed and extensive, demonstrate associations, and not mechanisms, of potential control mediated by NK cells. All cohort based studies are cross-sectional in nature, yet interpret a longitudinal effect on the reservoir. No association is shown demonstrating time on ART with reservoir level, where one would expect a progressive decay with longer time on ART. Notably, it is expected that intact proviral DNA will decay during extended ART as has been shown by others. Is this accelerated in individuals with HLA-C2?
3. It is not clear that the statistical differences observed in many of the analyses are biologically meaningful. The frequency of intact proviral DNA, for example, appears quite similar between the cohorts in many instances. Are these differences meaningful? Would one expect viral rebound to be delayed in HLA-C2+ individuals due to the slightly lower reservoir? Lessons from non-human primates and other human studies suggest that circulating reservoir frequencies do not translate well to rebound time (unless the reservoir is extremely low). Likewise, it is unclear that the frequencies of the different NK cell functions between cohorts are biologically impactful. Is there evidence at a change in peripheral blood NK cell subset frequency of a few percent can have such a potential effect longitudinally on the HIV reservoir? Do these populations exhibit activity in tissues where the reservoir resides? The HLA-C 'down-regulation' observed with vpu appears minimal (1-2 tenths of a log, statistical significance unknown). Is this sufficient to alter the

recognition of HLA-C restricted epitopes by T cells or prevent NK cell recognition through NKG2C?
4. The cross-sectional design of the study reduces the strength of the results. The optimal study here would be to show preferential decay of intact proviral reservoirs over time within HLA-C2+ vs HLA-C2- donors on long term ART. Within these donors there would also need to be extensive analysis to rule out potential effects of other immune mechanisms, specifically T cells (see point 5).
5. Analysis of adaptive responses, especially T cells, is insufficient to rule out a potential effect of T cell mediated immunity on reservoir decay, as only responses to Gag were measured.
6. The expansion of NKG2C+ NK cells, as stated by the authors, is known to be associated with CMV. CMV is also associated with increased KIR2DL1+ NK cells. While the authors state that "CMV infection did not seem to represent a sufficient factor", I feel that CMV is a major driver of the effect given the well-documented effect on these subsets of NK cells after CMV acquisition.
7. Do the authors feel that there is evidence of vpu sequence evolution taking place, or vpu sequence selection? Presuming under long term fully suppressive ART, depending on who we believe in the literature, there should not be ongoing rounds of viral replication. As such, how would vpu be evolving under innate immune pressure? Or is this selection occurring within the reservoir? Here, it would be important to understand the variation of this region of vpu prior to ART initiation when the reservoir presumably forms. I understand the complexity of this question, but this issue is central to interpretation of the entire manuscript, and needs to be carefully considered.

Technical comments.
1. The mitoseq and phepseq studies, while interesting and technically demanding, provide insufficient support for their respective conclusions due to the low n (2 per cohort) studied. The value of these analyses to the overall conclusion is thus limited.
2. The sequence analysis of vpu in Figure 5A appears to show an insert at position 7 relative to the consensus clade B vpu sequence available on the LANL database. If the sequences are correct as shown, and an insertion is truly present, there is concern whether vpu function in these donors is altered given that this area has multiple transmembrane regions.

We thank Reviewer 4 for their comments. We note that these remarks repeat the comments raised in the initial review. As such, we have already addressed them in our earlier responses and do not have additional points to add at this time. We appreciate the reviewer's continued interest in our work.

Reviewer #6 (Remarks to the Author):

A. Summary of the key results
Tan, Sun, Gao et al. present a tour-de-force analysis of proviral reservoir sequences, host immunogenetic, and antiviral immune responses in the setting of HIV infection that challenges the existing paradigm that the HIV reservoir is largely invisible to the immune system. In this fascinating manuscript, they instead suggest that NK cells, not T cells, play

a role in recognizing reservoir cells, and that this recognition is through a combination of missing-self responses through KIR2DL1 and is aided by NKG2C-dependent recognition of HLA-E-restricted HIV-1 epitopes. In addition to challenging the paradigm of reservoir 'silence' this paper highlights a fundamental and previously unappreciated aspect of HIV biology—the fact that the immune responses important in untreated infection, where T cell responses are the most important, are quite distinct from those during treated infection when NK cells dominate. This paper therefore has important ramifications for our fundamental understanding of HIV biology during treatment and for our approach to cure strategies. This paper highlights the complex interplay between viral (particularly vpu) and host genetics and indeed opens the door for more personalized treatment strategies. Overall, I feel this paper is of great interest to the field. There are a few places where the conclusions could be strengthened as outlined below.

We thank Reviewer #6 for the thoughtful, enthusiastic, and insightful assessment of our work. We are also encouraged by the reviewer's recognition of the integrated nature of our study, combining proviral sequencing, host immunogenetics, and functional immunologic analyses to investigate the interplay between viral and host factors. We greatly appreciated the reviewer's constructive suggestions below, which have helped us further strengthen the manuscript.

B. Originality and significance: if not novel, please include reference

This is a highly original and significant paper.

C. Data & methodology: validity of approach, quality of data, quality of presentation

Overall, the data and methodology are very clear with excellent presentation. However, the following major and minor comments would significantly improve the presentation and support the claims better.

Major comments

1. Linking claims to displayed data (Fig. 3b; Fig. 5f)

In Fig. 3b, the text links stronger clonal proliferation in P1 vs P2 to P1 having a lower reservoir, but reservoir measurements are not shown in that panel. Please add a cross-reference to where these reservoir metrics are displayed or temper the wording.

We thank Reviewer #6 for this helpful suggestion. To address this concern, we have revised the manuscript to include the reservoir measurements and the proportion of KIR2DL1+KIR3DL1-NKG2C+ NK cells for P1 and P2, providing additional context for the observed differences in clonal expansion between these individuals.

In Fig. 5f, since the main text emphasizes the C2-carrier interpretation, please briefly describe the pattern in C2 non-carriers and discuss why intact-provirus cells can show

lower (or not higher) HLA-C than the broader HIV-DNA+ pool in some strata (biological or technical explanations).

*We thank Reviewer #6 for this helpful comment. It is indeed interesting that in the two C1 carriers in **Figure 5f,** HIV-1-infected cells analyzed 1 year after ART initiation demonstrate reduced HLA-A, -B, -C expression compared to uninfected cells. We propose that this may represent a selection effect likely driven by HIV-specific T cells; infected cells with lower levels of HLA-A, -B, -C likely have a survival advantage when T cells represent the primary immunological threat. However, during long-term ART in HLA-C2 carriers, an opposite finding is made; here, infected cells harboring intact proviruses have higher HLA-C levels, prompting us to hypothesize that NK cells represent the primary driver of immune selection at this time. We have suggested such a sequential model of immune selection, with T cells acting as the dominant selection force during untreated or early-stage treated infection and NK cells acting as a driver of immune selection during long-term ART, in our discussion. We have also adjusted the language in the results section of the manuscript to address this.*

2. C2-specificity as effect modification (Fig. 4a; optionally Fig. 5f)

Because a central claim is that variant–reservoir associations are preferentially observed in HLA-C2 carriers, it would strengthen the presentation to report a formal interaction/effect-modification test (variant × HLA-C2) or otherwise provide stratum-specific effect sizes (with CIs) to complement p-values.

*We thank the reviewer for this excellent suggestion, which has significantly strengthened the statistical rigor of our manuscript. As requested, we have performed a formal test for effect modification by constructing multivariable linear regression models that incorporate explicit multiplicative interaction terms between HLA-C2 status and the individual viral variants in HLA-E-restricted epitopes. Consistent with our prior observations, these models revealed statistically significant interactions for the E460A variant, the R747P variant and the Y79F variant with HLA-C2. These significant interaction terms formally confirm a synergistic effect, demonstrating that these variants within HLA-E-restricted epitopes modify (and effectively abrogate) the protective association of the HLA-C2 allele. To complement these findings and provide clear, stratum-specific context as requested, we have reported these formal interaction test results alongside the corresponding stratum-specific effect sizes and confidence intervals in **Extended Data Figure 6f**. The main text and Methods section have been updated to reflect these additions.*

3. Mechanistic inference around NKG2C/HLA-E and missing-self (Fig. 4d–e)

Fig. 4d/ED8 support generally weak HLA-E stabilization by HIV peptides and modest WT–variant differences for selected epitopes. Please consider reporting quantitative curve summaries (EC50/max/AUC) and use “surface stabilization” language consistently, reserving “affinity” for cases with direct binding evidence.

*We thank Reviewer #6 for this helpful suggestion. We have now included a new supplemental Table 6 summarizing the AUC for all peptides assayed in **Figure 4d** and **Extended Figure 8c** in this revision. In the original text, we intended to relate the reduced HLA-E surface stability to diminished binding affinity. For a more cautious tone, we have tempered the language to "potentially suggesting diminished HLA-E binding affinity". While several HLA-E–presented peptides exhibit limited capacity to stabilize HLA-E, these lower-stability complexes may still be able to mediate biologically relevant immune effects. Indeed, such conformers are likely preferentially recognized by NKG2C over NKG2A, consistent with the greater tolerance of NKG2C for low-stability HLA-E–peptide complexes. Accordingly, these peptides could be well positioned to promote NKG2C-mediated activation without eliciting NKG2A-dependent inhibition.*

Fig. 4e/ED9 is a strong test of the missing-self + HLA-E/NKG2C model (with HLA-C downregulation and infection gating controls). To tighten causality, consider tempering language to "consistent with NKG2C involvement" unless receptor/ligand perturbation (e.g., NKG2C or HLA-E blockade) is available. Also, please cite ED9E (TNF-α, CD107a) in the main-text interpretation.

*We thank Reviewer #6 for this helpful suggestion. We have now cited **Extended Data Fig. 9e** in the text and tempered the language to "NKG2C involvement" instead of "mediated by NKG2C" in this revision.*

Minor comments

Extended Data Fig. 4i is descriptive within the KIR2DL1+KIR3DL1− gate; if possible, add an enrichment vs a reference NK subset (within-donor) or slightly temper the "adaptive NK" phrasing.

We have adjusted the phrasing for this figure to indicate that surface marker expression was selectively analyzed on KIR2DL1+KIR3DL1- NK cells, and adjusted the language for "adaptive NK cells".

A. Extended comments (figure-linked, detailed)

Extended Figure 4i:

Extended Data Fig. 4i provides useful descriptive information on marker expression within the KIR2DL1+KIR3DL1− subset across strata. To better support the statement that these cells show "adaptive-like" features, it would be helpful to explicitly indicate whether these markers are enriched relative to an internal reference NK population (e.g., total CD56dim or another KIR-defined subset), ideally using a within-donor comparison or an enrichment metric. If additional canonical "adaptive" markers (e.g., CD62L and/or NKG2A) are available in the dataset, briefly referencing them alongside the current markers would strengthen the interpretation; otherwise, the text could be slightly tempered to indicate that the phenotype is 'consistent with' rather than definitive for adaptive NK cells.

We thank reviewer #6 for the helpful advice, we altered the text to indicate that the described phenotype is consistent with adaptive NK cells. We also indicated that the KIR2DL1+ NKG2C+ NK cells expressed additional markers previously described on adaptive NK cells, such as CD57, DNAM-1 and KLRG1.

Figure 3b:

The P1 vs P2 example is a clear illustration of mtDNA-defined NK clonality. However, the Results text links the stronger clonal proliferation in P1 to P1 having a lower reservoir (citing Fig. 3b), while Fig. 3b itself does not display reservoir measurements. Please either add a cross-reference to where the reservoir metric for these two participants is shown (main or supplement) or adjust the wording to keep Fig. 3b focused on clonality/phenotype without implying a reservoir difference that is not displayed in that figure.

We thank Reviewer #6 for this helpful suggestion. To address this concern, we have revised the manuscript to include the reservoir measurements and the proportion of KIR2DL1+KIR3DL1-NKG2C+ NK cells for P1 and P2, providing additional context for the observed differences in clonal expansion between these individuals.

Figure 4a:

Fig. 4a clearly presents variant–reservoir associations stratified by HLA-C2 status and supports the stated “C2-specific” pattern for several variants. To strengthen this claim statistically (and to help readers interpret whether apparent differences between strata reflect true effect modification), it would be helpful to report a formal interaction/effect-modification test (variant × HLA-C2) using an appropriate model for the reservoir outcome. If such modelling is not feasible, please state this explicitly and consider reporting effect sizes (with confidence intervals) within each stratum to complement the p-values.

As reported above, we have calculated a linear regression model incorporating multiplicative interactions terms, which support a synergistic effect and demonstrate that specific variants in HLA-E-restricted epitopes modify the protective association of the HLA-C2 allele.

Figure 4d:

Fig. 4d, together with Extended Data Fig. 8, supports the authors’ conclusion that HIV-derived HLA-E epitopes generally form relatively weak/low-stability HLA-E complexes compared with the VL9 control, and that WT–variant differences are modest and largely evident at higher peptide concentrations for RLVDGFLAL and KALGPAATL. To improve interpretability and cross-epitope comparison, please consider reporting quantitative curve summaries (e.g., EC50 and maximal stabilization or AUC) for WT vs variant peptides. Additionally, given that stabilization is an indirect readout influenced by peptide loading/exchange kinetics, it may be preferable to consistently describe the findings in

terms of “HLA-E surface stabilization/steady-state expression,” while reserving “binding affinity” language for cases directly supported by the ELISA-based binding data (as shown for RLVDGFLAL). For epitopes with minimal stabilization differences, a short note clarifying whether this reflects assay sensitivity/kinetic limitations versus truly negligible effects would help align these in vitro results with the downstream functional assay in Fig. 4e.

We thank Reviewer #6 for this helpful suggestion. We have now included a new ***Supplemental Table 6*** *listing the AUC for all peptides assayed in* ***Figure 4d*** *and* ***Extended Figure 8c*** *in this revision. In the original text, we intended to relate the reduced HLA-E surface stability to diminished binding affinity. For a more cautious tone, we have tempered the language to “potentially suggesting diminished HLA-E binding affinity”. It is interesting that amino acid variations in HLA-E-restricted peptides with limited or almost undetectable HLA-E stabilization are still associated with altered viral reservoir size, suggesting an immune effect that is not completely captured by HLA-E stabilization assays but becomes functionally relevant in NK cell recognition assays (****Figure 4e****). We argue that these peptides with low abilities to stabilize HLA-E may still induce biologically relevant effects, due to the ability of NKG2C to recognize and tolerate low-affinity HLA-E conformers. We propose that the immunological significance of these non-canonical, pathogen-derived HLA-E-binding peptides deserve further investigation in future studies.*

Figure 4e:

Fig. 4e, together with Extended Data Fig. 9, provides a coherent experimental test of the “missing-self + HLA-E/NKG2C” model. The inclusion of Vpu constructs that do versus do not downregulate HLA-C, as well as the accompanying HLA-C downregulation measurements and comparable infection gating (p24+), are strengths and address key confounders.

1. To further tighten the causal interpretation that the peptide-variant effect is mediated specifically through NKG2C–HLA-E, it would be helpful to either (i) modestly temper the wording from “mediated by NKG2C” to “consistent with NKG2C involvement,” or (ii) include (if available) a receptor/ligand perturbation such as NKG2C or HLA-E blockade. While not strictly required given the subset-level specificity, such perturbation would strengthen the mechanistic claim.

We have now cited ***Extended Data Fig. 9e*** *and tempered the language to “NKG2C involvement” instead of “mediated by NKG2C”in this revision. We agree with the reviewer that the selective observation of the described functional effects in the KIR2DL1+ NKG2C+NKG2A- NK cell subsets supports the proposed conclusion that missing-self recognition via KIR2DL1 and NKG2C-dependent recognition of HLA-E epitopes jointly induce the observed functional NK cell responses.*

2. Extended Data Fig. 9E includes additional functional readouts (TNF-α and CD107a), which is helpful. Please consider explicitly citing these data in the main-text interpretation of Fig. 4e so that the conclusion is not based solely on IFN-γ.

We thank Reviewer #6 for this helpful suggestion. We have now cited ***Extended Data Fig. 9e****.*

3. Because the functional assay uses “triple” Gag constructs, the experiment convincingly addresses the combined effect of multiple epitope variants but does not resolve whether one epitope dominates or whether effects are additive versus epistatic. Please briefly acknowledge this as an interpretive limitation when connecting the mechanistic assay to the individual-variant reservoir associations shown in Fig. 4a/b

We thank Reviewer #6 for this helpful advice. We have now acknowledged this limitation in the results section when describing the functional NK cell assays in the context of how sequence variations in HIV-derived HLA-E epitopes associate with altered viral reservoir size.

Figure 5f:

Fig. 5f is helpful in stratifying HLA-C expression by C2 status and proviral category. Since the text focuses on the interpretation in C2 carriers, it would improve clarity to briefly describe the pattern in C2 non-carriers as well. Additionally, because intact-provirus cells can show lower (or not higher) HLA-C relative to the broader HIV-DNA+ pool in some strata, a short discussion of potential biological and/or technical contributors (e.g., differences in infected-cell composition/activation state, history of viral gene expression, or classification/coverage bias) would be valuable.

We thank Reviewer #6 for this helpful comment. We have adjusted our language and now point out that reduced HLA-C surface expression during early stages of ART initiation in HLA-C1 carriers may represent a specific adjustment to HIV-specific T cells, which may represent the major immunological threat during untreated and early stages of treated HIV infection. In contrast, during long-term ART, cells carrying intact proviruses in HLA-C2 carriers express higher levels of HLA-C, which is best explained as an adaptation to NK cell immune pressure and supports our conclusion that NK cells represent the dominant driver of immune selection at that time. We have modified the manuscript text to address this.

D. Appropriate use of statistics and treatment of uncertainties

Except as noted above, statistics and uncertainties were treated well.

E. Conclusions: robustness, validity, reliability

Conclusions were supported except as noted above.

F. Suggested improvements: experiments, data for possible revision

Clarifications as noted above including new analyses. Blocking experiments could be consider but not necessary. Mostly analyses.

G. References: appropriate credit to previous work?

yes

H. Clarity and context: lucidity of abstract/summary, appropriateness of abstract, introduction and conclusions

Very clear
